# A taxonomic revision of *Herminium* L. (Orchidoideae, Orchidaceae)

**DOI:** 10.3897/phytokeys.79.11215

**Published:** 2017-04-19

**Authors:** Bhakta Bahadur Raskoti, André Schuiteman, Wei-Tao Jin, Xiao-Hua Jin

**Affiliations:** 1 State Key Laboratory of Systematic and Evolutionary Botany, Institute of Botany, Chinese Academy of Sciences, Beijing 100093, China; 2 Science Directorate, Royal Botanical Gardens, Kew, Richmond, Surrey TW9 3AB, U.K.; 3 Southeast Asia Biodiversity Research Institute, Chinese Academy of Science, Yezin, Nay Pyi Taw 05282, Myanmar

**Keywords:** *Herminium tibeticum*, key, morphology, synonyms, taxonomy

## Abstract

*Herminium* (Orchidaceae, Orchidoideae) is a medium-sized genus widespread in the northern hemisphere, with a clear centre of diversity in the Himalayas. We present a comprehensive taxonomic revision of *Herminium* based on field observations and morphological studies, for which we examined about 2500 specimens. We recognize 49 species grouped into six formal sections, including one new species, *Herminium tibeticum*, from Tibet. We provide an identification key to the species, descriptions of the species, notes on ecology and distribution, and complete nomenclature for each species, including typifications. We here designate lectotypes for five species and reduce four taxa to synonymy.

## Introduction

**Figure 1.**
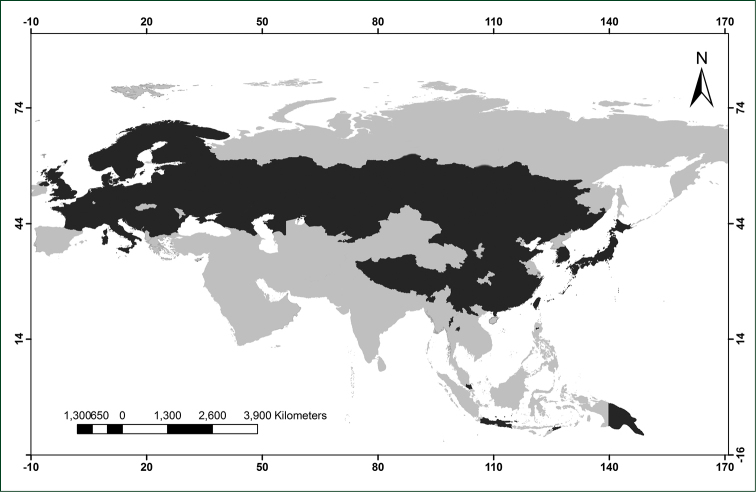
Geographical distribution of *Herminium*.

*Herminium* L. is a widely distributed genus, ranging from Europe through most of continental Asia to Japan, the Malay Archipelago and New Guinea (Fig. 1). However, most species are restricted to continental Asia, in particular to the Pan-Himalayan region, with only one, non-endemic species each in Europe (*H. monorchis*) and tropical Southeast Asia, including New Guinea (*H. lanceum*) (Hooker 1890; Tang and Wang 1937, 1940; Lang 1988; King and Pantling 1898; Press et al. 2000; Pridgeon et al. 2001; Pearce and Cribb 2002; Chen et al. 2009; Averyanov and Averyanova 2003; Averyanov 2010; Kurzweil 2010; Lee et al. 2013; Kurzweil and Lwin 2014).

In most of the earlier, floristic literature, such as Duthie (1906), Hooker (1890), King and Pantling (1898), and Tang and Wang (1937, 1940), *Herminium* was accepted in a broad sense. More recent authors, such as Seidenfaden (1977), Hara et al. (1978), Hunt (1970), Lang (1988), Pridgeon et al. (2001), Agrawala et al. (2010) and Ormerod (2013) took a more narrow view to the genus and recognized a number of small satellite genera, including *Androcorys* Schltr., *Bhutanthera* J. Renz, *Frigidorchis* Z.J. Liu & S.C. Chen, and *Porolabium* T. Tang & F.T. Wang. Raskoti et al. (2016) found that these satellite genera as well as certain misplaced members of *Peristylus* Blume and *Platanthera* Rich. were nested within *Herminium* s.l. This led these authors to propose a broader delimitation of the genus, which we also adopt in this revision.

As redefined, *Herminium* is a genus of 49 species of predominantly terrestrial orchids in the subtribe Orchidinae, characterized by having a (sub)globose tuber, a lip with a concave or spurred base, and pollinia with reduced caudicles. Unlike in the closely related genus *Peristylus*, the stigma-lobes are not adnate to the base of the lip. Most species have quite small, greenish, whitish or yellowish flowers arranged in racemes. Apart from regional treatments (e.g. Chen et al. 2009, Pearce and Cribb 2002, Pradhan 1976), no modern revision of the genus as a whole has been undertaken before. Here we present such a revision, which should make it easier to identify these inconspicuous orchids.

## Materials and methods

Vegetative as well as reproductive characters of *Herminium* were examined in the natural habitats and in herbaria. Herbarium specimens collected during our fieldwork were deposited in the Chinese National Herbarium (PE) and the National Herbarium and Plant Laboratories in Nepal (KATH). Flowers were preserved in formalin-acetic-acid-alcohol (FAA) for further study. Such material was studied in the laboratory under a stereomicroscope. Photographs and measurements were taken using image software of Nikon Instruments Inc., NIS-Elements D 3.10.

Specimens deposited at CAL, CDBI, DD, E, HNWP, KATH, K, KUN, LINN, PE, SIN, TI, and TAIF herbaria were examined. Photographs of the specimens were also studied from several herbaria (such as NY, P, UPS, W) where direct access was not possible. Over 2500 specimens, including types of most species, from the distributional range of *Herminium* have been examined.

Vegetative characters, such as tuber, leaf shape, phyllotaxy, and peduncle scales, and reproductive characters such as sepals, petals, lip and column, were examined. Flowers of dried specimens were rehydrated in boiling water prior to dissection. Floral characters such as bracts, sepals, petals, lip, and column were observed and measured with the help of a stereomicroscope. Literature, including Hooker (1890), King and Pantling (1898), Tang and Wang (1937, 1940), Pradhan (1976), Lang (1988), Press et al. (2000), Pearce and Cribb (2000; 2001), Boufford (2003), Chen et al. (2009), Agrawala et al. (2010), Averyanov (2010), Raskoti (2012, 2013), Ormerod (2013), Kurzweil (2010), and Kurzweil and Lwin (2014), was consulted for general distribution data and to assess some morphological characters of species we have been unable to examine ourselves. Thirty-seven morphological characters were compared (see Raskoti et al. 2016), and a set of diagnostic characters was chosen for the preparation of a dichotomous key. Based on phylogenetic analyses (Raskoti et al. 2016) and comparison of morphological characters with its relatives, a new species (*Herminium tibeticum*) is described and illustrated (see taxonomic treatment).

Habitat information is based on field observations, literature and herbarium labels. The distribution map was produced using DIVA GIS 7.5 (www.diva-gis.org) based on labels of representative specimens as well as data in the literature (e.g., Pearce and Cribb 2002, and Chen et al. 2009). Phenology is based on field observations of authors, information of lables and data in the literature (e.g., Pearce and Cribb 2002, and Chen et al. 2009)

### General morphology

**Tuber**: Unbranched, smooth, globose to oblong or ovoid. Slender roots are produced from the stem just above the tuber. The simple, rounded tuber in *Herminium* represents a major difference with the subterranean parts of Eurasian species of *Platanthera*, in which a tuber is either absent or is drawn out into a tapering, root-like tip (Fig. 4).

**Figure 2.**
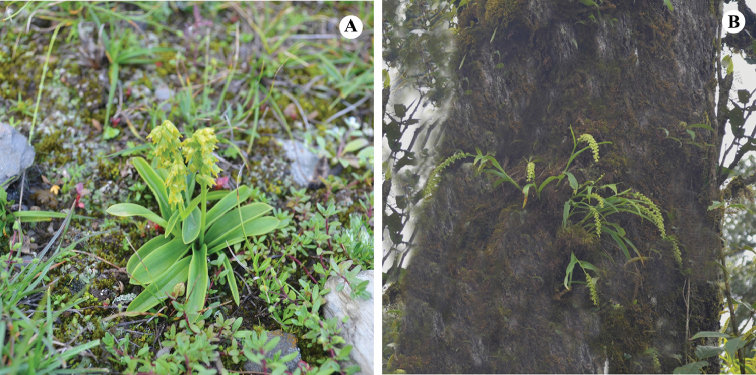
Habitat of *Herminium*. **A** *H. chloranthum* (Terrestrial) **B** *H. quinquelobum* (Epiphytic on tree trunk).

**Figure 3.**
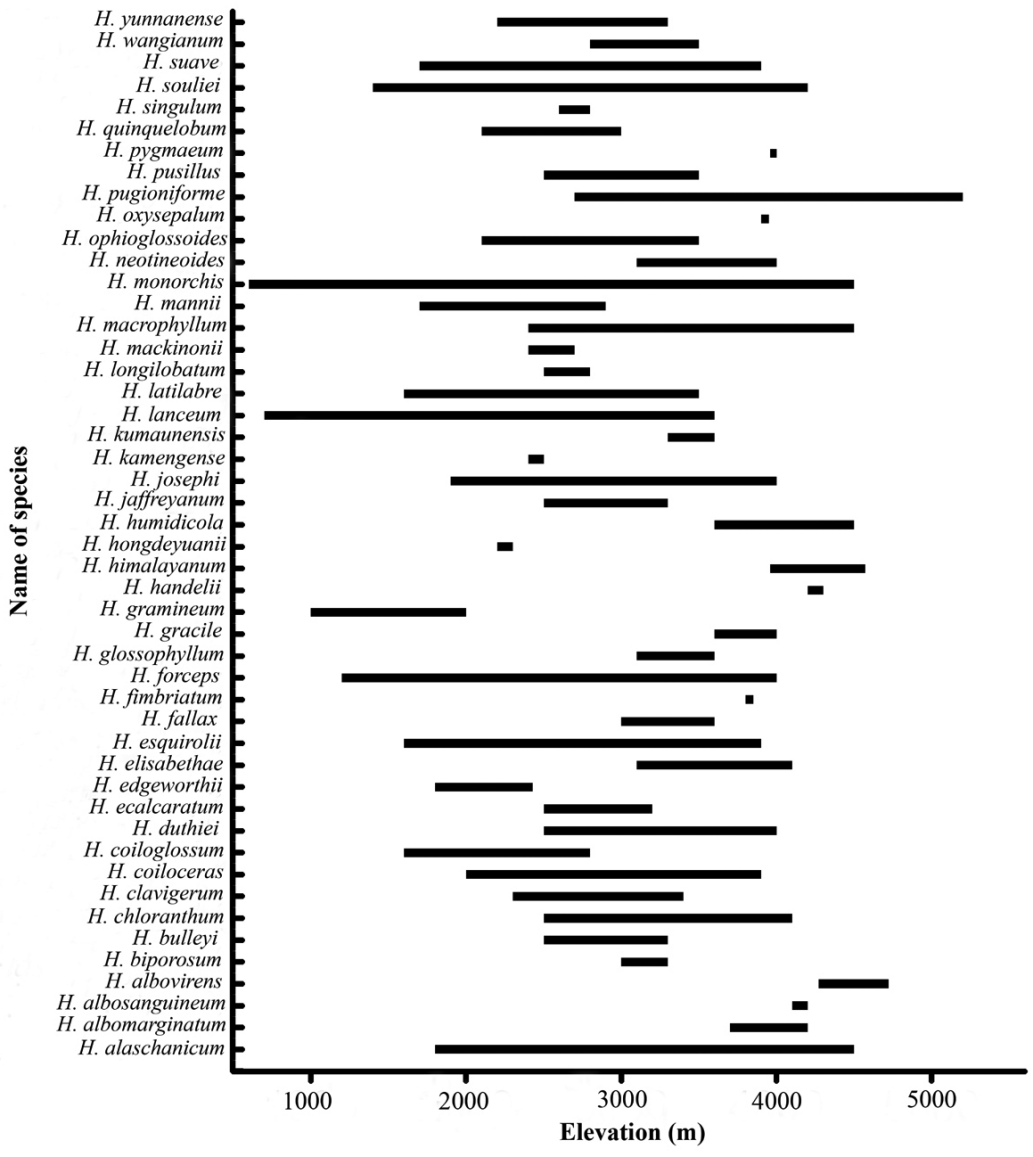
Elevational range of *Herminium*.

**Figure 4.**
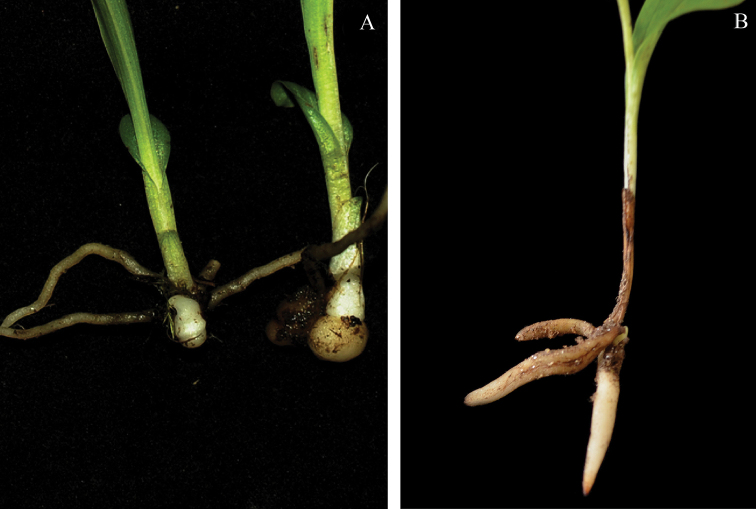
Subterranean parts of *Herminium* and *Platanthera*. **A** *Herminium* sp. **B** *Platanthera* sp.

**Phyllotaxy**: Leaf number and arrangement vary in *Herminium*. Leaves can be single or paired, or there can be more than two leaves; they can be basal or cauline, opposite, subopposite or spirally arranged. Except for *H. monophyllum* and *H. monorchis*, most of the subtropical and temperate species have several cauline leaves (e.g. *Herminium lanceum*, *Herminium longilobatum*, *Herminium clavigerum*) whereas subalpine and alpine species have only one or two basal leaves.

**Leaves**: Leaf shape in *Herminium* varies from linear, oblong, lanceolate, ovate, ovate-orbicular to sphathulate; the basal part always forms an amplexicaul sheath; the leaf apex can be obtuse, acute or acuminate. Linear leaves are restricted to subtropical and temperate species (*H. lanceum*, *H. longilobatum*, *H. mackinnonii*, *H. quinquelobum*); however; some subtropical and temperate species have ovate to oblong leaves (*H. latilabre*, *H. clavigerum*). Leaves may be shortly petiolate (*H. humidicola*), but are usually sessile.

**Stem**: Erect, glabrous. The stem may be abbreviated as in *H. humidicola*, *H. handelii*, and *H. albomarginatum* or elongate as in *H. clavigerum*, *H. edgeworthii*, and *H. lanceum*. The stem is covered by tubular sheaths but these do not produce a pseudostem, except in *H. humidicola*.

**Inflorescence**: This is almost always an unbranched raceme but in *H. humidicola* it is subcorymbose. The peduncle is cylindrical and smooth, except for the winged peduncle in *H. hongdeyuanii*. Most species have an elongate, densely many-flowered rachis (e.g. *H. clavigerum*, *H. longilobatum*, *H. lanceum*), but some, such as *H. humidicola* and *H. albomarginatum* have a short rachis with few, sometimes laxly arranged flowers. The peduncle may be shorter than the rachis or sometimes much longer.

**Peduncle scales**: In the subtropical and temperate species of *Herminium*, foliaceous peduncle scales are present (e.g. *H. lanceum*, *H. latilabre*, *H. edgeworthii*, *H. fallax*) but in several subalpine and alpine species, such as *H. pugioniforme*, *H. josephi*, and *H. fimbriatum*, they are absent.

**Floral bracts**: Most species have floral bracts, except *Herminium humidicola*, in which they are lacking. The shape of the floral bracts shows some variation. They can be ovate or triangular as in *H. albomarginatum*, *H. oxysepalum*, and *H. chloranthum*; reduced to tiny scales as in *H. pugioniforme* and *H. biporosum*; or they can be lanceolate as in *H. monorchis*, *H. lanceum*, and *H. fallax*. Floral bracts may be (sub) equal to the ovary as in *H. josephi*, *H. himalayanum* or distinctly shorter, or longer, as in *H. clavigerum*.

**Ovary**: Like all orchids, *Herminium* has an inferior ovary, which may be sessile or pedicelate, straight to arcuate, or twisted as in *H. biporosum*. Its shape ranges from cylindrical to fusiform. The ovary is usually beaked (abruptly tapered) at the apex. In *Herminium monorchis* and closely related species (*H. ophioglossoides*, *H. pygmaeum*) the ovaries are beaked and decurved.

**Figure 5.**
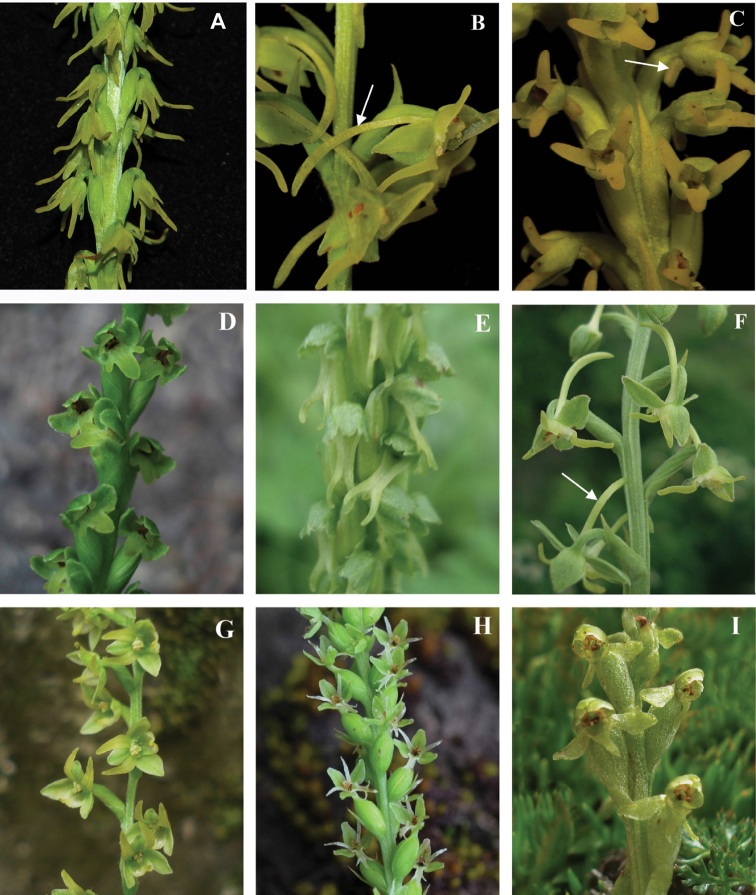
Flowers of representive species of *Herminium*. **A** *H. monorchis* **B** *H. latilabre* **C** *H. forceps* **D** *H. fallax* **E** *H. lanceum* **F** *H. edgeworthii* **G** *H. monophyllum* **H** *H. quinquelobum* **I** *H. pugioniforme*.

**Flower**: Most species of *Herminium* have inflorescence with spirally arranged flowers, but several have subsecund flowers, e.g. *H. monophyllum* (Fig. 5). The flowers are resupinate (rarely not resupinate), small, usually pale to yellowish green as in *Herminium monorchis*, *H. chloranthum*, but sometimes white (*H. coeloceras*, forms of *H. macrophyllum*), whitish-green (e.g. *H. mackinnonii*, *H. jaffreyanum*), or rarely more colorful, as in the white-and-red *H. albosanguineum*. In some species the flowers open only slightly (e.g. *H. humidicola*).

**Figure 6.**
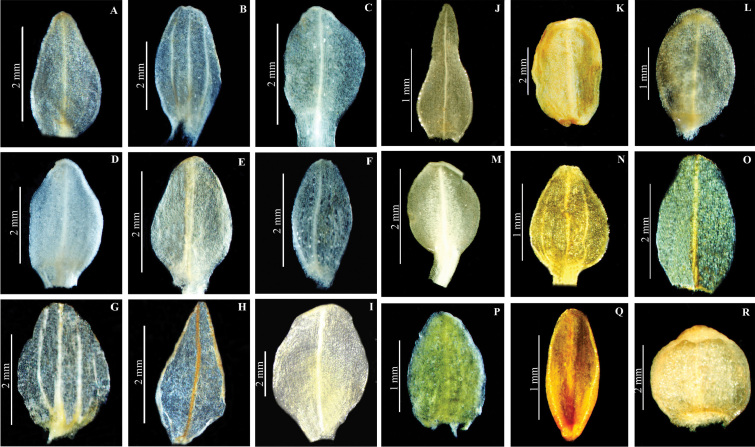
Dorsal sepal of *Herminium*. **A** *H. monorchis* **B** *H. chloranthum* **C** *H. pygmaeum* **D** *H. josephi* **E** *H. monophyllum* **F** *H. lanceum* **G** *H. macrophyllum* **H** *H. glossophyllum* **I** *H. coeloceras* **J** *H. fallax* **K** *H. handelii* **L** *H. elisabethae* **M** *H. himalayanum* **N** *H. clavigerum* **O** *H. quinquelobum* **P** *H. macrophyllum* **Q** *H. mannii* **R** *H. biporosum.*

**Dorsal sepal**: Always concave. It can be ovate (e.g. *H. josephi*, *H. souliei*, *H. alaschanicum*, *H. macrophyllum*), elliptic to ovate-oblong (e.g. *H. fallax*, *H. latilabre*, *H. monorchis*, *H. quinquelobum*), or lanceolate-oblong (*H. quinquelobum*, *H. ophioglossoides*, *H. glossophyllum*) (Fig. 6). Some alpine species (e.g. *H. humidicola* and *H. biporosum*) have a nearly orbicular dorsal sepal. The dorsal sepal may be spreading, as in *H. monophyllum* and *H. mackinnonii*, or connivent with the lateral sepals or petals forming a hood, as in *H. biporosum*, *H. lanceum*, *H. chloranthum*, *H. elisabethae*, *H. fallax*, and *H. alaschanicum*.

**Figure 7.**
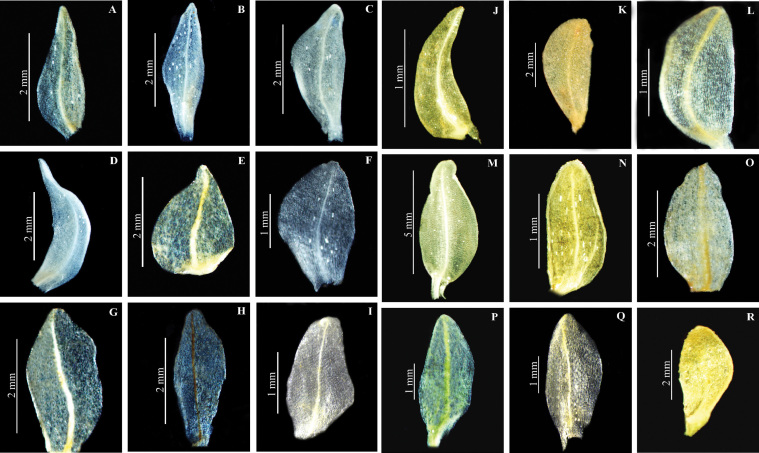
Lateral sepal of *Herminium*. **A** *H. monorchis* **B** *H. chloranthum* **C** *H. pygmaeum* **D** *H. josephi* **E** *H. monophyllum* **F** *H. lanceum* **G** *H. macrophyllum* **H** *H. glossophyllum* **I** *H. coeloceras* **J** *H. fallax* **K** *H. gracile* **L** *H. elisabethae* **M** *H. himalayanum* **N** *H. clavigerum* **O** *H. quinquelobum* **P** *H. macrophyllum* **Q** *H. lanceum* **R** *H. biporosum*.

**Lateral sepals**: The shape of lateral sepals is rather variable: oblong-obovate (*H. monorchis*), ovate-oblong (*H. lanceum*), ovate-lanceolate (*H. fallax* and *H. alaschanicum*), oblong-lanceolate (*H. macrophyllum*), ovate (*H. latilabre* and *H. elisabethae*), ovate to elliptic (*H. pugioniforme*), obliquely elliptic (*H. chloranthum*), or elliptic-oblong to elliptic-lanceolate (*H. clavigerum*) (Fig. 7). In most species, lateral sepals are spreading but they are completely reflexed in *H. clavigerum*, *H. latilabre*, *H. edgeworthii*, and *H. pugioniforme*; in *H. gracile* they are reflexed so as to become parallel to the lip. In *H. latilabre*, *H. edgeworthii*, and *H. clavigerum*, the lateral sepals have ciliate margins, whereas these are denticulate in *H. oxysepalum*.

**Figure 8.**
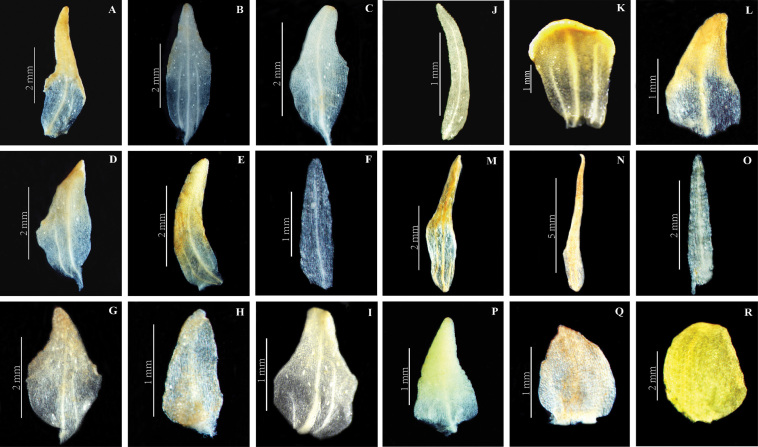
Petal of *Herminium*. **A** *H. monorchis* **B** *H. chloranthum* **C** *H. pygmaeum* **D** *H. josephi* **E** *H. monophyllum* **F** *H. lanceum* **G** *H. josephi* **H** *H. coiloglossum* **I** *H. coeloceras* **J** *H. fallax* **K** *H. himalayanum* **L** *H. elisabethae* **M** *H. alaschanicum* **N** *H. ophioglossoides* **O** *H. quinquelobum* **P** *H. macrophyllum* **Q** *H. ecalcaratum* **R** *H. biporosum*

**Petals**: Connivent with the dorsal sepals, except in *H. jaffreyanum*, *H. monophyllum*, *H. quinquelobum* and some other species, which have free, spreading petals. Their shape varies considerably: linear (*H. fallax*, *H. lanceum*, *H. souliei*, *H. quinquelobum*, *H. mackinnonii*, *H. jaffreyanum*, *H. monophyllum*), lanceolate (*H. chloranthum*, *H. pygmaeum*), oblong-lanceolate (*H. ophioglossoides*), ovate-lanceolate (*H. macrophyllum*, *H. josephi*, *H. elisabethae*), ovate to broadly ovate (*H. biporosum*, *H. coeloceras*, *H. macrophyllum*, *H. ecalcaratum*), oblong (*H. coiloglossum*), rhombic (*H. monorchis*), or spathulate (*H. himalayanum*) (Fig. 8).

**Figure 9.**
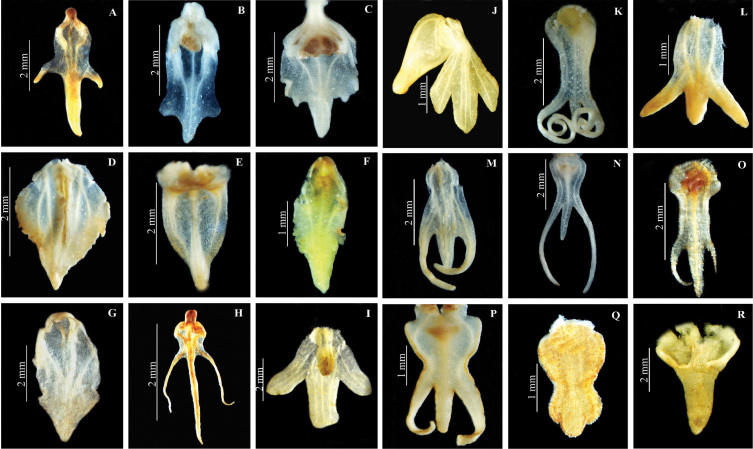
Lip of *Herminium*. **A** *H. monorchis* **B** *H. chloranthum* **C** *H. pygmaeum* **D** *H. josephi* **E** *H. monophyllum* **F** *H. macrophyllum* **G** *H. macrophyllum* **H** *H. alaschanicum* **I** *H. coeloceras* **J** *H. fallax* **K** *H. longilobatum* **L** *H. elisabethae* **M** *H. alaschanicum* **N** *H. lanceum* **O** *H. quinquelobum* **P** *H. kamengense* **Q** *H. coiloglossum* **R** *H. biporosum*.

**Lip**: Usually 3-lobed and with a concave and ecallose basal part that may be dilated as in *H. coiloglossum*, *H. lanceum* or not dilated as in *H. alaschanicum*. Some species, such as *H. coeloceras*, have a callus on the lip. In a few species the lip is entire, as in *H. monophyllum*, or 5-partite, as in *Herminium quinquelobum*. The shape of the lip may be ovate (*H. josephi*), oblong (*H. lanceum*), lingulate (e.g. *H. biporosum*, *H. latilabre*), or ovate-lanceolate (e.g. *H. macrophyllum*) (Fig. 9).

**Spur**: Lacking in most of the species, but a significant minority have a spurred lip (*H. alaschanicum*, *H. albomarginatum*, *H. albosanguineum*, *H. albovirens*, *H. bulleyi*, *H. clavigerum*, *H. edgeworthii*, *H. quinquelobum*, *H. fallax*, *H. fimbriatum*, *H. forceps*, *H. handelii*, *H. himalayanum*, *H. humidicola*, *H. latilabre*, *H. mannii*, *H. singulum*, and *H. suave*). The spur may be cylindrical (e.g. *H. edgeworthii*) or scrotiform (*H. alaschanicum*, *H. quinquelobum*, *H. fallax*). It is usually shorter than the ovary (e.g. *H. alaschanicum*, *H. quinquelobum*, *H. forceps*) but in a few species the spur is longer than the ovary (e.g. *H. latilabre*, *H. edgeworthii*) (Fig. 5). In some species, such as *H. josephi*, *H. monorchis*, *H. ophioglossoides*, and *H. pygmaeum*, the lip has a saccate base, which can be almost like a spur.

**Figure 10.**
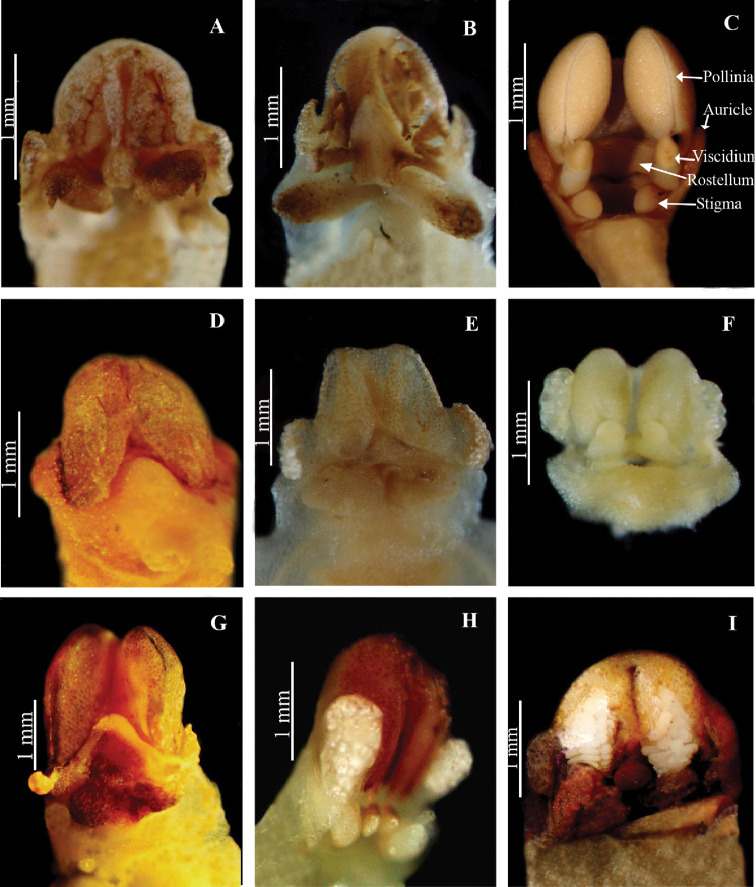
Column structure in *Herminium*. **A** *H. monorchis* **B** *H. josephi* **C** *H. latilabre* **D** *H. monophyllum* **E** *H. kamengense* **F** *H. longilobatum* **F** *H. himalayanum* **H** *H. mannii* **I** *H. fallax*.

**Figure 11.**
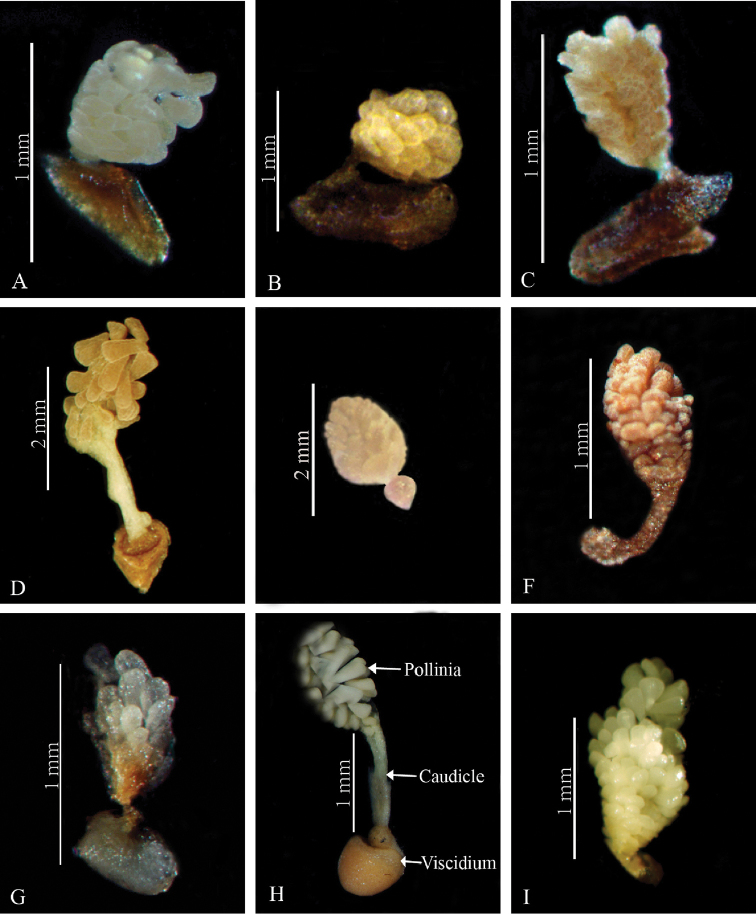
Pollinia structure in *Herminium*. **A** *H. josephi* **B** *H. macrophyllum* **C** *H. pygmaeum* **D** *H. handelii* **E** *H. lanceum* **F** *H. fallax* **G** *H. forceps* **H** *H. latilabre* **I** *H. coeloceras*.

**Column**: Short but well-developed (Fig. 10). The anther is adnate to the column apex, 2-locular, with the locules parallel or divergent. Pollinia 2, oblong to ellipsoid, granular-farinaceous and sectile, with very short or vestigial caudicles. Viscidia globose (*H. lanceum*, *H. longilobatum*) or often involute, naked and hornlike (*H. alaschanicum*, *H. josephi*, *H. monorchis*) (Fig. 11). Rostellum triangular, often with arms. Stigma lobes 2, free, sometimes confluent, usually clavate and raised, in some species, such as *H. albomarginatum* and *H. pugioniforme*, pulvinate; in *H. latilabre* and closely related species the stigma lobes are extended along the entrance of the spur, but not adnate to the lip base as in *Peristylus*. There are two, usually prominent auricles laterally at the base of the anther.

**Fruits**: A capsule; longitudinally dehiscent; usually erect, beaked, oblong or fusiform, in some species it may be twisted.

### Ecology

Almost all species of *Herminium* are terrestrial, but a few, such as *H. lanceum* (Thunb. ex Sw.) Vuijk, *H. mackinnonii* Duthie and *H. quinquelobum* King & Pantl., occasionally grow as epiphytes on tree trunks or major branches of large trees (Fig. 2). Forests, woodlands and grasslands are a common habitat of *Herminium*, where the species grow in humus-rich soil in light shade. Many species are subalpine or alpine, growing fully exposed in high mountain valleys and meadows (e.g. *H. albomarginatum*, *H. josephi*, *H. monorchis*, *H. pugioniforme*) or on peaty soil in moraine areas (e.g. *H. alaschanicum*, *H. chloranthum*).

*Herminium pugioniforme* appears to reach the highest elevation of any orchid, said to occur up to 5500 m (Pridgeon et al. 2001; but see note under *H. pugioniforme* below). In contrast, *H. monorchis* grows at sea level in part of its range, e.g. in The Netherlands. In tropical and subtropical regions, all *Herminium* species are mountain plants, with many species growing at an elevation of around 4000 m (Fig. 3). A few species, such as *H. lanceum*, *H. souliei* and *H. monorchis*, have a wide distributional range, but many more, about one quarter of the species, are narrow endemics, including *H. albosanguineum*, *H. handelii*, *H. kamengense*, *H. oxysepalum*, and *H. pygmaeum*.

Little, if anything, is known about the pollination of nearly all species of *Herminium*. Only that of *H. monorchis* has been thoroughly studied (Nilsson 1979; Claessens and Kleynen 2016). The honey-scented flowers of this taxon produce small quantities of nectar and attract various species of minute insect, in particular parasitic wasps, flies, mosquitos and even beetles (Claessens and Kleynen 2016). The most important pollinators in Sweden were found to be female parasitic wasps of the genus *Tetrastichus* (Nilsson, 1979), which accounted for 65% of pollinia transfers, with other parasitic wasps accounting for 12.2%, while Diptera only accounted for 7.8%. Most *Herminium* species are superficially similar to *H. monorchis*, in that they have small greenish flowers, and they may have similar pollinators. A few species with longer spurs and larger, *Platanthera*-like flowers (here included in the new section Pseudoplatanthera), may well be moth-pollinated.

### Phenology

In the Pan-Himalayan region, vegetative growth of *Herminium* species starts in March–May and flower buds appear in June. Flowering begins in June and reaches its peak in August. Fruiting occurs largely simultaneously with the flowering time. Leaf and stem decay in November–December. In the middle of winter, i.e. during December to February, plants of *Herminium* are dormant, surviving as subterranean tubers.

## Taxonomic treatment

### HERMINIUM

Taxon classificationPlantaeAsparagalesOrchidaceae

 Opera Var.: 251. 1758.

Androcorys Schltr., Repert. Spec. Nov. Regni Veg. Beih. 4: 52. 1919. Type species: Androcorys ophioglossoides Schltr.
Bhutanthera J. Renz, Edinburgh J. Bot. 58: 99. 2001. Type species: Bhutanthera albomarginata (King & Pantl.) J.Renz
Cybele Falc. in Lindl., Veg. Kingd., ed. 2: 183c. 1847, *nom. inval.*
Frigidorchis Z.J. Liu & S.C. Chen, J. Fairylake Bot. Gard. 6: 14. 2007. Type species: Frigidorchis humidicola Z.J. Liu & S.C. Chen
Monorchis Ség., Pl. Veron. 3: 251. 1754. Type species: Monorchis herminium O.Schwarz
Porolabium T. Tang & F.T. Wang, Bull. Fan Mem. Inst. Biol., Peiping, Bot. Ser. 10: 36. 1940. Type species: Porolabium biporosum (Maxim.) Tang & F. T. Wang
Thisbe Falc. in Lindl., Veg. Kingd., ed. 2: 183c. 1847, *nom. inval.*

#### Type species.

*Herminium monorchis* (L.) R.Br.

#### Description.

Herbs; terrestrial or occasionally epiphytic. Tubers unbranched, globose to oblong-ellipsoid. Stem erect, one-to several-leaved. Leaves basal to cauline. Inflorescence racemose or rarely subcorymbose. Flowers erect, horizontal, or nodding; ovary and pedicel straight or arcuate, beaked. Sepals subequal; dorsal sepal connivent with petals and forming a hood; lateral sepals spreading, oblique. Petals usually thickened and fleshy; lip base shallowly concave, saccate or shortly spurred. Column very short; pollinia 2, granular-farinaceous, sectile, each attached to a viscidium via caudicle; viscidia often involute and hornlike, naked; rostellum triangular, with arm-like lobes; stigma lobes 2, free, raised, clavate or pulvinate; staminodes 2, usually prominent. Fruit usually erect, oblong to fusiform.

### Infrageneric classification

Based on the phylogeny in Raskoti et al. (2016) and previous classifications of Lang (1988), Pridgeon et al. (2001), and Agrawala et al. (2010), we here propose the following infrageneric classification.

#### I. Herminium sect. Herminium

Taxon classificationPlantaeAsparagalesOrchidaceae

##### Note.

Ovary rostrate, with the apex hook-like bent sideways or downwards (not always clearly so in *H. monophyllum*). Lip with or without a short spur, entire or lobed.

##### Type species.

*Herminium monorchis* (L.) R.Br.

##### Species.

1. *H. alaschanicum*, 6. *H. bulleyi*, 7. *H. chloranthum*, 9. *H. coeloceras*, 13. *H. elisabethae*, 16. *H. forceps*, 17. *H. glossophyllum*, 24. *H. josephi*, 27. *H. kumaunense*, 32. *H. macrophyllum*, 33. *H. mannii*, 34. *H. monophyllum*, 35. *H. monorchis*, 36. *H. neotineoides*, 37. *H. ophioglossoides*, 42. *H. pygmaeum*, 43. *H. singulum*, 46. *H. tangianum*, 47. *H. tibeticum*.

#### II. Herminium sect. Thisbe X.H. Jin, Schuit. & Raskoti

Taxon classificationPlantaeAsparagalesOrchidaceae

urn:lsid:ipni.org:names:77161966-1

##### Note.

Ovary not with hooked-rostrate apex; lip not spurred, 2-, 3-, or 5-lobed.

##### Type species.

*Herminium lanceum* (Thunb. ex Sw.) Vuijk, here designated.

##### Species.

10. *H. coiloglossum*, 11. *H. ecalcaratum*, 21. *H. hongdeyuanii*, 23. *H. jaffreyanum*, 26. *H. kamengense*, 28. *H. lanceum*, 30. *H. longilobatum*, 31. *H. mackinnonii*, 42. *H. quinquelobum*, 45. *H. souliei*, 49. *H. yunnanense*.

#### III. Herminium sect. Androcorys

Taxon classificationPlantaeAsparagalesOrchidaceae

(Schltr.) X.H. Jin, Schuit. & Raskoti stat. nov.

urn:lsid:ipni.org:names:77161967-1

##### Basionym.

*Androcorys* Schltr., Repert. Spec. Nov. Regni Veg. Beih. 4: 52. 1919.

##### Note.

Ovary not with hooked-rostrate apex; lip not spurred, entire, usually abruptly dilated at the base; anther-connective forming a hood over the thecae.

##### Type species.

*Androcorys ophioglossoides* Schltr. (= *Herminium gracile* King & Pantl.)

##### Species.

5. *H. biporosum*, 18. *H. gracile*, 25. ?*H. kalimpongense*, 38 *H. oxysepalum*, 39. *H. pugioniforme*, 40. *H. pusillum*, 48. *H. wangianum*.

##### Note.

The aberrant *H. kalimpongense* Pradhan may not belong to this section (see discussion under *Herminium kalimpongense*).

#### IV. Herminium sect. Bhutanthera

Taxon classificationPlantaeAsparagalesOrchidaceae

(J.Renz) X.H. Jin, Schuit. & Raskoti stat. nov.

urn:lsid:ipni.org:names:77161968-1

##### Basionym.

*Bhutanthera* J. Renz, Edinburgh J. Bot. 58: 99. 2001.

##### Note.

Ovary not with hooked-rostrate apex; lip spurred, 3-lobed; stigma cushion-like, not projecting beyond rostellum.

##### Type species.

*Herminium albomarginatum* (King & Pantl.) X.H. Jin, Schuit., Raskoti & L.Q. Huang

##### Species.

2. *H. albomarginatum*, 3. *H. albosanguineum*, 4. *H. albovirens*, 15. *H. fimbriatum*, 19. *H. handelii*, 20. *H. himalayanum*, 22. *H. humidicola*.

#### V. Herminium sect. Cybele X.H. Jin, Schuit. & Raskoti, sect. nov.

Taxon classificationPlantaeAsparagalesOrchidaceae

urn:lsid:ipni.org:names:77161969-1

##### Note.

Plant 1-leaved; ovary not with hooked-rostrate apex; lip spurred, 3-lobed; stigma lobes projecting beyond rostellum.

##### Type species.

*Herminium fallax* (Lindl.) Hook. f., here designated.

##### Species.

14. *H. fallax*.

#### VI. Herminium sect. Pseudoplatanthera X.H. Jin, Schuit. & Raskoti

Taxon classificationPlantaeAsparagalesOrchidaceae

urn:lsid:ipni.org:names:77161970-1

##### Note.

Plant 2- or more-leaved; ovary not with hooked-rostrate apex; lip spurred, entire; stigma lobes projecting beyond rostellum.

##### Type species.

*Herminium clavigerum* (Lindl.) X.H. Jin, Schuit., Raskoti & L.Q. Huang, here designated.

##### Species.

8. *H. clavigerum*, 12. *H. edgeworthii*, 29. *H. latilabre*, 45. *H. suave*.

### Key to the species

**Table:** 

1	Ovary apex hooked sideways or downward, distinctly rostrate	**2**
–	Ovary apex straight or not distinctly rostrate	**20**
2	Plant with one or more leaves; lip with concave or saccate to globose base	**3**
–	Plant with two or more leaves; lip with distinct clavate or cylindrical spur	**15**
3	Lip entire or lateral lobes obscure	**4**
–	Lip 3-lobed with distinct lateral lobes	**7**
4	Plant with single leaf; lip without callus	**34. *H. monophyllum***
–	Plant with two or more leaves; lip with or without callus	**5**
5	Lip pandurate	**46. *H. tangianum***
–	Lip triangular to ovate	**6**
6	Flower green; lip deflexed, 3.5–5 mm long, in basal half of the blade with two parallel longitudinal keels; sac reduced to a slight bulge at the base of the lip	**24. *H. josephi***
–	Flower white or green; lip subpatent to deflexed, 1.6–3.5 mm long, ecallose or with a single callus on the blade; sac subglobose	**32. *H. macrophyllum***
7	Lip pandurate or subpandurate	**8**
–	Lip not pandurate	**11**
8	Petal constricted in the middle, lip lobes obscure	**27. *H. kumaunense***
–	Petal not constricted in the middle, lip with distinct lobes	**9**
9	Leaves oblong, elliptic or spathulate, petal elliptic-lanceolate, lip fiddle-shaped	**7. *H. chloranthum***
–	Leaves linear-oblong or lanceolate, petal trullate, lip not fiddle-shaped	**10**
10	Leaves linear-oblong, peduncle with lanceolate peduncle-scales, lip without callus	**42. *H. pygmaeum***
–	Leaves lanceolate, peduncle without peduncle-scales, lip with two longitudinal calli	**47. *H. tibeticum***
11	Flower white or green; lip with a large callus	**12**
–	Flower yellowish green; lip without callus	**13**
12	Lip lateral lobes oblong, callus not papillose	**9. *H. coeloceras***
–	Lip lateral lobes lanceolate, callus papillose	**36. *H. neotineoides***
13	Petals not abruptly narrowed above the middle, lip base with a tiny globose spur	**17. *H. glossophyllum***
–	Petal abruptly narrowed above the middle, lip base lacking spur	**14**
14	Plant with two or more leaves, petals rhombic-caudate, c. 3 times longer than wide, lip side-lobes about half as long as the mid-lobe	**35. *H. monorchis***
–	Plant with one leaf, petals lanceolate-caudate, c. 6–8 times longer than wide, lip side-lobes about 3/4 as long as the mid-lobe	**37. *H. ophioglossoides***
15	Lip entire	**16**
–	Lip 3-lobed	**17**
16	Plant with more than one well-spaced oblong-lanceolate leaf, lateral sepals oblong-lanceolate	**16. *H. forceps***
–	Plant with a single basal elliptic leaf, lateral sepals obliquely ovate	**43. *H. singulum***
17	Leaves elliptic, oblong to lanceolate, petals ovate, oblique	**13. *H. elisabethae***
–	Leaves linear	**18**
18	Floral bracts longer than the ovary, petals oblong-lanceolate, abruptly narrowed above middle	**1. *H. alaschanicum***
–	Floral bracts shorter than the ovary	**19**
19	Spur scrotiform, shorter than ovary, petals ovate-lanceolate	**33. *H. mannii***
–	Spur cylindrical, longer than ovary and pedicel, petals oblong-lanceolate	**6. *H. bulleyi***
20	Lip not spurred, though possibly with a semiglobose saccate base, possibly dilated at base	**21**
–	Lip spurred, not dilated at base	**39**
21	Lip 2-, 3-, or 5-partite	**22**
–	Lip entire	**32**
22	Petals ovate	**23**
–	Petals linear or oblong	**24**
23	Plant with single basal linear-oblong leaf, flower yellowish-green	**49. *H. yunnanense***
–	Plants with two alternate oblong leaves, flower white	**11. *H. ecalcaratum***
24	Petals spreading, white	**25**
–	Petals connivent with dorsal sepal, yellowish green	**27**
25	Lip 5-lobed, lateral sepals oblong, petals linear	**42. *H. quinquelobum***
–	Lip 3-lobed, lateral sepals ovate-lanceolate or ovate-oblong, petals lanceolate or linear-lanceolate	**26**
26	Lateral lobes of lip reduced or sometimes absent, lateral sepals ovate-lanceolate	**23. *H. jaffreyanum***
–	Lateral lobes of lip distinctly developed, lateral sepals ovate-oblong	**31. *H. mackinnonii***
27	Lateral lobes of lip falcate	**28**
–	Lateral lobes of lip straight	**29**
28	Plant with single leaf, petals oblong	**10. *H. coiloglossum***
–	Plant with two or more leaves, petals linear	**45. *H. souliei***
29	Lip broadly ovate, petals slightly falcate	**26. *H. kamengense***
–	Lip oblong, petals not falcate	**30**
30	Lip with a callus, lateral lobes coiled at anthesis	**30. *H. longilobatum***
–	Lip without callus, lateral lobes not coiled	**31**
31	Peduncle not winged, lateral lobes longer than mid-lobe	**28. *H. lanceum***
–	Peduncle winged, lateral petals shorter than mid-lobe	**21. *H. hongdeyuanii***
32	Leaf cordate, fleshy, with reticulate markings, flower white	**25. *H. kalimpongense***
–	Leaf not cordate, not fleshy, without markings, flower greenish	**33**
33	Lip with two sharply delimited pits at base, or with concave basal part separated into two cavities by a longitudinal callus ridge	**34**
–	Lip without pits or cavities at base, possibly with two shallow depressions, but these not sharply delimited or separated by a callus ridge	**36**
34	Anther-channels elongate, pollinia with well-developed caudicles; lateral sepals deflexed, held parallel with the lip	**18. *H. gracile***
–	Anther channels very short, caudicles very short; lateral sepals patent, at about right angles to the lip	**35**
35	Inflorescence relatively lax: distance between at least some of the successive flowers about as long as or longer than the ovary; lateral sepals 3 mm long, lip 2.8–3 mm long	**5. *H. biporosum***
–	Inflorescence dense: distance between successive flowers less than half as long as the ovary; lateral sepals 1.5–1.8 mm long, lip 1.4–1.8 mm long	**39. *H. pugioniforme***
36	Margins of lateral sepals ciliate or dentate	**37**
–	Margins of lateral sepals glabrous and smooth	**38**
37	Lateral sepals ciliate, petals oblong	**40. *H. pusillum***
–	Lateral sepals dentate, petals oblong-lanceolate	**38. *H. oxysepalum***
38	Floral bracts shorter than the ovary; lip linear-ligulate, dilated at base	**48. *H. wangianum***
–	Floral bracts at least in the lower flowers as long as or longer than the ovary; lip ovate	**34. *H. monophyllum***
39	Stigma not cushion like, projecting beyond the rostellum; lip entire or 3-lobed	**40**
–	Stigma cushion like, situated underneath the rostellum; lip 3-lobed	**44**
40	Plant with one leaf, lip 3-lobed	**14. *H. fallax***
–	Plant with more than one leaf, lip entire, lingulate	**41**
41	Spur shorter than pedicel and ovary	**8. *H. clavigerum***
–	Spur equal to or longer than pedicel and ovary	**42**
42	Spur pointing upwards	**12. *H. edgeworthii***
–	Spur pointing downwards	**43**
43	Leaves ovate to oblong, apex acute, spur longer than ovary	**29. *H. latilabre***
–	Leaves linear, apex acuminate, spur equal to or shorter than ovary	**45. *H. suave***
44	Inflorescence subcorymbose, floral bracts absent	**22. *H. humidicola***
–	Inflorescence racemose, floral bract present	**45**
45	Lateral lobes of lip much reduced, distinctly shorter than the mid-lobe	**19. *H. handelii***
–	Lateral lobes of lip distinct, nearly equal to the mid-lobe	**46**
46	Leaves opposite, apex of the dorsal sepal fimbriate	**15. *H. fimbriatum***
–	Leaves subopposite or distant, apex of the dorsal sepal not fimbriate	**47**
47	Inflorescence 1–2-flowered	**48**
–	Inflorescence 3- or more-flowered	**49**
48	Sepals white, petals and lip red	**3. *H. albosanguineum***
–	Sepals green, petals greenish or white, lip white	**4. *H. albovirens***
49	Sepals white; inflorescence 3–4-flowered	**20. *H. himalayanum***
–	Sepals green with white margins; inflorescence 5–20-flowered	**2. *H. albomarginatum***

### 1. Herminium alaschanicum

Taxon classificationPlantaeAsparagalesOrchidaceae

Maxim., Bull. Acad. Imp. Sci. Saint-Pétersbourg 31: 105. 1887.

Figs 8M , 9M

Monorchis alaschanica (Maxim.) O. Schwarz, Mitt. Thüring. Bot. Ges. 1: 95. 1949.
Peristylus alaschanicus (Maxim.) N. Pearce & P.J. Cribb, Edinburgh J. Bot. 58: 117. 2001.

#### Type.

MONGOLIA. Mont. Alaschan, 1873, *Przewalski N.M., 163* (Holotype: LE! [LE01012203]; Isotype: K! [K000079038]).

#### Description.

Plant 13–68 cm tall. Tubers oblong, 1–1.5 × ca. 0.5–1 cm. Stem with 2 or 3 tubular sheaths at base, 3–4-leaved. Leaves linear-lanceolate, 2–15 × 0.4–1 cm, apex acuminate. Inflorescence 14–34 cm; peduncle cylindrical, with 3–5 lanceolate peduncle-scales 5–15 mm; rachis 4–8 cm, laxly many-flowered; floral bracts ovate-lanceolate, 5–26 mm, longer than ovary, apex cuspidate. Flowers green; pedicel and ovary twisted, beaked and much hooked toward apex, 5–15 mm. Dorsal sepal oblong-lanceolate, 3–3.5 × 1.2–3 mm, apex obtuse; lateral sepals oblong-lanceolate, 3–3.5 × 1.2–2 mm, apex subacute. Petals ovate-lanceolate, falcate, 3.2–5 × 1.1–1.5 mm, narrowed above middle, fleshy, apex cuspidate. Lip oblong, 4–5.5 × 1.2–1.5 mm, base concave, spurred, 3-lobed near middle; lateral lobes linear, 2.5 × 0.3 mm; mid-lobe linear-triangular, 1.5 × 0.5 mm, apex acute. Column 1 mm; pollinia obovoid; caudicles very short, viscidia involute, hornlike, rostellum triangular, armed, stigma transversely oblong, situated below the rostellum.

#### Flowering time.

June–September.

#### Habitat.

Terrestrial in *Quercus-Abies* forests, dry stony slopes, alpine meadows and valleys at elevations of 1800–4500 m.

#### Distribution.

China, Mongolia, Nepal.

#### Specimens examined.

CHINA: **Gansu**, Tianzhu county, Leigong mountain, 2800 m, 09.07.1964, *Sino-Soviet Team,758* (PE); Min Hsien, 2500 m, 07.07.1936, *Wang Z. B., 4879 A* (PE); **Hebei**, Laiyuan county, Paishihshan; **Inner Mongolia**, Xing county, 1700 m, 16.07.1977, *Zhao Y., 37* (PE); **Qinghai**, Nangqian county, 4300 m, 11.07.1965, *Yang Y., 01149A* (PE); **Sichuan**, Daofu county, Geka Town, Mugu, 3900 m, 26.08.2001, *Luo Y.B., 695* (PE); between Luhua and Gyangze along Highway 317, 3385 m, 24.07.2005, *Boufford D. E. et al., 33486* (K); **Shaanxi**, Wuqi county, 1800 m, 22.08.1956, *Huang Team, 8216* (PE); **Shanxi**, Shai-wang-ping, 1800 m, 24.07.1921, *Smith H., 6660* (PE); **Tibet**, Gyirong county, 4400 m, 31.07.1975, *Qinghai-Tibet Team, 3137* (PE); Lhasa, 8 km SSE of Lhasa, 4000 m, 03.08.1989, *Dickoré B., 3411* (K); Nyalam, 4200 m, 04.09.1972, *Tibetan Herb Survey Team, 1773* (PE); Radja and Yellow River Gorges, SW of Radja, 3350 m, 06.1926, *Rock J. F., 14210* (K);West side of Jinsha Jiang opposite Luoxu, 3440 m, 30.07.2005, *Boufford D. E. et al.*, 33797 (K); **Yunnan**, Gongshan county, 21.08.2007, *Jin X. H., 9199* (PE); Lijiang, 2800 m, 27.06.1937, *Yu T. T., 15224* (PE).

NEPAL: Mustang, Muktinath, 07.2007, *Raskoti B. B., 501* (KATH); same locality, 3700 m, 07.08.1974, *Joshie D. P. and Bhattacharya T. K., 2270/74* (K); 3 miles N of Shimen, 4300 m, 06.08.1973, *Grey-Wilson and Phillips, 538* (K).

### 2. Herminium albomarginatum

Taxon classificationPlantaeAsparagalesOrchidaceae

(King & Pantl.) X.H. Jin, Schuit., Raskoti & L.Q. Huang, Cladistics 2015.

Platanthera albomarginata (King & Pantl.) Kraenzl., Orchid. Gen. Sp. 1: 939. 1901.
Peristylus albomarginatus (King & Pantl.) K.Y. Lang, Acta Phytotax. Sin. 34: 640. 1996.
Bhutanthera albomarginata (King & Pantl.) Renz, Edinburgh J. Bot. 58: 101. 2001.

#### Basionym.

*Habenaria albomarginata* King & Pantl. in Ann. Roy. Bot. Gard. (Calcutta) 8: 322, t.425. 1898.

#### Type.

INDIA. Sikkim, Singalila, 13000 ft, 07.1896, *Pantling R., 450* (Holotype: K! [K000387527]).

#### Description.

Plant 10–14 cm tall. Tuber globose, 0.6–1.4 × 0.7–1.3 cm. Stem sheathed, 6.5–11 cm long, 2-leaved, sheaths tubular, ovate, obtuse, 2–3.5 × 0.6–0.7 cm. Leaves distant, orbicular-elliptic, obtuse shortly sheathing at base, 3–5 × 1.3–2.4 cm. Inflorescence 5–20-flowered, rachis 1–3 cm long, floral bracts lanceolate, 1–2 × 0.3–0.4 cm, upper bracts triangular, 1–1.5 mm long. Flower white, sepal green with white margin, ovary 4–6 mm, sessile, beaked. Dorsal sepal broadly elliptic-ovate, obtuse, 4 × 2.5 mm, lateral sepal elliptic-ovate, obtuse, 4 × 3 mm. Petal suborbicular, concave, 2 × 1 mm. Lip 3-lobed, 2 × 1 mm, lateral lobes oblong, mid-lobe oblong, obtuse, spur cylindrical, 2 mm long. Column 1.5 mm tall, pollinia clavate-elliptic, 1–1.2 mm, rostellum triangular, stigma pulvinate.

#### Flowering time.

July–September.

#### Habitat.

Terrestrial in alpine meadows and on grassy slopes at elevations of 3720–4270 m.

#### Distribution.

India, Nepal

#### Specimens examined.

INDIA: **Sikkim**, Nathang, 13.07.1877, *King, 4367* (K); Sikkim, Tsomgo Chho, 08.07.1996, *Long and Noltie, 71* (E); Sikkim, Yampung, 12.08.1913, *Rohmoo, 1098* (E).

NEPAL: **Bagmati**, Rasuwa, Above Rupche Bhanjyang, Ganesh Himal, 3600 m, 02.07.1992, *Baker W. J., Burkitt T. A., Miller J. T and Shrestha R., 9* (KATH).

### 3. Herminium albosanguineum

Taxon classificationPlantaeAsparagalesOrchidaceae

(Renz) X.H. Jin, Schuit., Raskoti & L.Q. Huang, Cladistics 2015.

#### Basionym.

*Bhutanthera albosanguinea* Renz, Edinburgh J. Bot. 58: 101. 2001.

#### Type.

BHUTAN. Thimphu district, Darkey Pang Tso, north of the Paro, *Wood J.R., 7405* (Holotype: E! [E00008001]).

#### Description.

Plant 4.5 cm tall. Tuber not seen. Stem with tubular sheaths at base, 2-leaved. Leaves lanceolate, acute, 2.3–2.5 × 0.4–0.8 cm. Inflorescence terminal, racemose, 2-flowered; rachis 1 cm long; floral bract triangular. Flowers 1–1.3 cm across; sepals white, petals and lip red; pedicel and ovary twisted, 7–9 mm long. Dorsal sepal elliptic-obovate, rounded, margins glandular, 5 × 4 mm; lateral sepals elliptic, subacute, 6 × 4 mm. Petals spathulate, rounded, cucullate at apex, 5 × 2.2 mm (at wide apex). Lip 3-lobed, narrowed and subunguiculate at base, spurred, base 1 × 1 mm; lateral lobes lingulate, obtuse, 3 × 1 mm; mid-lobe lingulate, obtuse, 3 × 1 mm; spur conical, 2 mm long. Column short, 1.5–2 × 1.5 mm; pollinaria 1.6 mm long. Fruit not seen.

#### Flowering time.

August.

#### Habitat.

Terrestrial on rock crevices or cliffs at an elevation of 4100 m.

#### Distribution.

Endemic to Bhutan.

#### Note.

Our description is based on Pearce and Cribb (2001) and an image of the holotype, which is apparently the only collection made of this species so far. *Herminium albosanguineum* is similar to *H. albomarginatum*, but differs in having a fewer-flowered inflorescence, white sepals, red petals and lip. It is also similar to *H. albovirens*; see under that species for a comparison.

### 4. Herminium albovirens

Taxon classificationPlantaeAsparagalesOrchidaceae

(Renz) X.H. Jin, Schuit., Raskoti & L.Q. Huang, Cladistics 2015.

#### Basionym.

*Bhutanthera albovirens* Renz, Edinburgh J. Bot. 58: 102. 2001.

#### Type.

BHUTAN. Tongsa district, Thita Tso, Rinchen Chu, 13. 07. 1937. *Ludlow and Sherriff, 3441* (Holotype: BM! [BM000074511]).

#### Description.

Plant 4–6 cm tall. Tuber globose, 0.5–1 × 0.4–0.5 cm. Stem with 2 leaves and tubular sheaths. Leaves subopposite, lanceolate to ovate-lanceolate, subacute, 1–1.9 × 0.4–1.1 cm. Inflorescence terminal, 1–2-flowered; floral bract lanceolate, acuminate, 3 × 0.5–1mm. Flowers white, sepals and petals yellowish green, 0.8–1cm across; pedicel and ovary fusiform, 6–8 mm long. Dorsal sepal ovate, obtuse, 5 × 3.5 mm; lateral sepals obliquely ovate, falcate, subacute, 6–6.5 × 3–4 mm. Petals obovoid, rounded, 3–5 × 2.5–4 mm. Lip deeply 3-lobed, spurred, base quadrate, 0.5 × 2 mm (wide); lateral lobes oblong, rounded, 3.5–4 × 0.8mm; mid-lobe oblong, rounded, 5 × 0.6 mm; spur conical, globose, rounded, curved forwards, 2–3 × 0.8–1 mm. Column short, broad, 1 mm tall; anther-cap long-beaked, 1.5–1.9 mm long.

#### Flowering time.

July–September.

#### Habitat.

Terrestrial on alpine cliffs and in rock crevices at elevations of 4270–4720 m.

#### Distribution.

Endemic to Bhutan.

#### Specimen examined.

BHUTAN: Upper Kulong Chi district, Shingbe, 17.09.1949, *Ludlow & Sherriff, 21196* (BM! [BM000074510]).

#### Note.

Our description is based on Pearce and Cribb (2001) and an image of the holotype. *Herminium albovirens* is similar to *H. albosanguineum*, but differs in having yellowish-green sepals, yellowish-green to white petals and a white lip. More material of these species is needed to assess their variability.

### 5. Herminium biporosum

Taxon classificationPlantaeAsparagalesOrchidaceae

Maxim., Bull. Acad. Imp. Sci. Saint-Pétersbourg, sér. 3, 31: 106. 1886.

Figs 6R , 7R , 8R , 9R

Porolabium biporosum (Maxim.) Tang & F. T. Wang, Bull. Fan Mem. Inst. Biol. Bot. 10: 38. 1940.
Monorchis biporosa (Maxim.) O. Schwarz, Mitt. Thüring. Bot. Ges. 1: 95. 1949.

#### Type.

CHINA. Qinghai, near Lake Khuku-nor, 1880, *Przewalski, Spec. 1* (Holotype: LE!).

#### Description.

Plant 5–12 cm tall. Tubers globose, 6 × 4 mm. Stem slender, with 2 or 3 tubular sheaths and 1 leaf. Leaf oblong-lanceolate, 1–3.5 × 0.4–0.6 cm, apex obtuse. Inflorescence 2–10 cm, peduncle cylindrical without peduncle-scales, rachis 1–4 cm with 4–8-flowered; floral bracts scale like, ca. 1 mm. Flowers yellowish green; ovary twisted, fusiform, including pedicel 5–6 mm. Dorsal sepal forming a hood with petals, orbicular, 2.5 × 1.5 mm, apex obtuse; lateral sepals obliquely oblong-lanceolate, 3×1.5 mm, apex obtuse. Petals ovate, oblique, 2 × 1mm. Lip deflexed, 1.5–2.8 ×1.5 mm, base dilated and with 2 sharply delimited pits, attenuate toward apex. Column 0.7 mm, with 2 lateral appendages at base of anther; anther cucullate, large, 2-locular; pollinia 2, each attached to a viscidium by a short caudicle; rostellum triangular, with spreading lateral lobes; stigma cushion situated below the rostellum. Fruit fusiform, 5 mm long.

#### Flowering time.

July.

#### Habitat.

Terrestrial on grasslands slopes at elevations of 3000–3600 m.

#### Distribution.

China (Qinghai, Shanxi, Yunnan).

#### Specimen examined.

CHINA: **Yunnan**, Fugong county, 3600 m, 17.07.2013, *Jin X.H., Wang L.S.,Wang Q et al.*, ST0437 (PE).

### 6. Herminium bulleyi

Taxon classificationPlantaeAsparagalesOrchidaceae

(Rolfe) Tang & F. T. Wang, Bull. Fan Mem. Inst. Biol. 7: 130. 1936.

Peristylus bulleyi (Rolfe) K. Y. Lang, Acta Phytotax. Sin. 25: 448. 1987.
Peristylus gracillimus (Hook. f.) Kraenzl. f. lankongensis Finet, Rev. Gén. Bot. 13: 522. 1901. Type: CHINA. Yunnan, Mt. Hee-chan-men above Lan-kong, 2800 m, 3.9.1884, *Delavay J. M., 681* (Holotype P! [P00363886]).
Habenaria beesiana W.W. Sm., Notes Roy. Bot. Gard. Edinburgh 8: 189. 1914. Type: CHINA. Eastern flank of the Lichiang Range, Lat. 27° 30’ N, 10,000 ft, August 1910, *Forrest G., 6404* (Holotype E! [E00381988]; Isotype K! [K000796957]).
Platanthera praeustipetala Kraenzl., Repert. Spec. Nov. Regni Veg. 17: 103. 1921. Type: CHINA. Yunnan, Pe Tsao Lin, *Tén S., 1384* (Holotype: C).

#### Basionym.

*Habenaria bulleyi* Rolfe, Notes Roy. Bot. Gard. Edinburgh 8: 25. 1913.

#### Type.

CHINA. Yunnan, eastern slopes of the Tsan-Shan Range, west of Talifu, 7800 ft, 15.09.1905, *Forrest G., 895* (Holotype: E! [E00059069]).

#### Description.

Plant slender, 15–30 cm tall. Tubers oblong, 1–3.5 × ca. 0.5–1 cm. Stem with 2 tubular sheaths, 2–4 leaved. The two lowest leaves subopposite, others more distant, linear, 4–10× 0.2–0.6 cm, apex acute or acuminate. Inflorescence 15–25cm; peduncle cylindrical, slender; peduncle-scales1–3, linear-lanceolate, 5–7 mm; rachis 5–15 cm, laxly many-flowered; floral bracts ovate-lanceolate, 4–6 mm, shorter than ovary, apex acuminate. Flowers yellowish green, secund; pedicel and ovary 5–10 mm, apex beaked. Dorsal sepal ovate-oblong, concave, 1.5–3.5 × 0.6–1.2 mm, apex obtuse; lateral sepals oblong, oblique, 3–4 × 0.8–1.5 mm, apex obtuse. Petals ovate-lanceolate, ca. 3.5 × 1.5 mm, fleshy above middle, apex acute. Lip narrowly oblong, 3–4.5 × ca. 0.4 mm, 3-lobed near middle; lateral lobes oblong, 0.6–2.5 mm; mid-lobe linear-oblong, 2–3 mm, wider than lateral lobes, apex obtuse; spur pendulous, curved forward, cylindrical, 4–6 mm, equal or longer than ovary, apex dilated, obtuse. Column ca. 1 mm; viscidia ellipsoid, rostellum triangular, stigma ca. 1 mm, drawn out at the base of the lip.

#### Flowering time.

July–September.

#### Habitat.

Terrestrial in forest margins, in dry pastures in open pine forest, or on grassy slopes at elevations of 2500–3500 m.

#### Distribution.

China (Sichuan, Yunnan).

#### Specimens examined.

CHINA: **Sichuan**, Daocheng county, Riwagongshe, Xiayong, 3300 m, 24.08.1981, *Hengduan Mountain Team, 4193* (PE); Daocheng, 3500 m, 18.08.1973, *Sichuan Botanical Survey Team, 2534* (PE); Muli, 03.08.1937, *Yu T. T., 7560* (PE); **Yunnan**, Dali (‘Tali’), 3000 m, 08.1914, *Schneider C., 3100* (K); Heqing county, 2500 m, 18.08.1929, *Chang Q., 23708* (PE); Lichiang Range, 3050 m, 08.1922, *Forrest G., 22016* (K); Shangri-La, 3200 m, 15.07.2001, *HK Kadoorie Team, 1114* (PE); Shangri-La, 3250 m, 09.08.1981, *Tang Z. X., Tian X.W., Zhang Z.H., 842* (PE); Xianggelila (Shangri-La), 3300 m, 13.08.1962, *Zhongdian Team, 734* (PE); Shangri-La, Xiaozhongdian farm, Dongshan, 3250, 13.03.1981, *Beijing Hengduan Mountain Team, 3025* (PE); Zhongdian County, Mt. Haba Shan, 07.2002, 2700 m, *Sun Hang, 05* (K).

#### Notes.

*Herminium bulleyi* is similar to *H. mannii* but the spur of *H. bulleyi* is much longer and curved whereas *H. mannii* has a scrotiform spur.

### 7. Herminium chloranthum

Taxon classificationPlantaeAsparagalesOrchidaceae

Tang & F. T. Wang, Bull. Fan Mem. Inst. Biol. Bot. 10: 34. 1940.

Figs 6B , 7B , 8B , 9B

#### Type.

CHINA. Yunnan, Shangri-La (Zhongdian County), 3000 m, 05.07.1937, *Yu T. T., 11920* (Holotype: KUN!; Isotype: AMES! [AMES00104951], PE! [PE01432254]).

#### Description.

Plant 4–16 cm tall. Tubers globose, 4–15 × 4–10 mm. Stem with 2 or 3 tubular sheaths and 1–2 leaves at base. Leaves subopposite or opposite, oblong, elliptic, or spatulate, 2–7 × 0.3–2 cm, apex obtuse or acute. Inflorescence 3–14 cm; peduncle cylindrical, ebracteate; rachis 1–5 cm, laxly flowered; floral bracts ovate, shorter than ovary, apex obtuse. Flowers pale green; 5 mm across, pedicel and ovary twisted and hooked, 3–6 mm. Dorsal sepal broadly ovate, concave, 3–4 × 2–2.5 mm, apex obtuse; lateral sepals obliquely lanceolate, 3–4 × 1–1.4 mm, apex obtuse. Petals elliptic to lancolate, falcate, 3–4 × 1.3–1.8 mm, upper half-fleshy, apex obtuse. Lip pandurate, 5.5 × 2.5 mm, concave with spur like saccate base, 3-lobed above middle; lateral lobes triangular, 0.5 × 0.8 mm, apex obtuse; midlobe triangular, 1 × 0.6 mm, apex obtuse. Column 0.7 mm; pollinia globose; caudicles nearly obsolete, viscidia involute, hornlike, rostellum triangular, armed, stigma transversely oblong, situated below the rostellum. Fruit oblong, 6–7 mm long.

#### Flowering time.

July–August.

#### Habitat.

Terrestrial in alpine grasslands and meadows at elevations of 2500–4100 m.

#### Distribution.

China, Nepal.

#### Specimens examined.

CHINA: **Sichuan**, Kangding county, near Suopo, 3600 m, 03.08.1982, *Lang K. Y., Li L. Q., Fei Y., 948A* (PE); **Tibet**, Medog county, Bomi-Medog Road, 08.09.2009, *Jin X. H., Liu B. Q., Xu S. M., Zhao W., 2664* (PE); Nyalam county, 3800 m, 02.09.1972, *Tibetan Herbal Survey Team,1909* (PE); Zayu county, Chawalong from Meigu to Ridong, 3700 m, 17.07.2010, *Jin X. H.*, *Liu B. Q.*, *Xu S. M.*, *Zhao W.*, *STET0611* (PE); **Yunnan**, Li-Kiang Hsien, 07.1935, *Wang C. W.,71033* (PE), Shangri-La, 3000 m, 05.07.1937, *Yu T. T., 19920* (PE); Shangri-La, 4300 m, 18.08.1981, *Beijing Hengduan Mountain Team, 3319* (PE).

NEPAL: Dhaulagiri, Mustang district, above Jharkot at 3800 m, 18.08.2011, *Raskoti B. B.,00139* (KATH); Mustang, Bhena, 3780 m, 26.08.2001, *Miehe G. and Miehe S., 01-072-16* (K).

### 8. Herminium clavigerum

Taxon classificationPlantaeAsparagalesOrchidaceae

(Lindl.) X.H. Jin, Schuit., Raskoti & L.Q. Huang, Cladistics 2015.

Figs 6N , 7N

Habenaria densa Wall. ex Lindl., Gen. Sp. Orchid. Pl. 326. 1835. Type: Nepal, *Wallich N., 7046* (Holotype: K-WALLICH!).
Habenaria clavigera (Lindl.) Dandy, J. Bot. 68: 246. 1930.
Platantheroides densa (Wall. ex Lindl.) Szlach., Richardiana 4: 106. 2004.
Habenella clavigera (Lindl.) Szlach. & Kras-Lap., Richardiana 6: 34. 2006.

#### Basionym.

*Platanthera clavigera* Lindl., Gen. Sp. Orchid. Pl. 289. 1835.

#### Type.

INDIA, Himachal Pradesh, Simla, 08.1831, *Dalhousie C., s.n.* (Holotype: K-LINDL; Isotype K! [K000247390]).

#### Description.

Plant 12–70 cm tall. Tubers, ovoid, 1–2 × 0.6–1 cm. Stem with 2 or 3 tubular sheaths at base and 4–5 spaced leaves above middle. Leaves ovate to elliptic, 4–10 × 1–4 cm, apex acuminate. Inflorescence 20–36 cm, peduncle cylindrical with 1 foliaceous bract; rachis 5–28 cm, densely many flowered; floral bracts longer than ovary, lanceolate, 5–20 cm, apex acuminate. Flowers yellowish green; pedicel and ovary cylindrical-fusiform, apex beaked, 6–12 mm. Dorsal sepal broadly ovat-elliptic, 3–5 × 2–2.5 mm, margin ciliate, apex obtuse; lateral sepals obliquely elliptic-oblong, 3–5.5 × 1.5–2.5 mm, margin ciliate, apex obtuse. Petals obliquely oblong-ovate, 3–5 × 2–2.5 mm, apex acute. Lip lingulate, 4–5.5 × 1–1.5 mm, entire, apex obtuse; disk with a conic callus in front of mouth of spur; spur pendulous, clavate, 5–6 mm, much shorter than ovary. Column 5 mm; staminodes small, elliptic; anther locules nearly parallel; pollinia subglobose, with very short caudicles, viscidia orbicular; rostellum erect, triangular; stigma lobes separate, raised, shortly clavate to narrowly oblong, spreading on either side of base of lip.

#### Flowering time.

August–September.

#### Habitat.

Terrestrial in forest margins and on grassy slopes at elevations of 2300–3400 m.

#### Distribution.

Bhutan, China, India, Nepal.

#### Specimens examined.

CHINA: **Tibet**, Cona county, 3200 m, 09.09.1975, *Qinghai-Tibet Team, 751858* (PE); Cona county, Mama town, 3040 m, 06.08.1974, *Qinghai-Tibet Team, 2257* (PE); Cona county, Le town, 2430 m, 08.08.1974, *Qinghai-TibetTeam, 2477* (PE); Cona county, Le bu courtyard, 2789 m, 07.09.2012, *FLPH Tibet Expedition, 12–0655* (PE); Gyirong county, 2895 m, 07.08.2010, *PE-Tibet Team, 00304* (PE); Gyirong county, 3200 m, 19.07.1975, *Qinghai-Tibet Team, 4713* (PE); Gyirong county, 3050 m, 24.07.1975, *Qinghai-Tibet Team,4789* (PE); Nyalam county, 2800 m, 14.08.1972, *Tibetan Herb Survey Team, 1191* (PE), Yadong county, 3012 m, 28.08.2010, *PE-Tibet Team, 02178* (PE); Yadong county, 2700 m, 11.08.1974, *Qinghai-Tibet Team, 74–2255* (PE).

INDIA: **Sikkim**, Donang, 3050 m, 10.08.1943, *Pradhan J., s.n.* (K); **Uttarakhand**, Kumaon, 17.09.1900, *Inayat, 24066* (K).

NEPAL: **Bagmati**, Rasuwa District, Dhunche, 1820 m, 27.08.1977, *Saiju H. K., and Amatya P. M., 1777* (KATH); **Janakpur**, Dolakha District, 2400 m, 31.08.1983, *Rajbhandari K. R., 9542* (KATH); **Koshi**, Dhankuta District, Chitre, 2000 m, 19.09.1983, *Saiju H. K., Joshi L., Pradhan N., and Subedi M. N.,1* (KATH); **Mahakali**, Darchula District, Thin, 2300 m, 27.08.1980, *Rajbhanari K. R. and Malla K. J. 5635* (KATH); **Mechi**, Ilam District, Hile, 2420 m, 06.10.1977, *Pradhan P., Rajbhandari K. R. and Niraula R. 265* (KATH).

### 9. Herminium coeloceras

Taxon classificationPlantaeAsparagalesOrchidaceae

(Finet) Schltr., Notes Roy. Bot. Gard. Edinburgh 5: 97. 1912.

Figs 6I , 7I , 8I , 9I , 11I

Herminium unicorne Kraenzl., Repert. Spec. Nov. Regni Veg. 5: 199. 1908. Type: CHINA. East Tibet, Tongolo, *Soulié J. A., 2980* (Holotype: B, lost; Isotype: P! [P00361310]).
Herminium tenianum Kraenzl., Repert. Spec. Nov. Regni Veg. 17: 110. 1921. Type: CHINA. Yunnan, Pe Tsao Lin, *Tén S., 1387* (Holotype: C).
Monorchis coeloceras O. Schwarz, Mitt. Thüring. Bot. Ges. 1: 96. 1949.
Monorchis teniana (Kraenzl.) O. Schwarz, Mitt. Thüring. Bot. Ges. 1: 96. 1949.

#### Basionym.

*Peristylus coeloceras* Finet, Rev. Gén. Bot. 13: 519. 1902.

#### Type.

CHINA. Yunnan, Mt. Hee chan menn above Lan Kong, 11.07.1883, *Delavay J. M.,68* (Lectotype, here chosen: P! [P00361306]). Syntypes: Yunnan, Mt. Hee chan menn above Lan Kong, 11.07.1883, *Delavay J. M., s.n.* (Syntype: P! [P00361307]); Yunnan, summit of Ma eul chan, 3500 m, 9.7.1889, *Delavay J. M., s.n.* (Syntype: P! [P00361303 & P00361304]); Yunnan, forest of Song pin above Ta pin tze, 2000 m, 18.8.1885, *Delavay J. M., s.n.* (Syntype: P! [P00361308 & P00361309]); Sichuan, Tongolo, Ragathong, August 1891, *Soulié J. A., 322* (Syntype: P! [P00361305]).

#### Description.

Plant 6–36 cm tall. Tubers oblong-ovoid, 10–25 × 5–10 mm. Stem with 1 or 2 tubular sheaths and 2 opposite to subopposite leaves at base. Leaves elliptic-lanceolate to oblong, 2–10 × 0.8–2 cm, apex obtuse or acute. Inflorescence 20–27 cm; peduncle cylindrical, slender; peduncle-scales1–3, lanceolate, 4–15 mm; rachis 2–10 cm, densely many flowered; floral bracts ovate-lanceolate, 5–10 mm, equal or slightly longer than ovary, apex acuminate. Flowers white, ca. 4 mm across, pedicel and ovary twisted, arcuate, beaked, 4–10 mm. Dorsal sepal ovate, 2–2.5 × 1.5–2 mm, apex obtuse; lateral sepals ovate, 2.5–3 × 1–1.5 mm, apex obtuse. Petals nearly rhombic to ovate, oblique, 2–2.5 × 1–1.5 mm, apex obtuse. Lipspurred, nearly ovate, 2–3 × 1.5–2 mm, 3-lobed; disk with subglobose callus; lateral lobes oblong,1 × 0.5 mm, apex obtuse; mid-lobe oblong, 2 × 1 mm, apex obtuse; spur globose-saccate. Column ca. 2 mm; pollinia globose, viscidia involute, hornlike, elliptic; rostellum with short arms, stigma transversely oblong, situated below the rostellum.

#### Flowering time.

June–August.

#### Habitat.

Terrestrial in coniferous and broad-leaved mixed forests, and on grassy alpine slopes at elevations of 1800–3900 m.

#### Distribution.

China, Myanmar.

#### Specimens examined.

CHINA: **Sichuan**, Luding county, 1300 m, 12.07.1982, *Lang K.Y., Li L.Q., Fei Y.Y., 624* (PE); Luding county, 1800 m, 20.08.1963, *Guan K.J.*, *Wang W.C.*, *et al. 1762* (PE); **Tibet**, Zayu county, 3400 m, 13.08.2013, *Jin X. H.*, *Wang L. S.*, *Wang Q et al.*, *ST2901* (PE); Gonjo county, 3700, 20.08.1976, *Qinghai-Tibet Team*, *9681* (PE); Mainling county, 3400 m, 20.07.1972, *Tibet Herb Survey Team, 4111* (PE); Nyingchi county, 3100 m, 31.07.1965, *Zhang Y.T. and Lang K.Y., 1213* (PE); Pasang Lake, 3050 m, 18.08.1924, *Kingdon-Ward F., 6107* (K); **Yunnan**, Shangri-La, 2830 m, 29.07.1997, *Luo Y.B., 176* (9752) (PE); Lijiang county, 2500 m, 03.10.1983, *Qinghai-Tibet Team, 14921* (PE).

### 10. Herminium coiloglossum

Taxon classificationPlantaeAsparagalesOrchidaceae

Schltr., Repert. Spec. Nov. Regni Veg. 3: 15. 1906.

Figs 8H , 9Q

Monorchis coiloglossa (Schltr.) O. Schwarz, Mitt. Thüring. Bot. Ges. 1: 95. 1949.

#### Type.

CHINA. Yunnan, Szemeo, *Henry A., 13556* (Holotype: B, lost; Isotype: K! [000079037], NY! [NY00008941]).

#### Description.

Plant 7–31 cm tall. Tubers ovoid-oblong, 4–20 × 3–5 mm. Stem with 2 tubular sheaths and 1 leaf at base. Leaf linear-oblong, 4–12 × 0.3–0.8 cm, apex acute. Inflorescence 6–27 cm; peduncle cylindrical, with 2–7 lanceolate peduncle-scales; rachis 1.5–11 cm, laxly many flowered; floral bracts ovate, 1–3 mm, shorter than ovary, apex acuminate. Flowers yellowish green; pedicel and ovary twisted, straight, 4–6 mm. Dorsal sepal orbicular-ovate, concave, 1.2–1.5 × 1–1.2 mm, apex obtuse; lateral sepals ovate, slightly oblique, 1.5–2 × 1–1.2 mm, apex obtuse. Petals hooded with dorsal sepal, oblong, oblique, 1.2 × 0.7 mm, apex obtuse. Lip oblong to nearly pandurate, 1.5–2 × 0.8–1 mm, contracted at middle, base dilated and concave, apex 3-lobed; lateral lobes triangular-falcate, ca. 0.2 × 0.08 mm, apex subacute; mid-lobe broadly triangular, 0.3 × 0.3 mm, apex obtuse. Column ca. 1 mm; pollinia globose; caudicles short, viscidia ovoid, rostellum triangular, stigma transversely oblong situated below the rostellum. Fruit oblong, 4–5 mm long.

#### Flowering time.

August–September.

#### Habitat.

Terrestrial on grassy slopes at elevations of 1600–2800 m.

#### Distribution.

China (Yunnan).

#### Specimens examined.

CHINA: **Yunnan**, Jingdong county, 1600 m, 11.10.1939, *Li M. K.*, 0449 (PE); Lanping Camp, way to Yakou, 2700 m, 11.06.1981, *Hengduan Mountain Expedition Team, 3925* (PE).

### 11. Herminium ecalcaratum

Taxon classificationPlantaeAsparagalesOrchidaceae

(Finet) Schltr., Repert. Spec. Nov. Regni Veg. Beih.4: 101. 1919.

Fig. 8Q

Monorchis ecalcarata (Finet) O. Schwarz, Mitt. Thüring. Bot. Ges. 1: 95. 1949.

#### Basionym.

*Peristylus ecalcaratus* Finet, Rev. Gén. Bot. 13: 520. 1901.

#### Type.

CHINA. Yunnan, Mt. Heechan men (Lan Kong), 3000 m, 11.09.1885, *Delavay J.M., 1692* (Lectotype, here chosen: P! [P00363792]); Syntype: Same locality, 2800 m, 03.09.1884, *Delavay J.M., 682* (P! [P00363791]).

#### Description.

Plant 8–20 cm tall. Tubers oblong-lanceolate, 4–10 × 3–5 mm. Stem with 2 tubular sheaths at base, 2-leaved. Leaves alternate, oblong, 10–12 × 0.3–1 cm, apex obtuse. Inflorescence 10–16 cm; peduncle cylindrical, with 1–4 ovate-lanceolate 2–3 mm peduncle-scales; rachis 3–7 cm, densely many-flowered; floral bracts ovate-lanceolate, 2–3 mm, shorter than ovary, apex acute. Flowers white; pedicel and ovary arcuate, twisted, slightly beaked near apex, 2–4 mm. Dorsal sepal ovate, concave, 1.5–2.2 × 0.8–1.3 mm, apex obtuse; lateral sepals ovate, oblique, 1.5–2 × 0.7–1 mm, apex obtuse. Petals connivent with dorsal sepal, ovate, oblique, 1–1.5 × 0.8–1 mm, apex acute. Lip obovate, 1.6–2.3 × 1–1.4 mm, base concave, apex 3-lobed; lateral lobes triangular, 0.3 × 0.4 mm, apex obtuse; mid-lobe broadly ovate, 1 × 1 mm, apex obtusee. Column ca. 0.9 mm; pollinia globose; caudicles short, viscidia elliptic, rostellum triangular with two distinct lobes, stigma transversely oblong situated below the rostellum.

#### Flowering time.

September.

#### Habitat.

Terrestrial on alpine grassy slopes and meadows at elevations of 2500–3900 m.

#### Distribution.

China (Sichuan, Yunnan).

#### Specimen examined.

CHINA: **Sichuan**, Hongyuan county, north of Partridge Hill, 3850 m, 02.07.1983, (without collector), *1284* (PE); **Yunnan**, Fugong county, Shangpa Town, Zhuminglin courtyard, 3020 m, 27.08.2005, *Jin X.H.,7913* (PE); Heqing county, Fragrant river, 2600 m, 03.09.1929, *Ching R.C., 24318* (PE); Lijiang county, east of Yulong mountain, 2900 m, 05.07.1981, *Beijing Hengduan Mountain Team*, 02698 B (PE).

### 12. Herminium edgeworthii

Taxon classificationPlantaeAsparagalesOrchidaceae

(Hook.f. ex Collett) X.H. Jin, Schuit., Raskoti & L.Q. Huang, Cladistics 2015.

Fig. 5F

Platanthera edgeworthii (Hook.f. ex Collett) R.K. Gupta, Fl. Nainital 349. 1968.
Platantheroides edgeworthii (Hook.f. ex Collett) Szlach., Richardiana 4: 106. 2004.

#### Basionym.

*Habenaria edgeworthii* Hook.f. ex Collett, Fl. Siml. 504. 1902.

#### Type.

INDIA. Himachal Pradesh, Simla, Banatar, 08.1834, *Edgeworth s.n.* (Holotype: K! [K000247384]).

#### Description.

Plant 20–50 cm tall. Tuber subglobose to ellipsoid, 1–2.4 × 0.4–9 cm. Stem with tubular sheaths at base, 3–5-leaved. Leaves well-spaced, broadly ovate to ovate-oblong, lanceolate, 4–8 × 1–4 cm, apex acute. Inflorescence 20–30 cm, peduncle cylindrical with foliaceous lanceolate bracts, apex acute, 1–3 × 2–4 cm, rachis 7–28 cm long with densely many flowers; floral bracts lanceolate, acute, equal or shorter than ovary, 5–15 × 2–3 mm. Flowers pale yellowish green to greenish yellow, ca. 1 cm long, pedicel and ovary arcuate, beaked, twisted, 5–15 cm long. Dorsal sepal broadly elliptic-ovate, concave, margin ciliated, 3–4 × 4–5 mm, lateral sepals reflexed, oblong ovate, margin ciliated, apex obtuse, 4.5–6 × 2–3 mm. Petals obliquely ovate-lanceolate, apex acute, 4–6 × 1.5–2 mm. Lip entire, lingulate, 7 × 2 mm with concave at base, slightly fleshy, clawed above base, apex obtuse, spur cylindrical, spreading upwards, longer than the ovaray, 1–2 cm long. Column 4 mm long, pollinia subglobose, with short caudicle, viscidia orbicular; rostellum triangular, ca. 2 mm; stigma lobes separate, clavate to oblong, ca. 2 mm, spreading on either side of lip base.

#### Flowering time.

August–September.

#### Habitat.

Terrestrial in *Quercus* forest and forest margins, and in subalpine grassland at elevations of 1800–2500 m.

#### Distribution.

China, India, Myanmar, Nepal.

#### Specimens examined.

CHINA: **Tibet**, Yatung, 1897, *Hobson H. E., s.n.* (K); Jatung, 3050 m, 03.08.1936, *Spencer Chapman F., 334* (K); Lhasa, 1947, *Guthrie J., s.n.* (K).

INDIA: **Uttarakhand**, *Almora, S. R.* and *Winterbottom J. E., 41* (K).

NEPAL: **Bagamati**, Rasuwa District, Ghatte Khola, 1900 m, 21.08.1964, *Shrestha T. B.,and Bista M. S., 1847* (KATH); **Gandaki**, Manang District, Dhanagyung-Dharapani, 2000 m, 07.08.1983, *Rajbhandari K. R., 9061* (KATH); **Karnali**, Kalikot District, near Kadampur, 1800 m, 31.08.1985, *Shakya P.R., Subedi N. N., and Uprety R., 8486* (KATH); **Mahakali**, Darchula District, Kasoti, 2430 m, 20.08.1984, *Shakya P. R., Adhikari M. K., and Subedi N. N., 7968* (KATH).

### 13. Herminium elisabethae

Taxon classificationPlantaeAsparagalesOrchidaceae

(Duthie) Tang & F.T. Wang, Bull. Fan Mem. Inst. Biol. 7: 129. 1936.

Figs 6L , 7L , 8L , 9L

Peristylus elisabethae (Duthie) R. K. Gupta, Fl. Nainital 351. 1968.

#### Basionym.

*Habenaria elisabethae* Duthie, J. Asiat. Soc. Bengal, Pt. 2, Nat. Hist. 71: 44. 1902.

#### Type.

INDIA. Uttaranchal, Tehri-Garhwal, Nag Tiba, 08.1899, 2743–3948 m, *Mackinnon P. W., 22990* [*a “Duthie 22990*”] (Lectotype, here chosen: K! [K000387522]; Isolectotype: K! [K000387586]; P! [P00426409]). Syntypes: INDIA. Simla, *Edgeworth*, *Lady E. Bubington Smith s.n.* (Syntype: CAL); Naini Tal, up to 8000 ft, *Davidson s.n.* (Syntype: CAL); Tehri-Garhwal, 7000-10000 ft, *Duthie 524* (Syntype: CAL); near Mussoorie, 6000–7000 ft, *Mckinnon P. W.*, 22990 (Syntype: K! [K000387523]).

#### Description.

Plant 10–40 cm tall. Tubers oblong-ellipsoid, 1–2.5 × 0.5–1 cm. Stem with 1 or 2 tubular sheaths at base, 2–3-leaved. Leaves cauline, elliptic to lanceolate, 3–12 × 0.8–2 cm, apex acute or acuminate. Inflorescence 6–32 cm; peduncle cylindrical, slender; peduncle-scales 1 or 2, lanceolate, to 3 cm; rachis 3–22 cm, rather laxly to subdensely many-flowered; floral bracts lanceolate, 4–8 mm, nearly equal to or shorter than ovary, apex acuminate. Flowers subsecund, yellowish green; pedicel and ovary arcuate, shortly beaked, 6–9 mm. Dorsal sepal broadly ovate, concave, 1.5–2 × 1.5–2 mm, apex obtuse; lateral sepals oblique, ovate, 1.5–2.3 × 1–1.5 mm, apex acute. Petals hooded with dorsal sepals, ovate, oblique, upper half fleshy, 2–3 × 1–1.5 mm, apex obtuse. Lip oblong, 2–3 × 1–1.5 mm, base concave, 3-lobed near middle; lateral lobes oblong, 1 × 0.5 mm, apex obtuse; mid-lobe oblong, 1.5 × 1.3 mm, apex obtuse; spur pendulous, oblong-clavate, 1 mm, apex obtuse. Column 1 mm; pollinia ellipsoid, viscidia involute, rostellum triangular, stigma transversely oblong situated below the rostellum. Fruit oblong, 0.5–1 cm long.

#### Flowering time.

July–September.

#### Habitat.

Terrestrial in coniferous and broad-leaved mixed forests and on alpine grassy slopes at elevations of 2100–4100 m, once reported growing on oak trees.

#### Distribution.

Bhutan, China, India, Myanmar, Nepal.

#### Specimens examined.

BHUTAN: Gasa Dist., Upper Mo Chu, around Taktsimakhang, 3560–3600 m, 14.08.2000, *Miehe G. and Miehe S., 00-286-03* (K); Gasa Dist., Upper Mo Chu, 3900 m, 12.08.2000, *Miehe G. and Miehe S., 00-278-04* (K).

CHINA: **Tibet**, Cona county, 3300 m, 10.09.1974, *Qinghai-Tibet Team, 75–1924* (PE); Gyirong county, 3200 m, 24.07.1975, *Qinghai-Tibet Team, 7071* (PE); Lhasa Dist., 0.5–0.7.1947, *Guthrie J., s.n.* (K); Luozha county, 2764 m, 17.08.2013, *Chen*, *Y. S.*, *Chang*, *Z.Y.*, *Deng M.*, *et al., 13–1461* (PE); Nyingchi county, 3100 m, 04.08.1965, *Zhang Y.T., and Lang K.Y., 1319* (PE); Yadong county, 4100 m, 11.09.1974, *Qinghai-Tibet Team, 74–2304* (PE); Yadong county, 3600 m, 25.08.2010, *PE-Tibet Team, 01784* (PE); Yadong county, 3900 m, 27.08.2010, *PE-Tibet Team, 02069* (PE); Zayu, junction of the Di Chu and Zayul Chu, “5000–7000 ft” (1500–2100 m), 16.07.1926, *Kingdon-Ward F., 7145* (K); east of Jatung, 3050 m, 03.08.1936, *Spencer Chapman F., 349* (K).

INDIA: **Himachal Pradesh**, Kulu-Lahaul, 1888, *Drummond J. R., 23/94* (K); Uttarakhand, Kumaon, 3650 m, undated, *Strachey R. and Winterbottom J. E., 32* (K); **Uttarakhand**, Mussoorie, Nag Sika, 05.08.1898, *Mackinnon P., 21766* (K); East of Tehri, 09.1901, *Mackinnon P., 25412* (K).

NEPAL: **Gandaki**, Manang District, Hunde, 3350 m, 04.08.1983, *Rajbhandari K. R., 8824* (KATH); **Janakpur**, Dolakha District, Sekpa to Gyangsar, 2800 m, 20.07.1977, *Rajbhandari K. R.*, and *Roy B., 1641* (KATH), **Koshi**, Sankhuwasabha District, Guphapokhari, 2900 m, 30.08.1989, *Grey-Wilson C., Zmarzty S., Sinnott M., Long D., Mc Beath R., Noltie H. and Subedi M., 99* (K, KATH); Jaljale Himal, Gu-Padara ridge, 2780 m, 23.08.1984, *Farille M. and Lachard G., 847536* (K); above Tukele, 3000 m, 24.07.1973, *Grey-Wilson and Phillips, 361* (K); Langtang, 2900 m, 07.09.1971, *Dobremez J. F., 1011* (K); Tinjure Danda, 2750 m, 07.09.1867, *Williams and Stainton, 8409* (K).

### 14. Herminium fallax

Taxon classificationPlantaeAsparagalesOrchidaceae

(Lindl.) Hook. f., Fl. Brit. India 6:129. 1890.

Figs 5D , 6J , 7J , 8J , 9J , 10I , 11F

Habenaria fallax (Lindl.) King & Pantl., Ann. Roy. Bot. Gard. (Calcutta) 8: 325. 1898.
Platanthera fallax (Lindl.) Schltr., Repert. Spec. Nov. Regni Veg. Beih.4: 111. 1919.
Monorchis fallax (Lindl.) O. Schwarz, Mitt. Thüring. Bot. Ges. 1: 95. 1949.
Peristylus fallax var. dwarikae Deva & H.B. Naithani. Orchid Fl. N.W. Himalaya 187. 1986. Type: INDIA. Uttarakhand, Dodital, Uttarkashi, alt. 9200 ft, 8.1974, *Dwarika Prasad s.n.* (Holotype: DD).

#### Basionym.

*Peristylus fallax* Lindl., Gen. Sp. Orchid. Pl. 298. 1835.

#### Type.

NEPAL. Without precise locality, 1821, *Wallich, 7412* (Holotype: K-LINDL!; Isotype: K-WALLICH! [K000974164]).

#### Description.

Plant 14–30 cm tall. Tubers subglobose, 1–1.5 × ca. 0.8 cm. Stem with 1 basal leaf and 2 or 3 tubular sheaths at base. Leaf oblong-elliptic to oblanceolate, 6–13 × 0.8–2 cm, apex acute. Inflorescence 11–22 cm; peduncle cylindrical, with or without 1 lanceolate peduncle scale to 20 mm; rachis 5–14 cm, densely many-flowered; floral bracts lanceolate, 6–15 × 2.5 mm, basal one longer than flowers, apex acuminate. Flowers yellowish green; pedicel and ovary twisted, straight, apex shortly beaked, 6–7 mm. Dorsal sepal ovate, concave, 2–4 × 1.5–2.1 mm, apex obtuse; lateral sepals obliquely ovate, concave, 3–4.2 × 1–1.5 mm, apex subacute. Petals obliquely ovate-lanceolate, 2.5–5 × 1–1.5 mm, apex acute. Lip oblong, 3–5.5 × 1–2 mm, ecallose, 3-lobed near middle; lateral lobes oblong, 1.5 × 0.5 mm, apex obtuse, mid-lobe oblong, 1–2 × 2 mm, apex obtuse, spur oblong-clavate, 0.7–1.5 mm, apex obtuse, shallowly 2-lobed. Column 1.5–2 mm; pollinia globose, caudicles short, viscidia disklike; rostellum triangular, broad, with arms, stigma transversely oblong, situated below the rostellum. Fruit oblong, 1 cm long.

#### Flowering time.

July–September.

#### Habitat.

Terrestrial in forest margins and on roadside slopes at elevations of 3000–4000 m.

#### Distribution.

China, Bhutan, India, Nepal.

#### Specimens examined.

BHUTAN: Gasa District, W of Thanza, 3800–4000 m, 04.09.2000, *Miehe G. and Miehe S., 00-351-01* (K).

CHINA: **Sichuan**, Yanyuan county, 3600 m, 07.08.1983, *Qinghai-Tibet Team, 12709* (PE); **Tibet**, Cona county, 3300 m, 10.09.1975, *Qinghai-Tibet Team, 751924* (PE); Gyilong county, 3150 m, 19.07.1975, *Qinghai-Tibet Team, 7017* (PE), Yadong county, Xiasima, 3600–3900 m, 02.08.2012, *Jin X. H., Jin W.T., Xu S. 13226* (PE); **Yunnan**, Dali City, Xi zhou town, 3000 m, 17.07.2009, *Yunnan Expedition, YN-ET 1652* (PE); without data, *Forrest G., 6837* (K).

INDIA: **Sikkim**, Lachen, *Hooker J. D., 288* (K); **Utarrakhand**, Kumaon, Munshiyari, 05.08.1900, *Inayat, 24058* (K).

NEPAL: **Koshi**, Terhthum District, Tinjure, 3060 m, 06.07.1972, *Shakya P. R., 1990* (KATH); **Rapti**, Dailekh District, Lamabagar to Hum, 16.07.1977, *Rajbhandari K. R., and Roy B., 1450* (KATH); **Mechi**, Ilam District, Khapore, 3033 m, 10.07.1977, *Pradhan P., Rajbhandari K. R., and Niraula R., 287* (KATH).

### 15. Herminium fimbriatum

Taxon classificationPlantaeAsparagalesOrchidaceae

(Raskoti) X.H. Jin, Schuit., Raskoti & L.Q. Huang, Cladistics 2015.

#### Basionym.

*Bhutanthera fimbriata* Raskoti, Phytotaxa 62: 57–60. 2012

#### Type.

NEPAL. Rasuwa, 3800 m, 14 July 2008, *Raskoti B. B., 234* (Holotype: KATH!).

#### Description.

Plant 11 cm tall. Tubers subglobose, 5 mm in diameter. Stem with an ovate, tubular sheath and 2 leaves at base. Leaves opposite, elliptic-ovate, 3.0–3.5 × 2.0–2.2 cm, apex obtuse. Inflorescence densely up to 8-flowered; peduncle 7 cm long, rachis 2.5 cm long, lower floral bracts ovate-triangular, 1 × 1 mm, apex acute, upper bracts lanceolate, 2 × 1 mm, apex acuminate. Flowers 1 cm across, sepals green with white margins, petals and lip white; pedicel and ovary fusiform, ridged, 7 mm long, apex beaked. Dorsal sepal broadly ovate, 4.0–5.0 × 2.5 mm, margin of the apical portion weakly fimbriate, apex obtuse; lateral sepals elliptic-ovate, oblique, 5.0–6.0 × 2.0–2.5 mm, margin of the apical portion weakly fimbriate, apex obtuse. Petals orbicular-elliptic, concave, 2.5 x 2.5 mm, apex rotund. Lip oblong, 4.0 × 1.5 mm, fleshy, trilobed below the middle; side lobes oblong-lanceolate, 2.0 × 0.5 mm, apex acute; midlobe oblong, 2.5 × 1.0 mm, apex obtuse; spur cylindrical with slightly dilated apex, 3.0–4.0 mm long, apex obtuse. Column 1 mm long, pollinia clavate-elliptic. Fruit oblong.

#### Flowering time.

July

#### Habitat.

Terrestrial in subalpine meadows and open moist places at elevation of 3800 m.

#### Distribution.

Endemic to Nepal.

### 16. Herminium forceps

Taxon classificationPlantaeAsparagalesOrchidaceae

(Finet) Schltr., Notes Roy. Bot. Gard. Edinburgh 5: 97. 1912.

Fig. 5C , 11G

Habenaria forceps (Finet) Schltr., Repert. Spec. Nov. Regni Veg. Beih.4: 127. 1919.
Herminium tsoongii Tang & F. T. Wang, Contr. Inst. Bot. Natl. Acad. Peiping 2: 134. 1934. Type: CHINA. Yunnan, without precise locality, 06.06.1919, *Tsoong K. K.*, 4916 (Holotype: N).
Herminium liguliforme Tang & F. T. Wang, Acta Phytotax. Sin.1: 61. 1951. Type: CHINA. Sikang, *Wang 66089* (Holotype: PE).

#### Basionym.

*Peristylus forceps* Finet, Rev. Gén. Bot. 13: 521. 1902.

#### Type.

CHINA. Yunnan, in the mountains, 07.1897, *Ducloux F., 319* (Lectotype, here chosen: P! [P00363813]; Isolectotype: P! [P00363812]). Syntypes: Kouy-Tcheou, Gan-pin area, 16.8.1897, *Bodinier E. & Martin L., 1779* (Syntype: P! [P00363805]); Teou chan, 15.7.1899, *Cavalerie J*., 1779 (Syntype: P! [P00363806]); Above Ta pin tze, 11.8.1882, *Delavay J. M., 381* (Syntype: P! [P00363808]); Mt. Hee Chan men (Lan Kong), 9.1884, *Delavay J. M*., 681 p.p. (Syntype: P! [P00363809]); Mt. Che tcho tze, above Ta pin tze, 8.8.1887, *Delavay J. M. s.n.* (Syntype: P! [P00363810 & P00363811]); Kouy-Yang area, 16.6.1898, *Bodinier E., 2206* (Syntype: P! [P00363814]).

#### Description.

Plant 12–40 cm tall. Tubers ovoid-oblongoid, 1–4 cm long. Stem slender, with 2 or 3 tubular sheaths and (2–)3–4(–5) widely spaced leaves. Leaves oblong-lanceolate, 4–10 × 0.5–2.3 cm, apex acute. Inflorescence 10–30 cm; peduncle cylindrical, peduncle-scales several, often foliaceous, lanceolate, rachis 3–20 cm, densely many flowered; floral bracts ovate-lanceolate, 5–20 mm, longer than ovary, apex acuminate. Flowers green; pedicel and ovary arcuate, shortly beaked, 4–10 mm. Dorsal sepal oblong-ovate, 2–3.5 × 1.5–3 mm, apex obtuse; lateral sepals oblong-lanceolate, slightly oblique, 2–3.5 × 1–1.5 mm, apex obtuse. Petals ovate-lanceolate, oblique, 2–4 × 1–1.6 mm, apex obtuse. Lip lingulate, entire, 3–5 × 1–1.5 mm, ecallose, margin incurved, apex obtuse; spur pendulous, 0.8–1 mm, much shorter than ovary, apex obtuse. Column 1.5 mm; pollinia clavate, viscidium involute, horn-like; rostellum triangular with short arms, stigma transversely oblong situated below the rostellum.

#### Flowering time.

June–August.

#### Habitat.

Terrestrial in *Quercus-Abies* forests along valleys, and on grassy slopes at elevations of 1200–4000 m.

#### Distribution.

China (Gansu, Guizhou, Hubei, Sichuan, Xizang, Yunnan).

#### Specimens examined.

CHINA: **Guizhou**, Guiyang, 1300 m, 03.05.2000, *Luo Y.B., 291* (PE); **Sichuan**, Danba county, 3000 m, 6.8.1940, *Qu G. L., 7539A* (PE); Jinchuan county, 2600 m, 24.6.1958, *Li X.,77885* (PE); Mianning county, 2000 m, 25.07.1959, *Wang Z. B., 2168* (PE); Muli county, 2400 m, 1.10.1983, *Qinghai-Tibet Team*, 14895 (PE); **Tibet**, Zayu county, 3500 m, 13.7.2010, *Jin X. H.*, *Zhang S.*, *Li Z.*, *Wu B.*, *Mu X.*, *Li J.*, *Jin W. T., STET0279* (PE); **Yunnan**, Mengtze, 1875, *Hancock W., s.n.* (K); Sundian county, 2404 m, 17.8.2007, *Jin X. H*, *9190* (PE); Shangri-La, 2830 m, 29.7.1997, *Luo Y.B., 176* (9752) (PE).

### 17. Herminium glossophyllum

Taxon classificationPlantaeAsparagalesOrchidaceae

Tang & F. T. Wang, Bull. Fan Mem. Inst. Biol. Bot. 7: 127. 1936.

Figs 6H , 7H

Herminium ophioglossoides var. minus Handel-Mazzetti, Symb. Sin. 7: 1333. 1936. Type: CHINA. NW Yunnan, Lidjiang, 1914–1916, *Handel-Mazzetti H., 3975* (Holotype: WU! [WU0061504]).

#### Type.

CHINA. Yunnan, Likiang, Yangtze watershed, eastern slopes of Likiang Snow Range, 15.06.1922, *Rock J.F., 4488* (Holotype: US; Isotype: AMES! [AMES00100253]).

#### Description.

Plant 8–15 cm tall. Tubers oblong, 8–15 × 3–6 mm. Stem with 1 or 2 tubular sheaths and 1–2 leaves at base. Leaves oblong-elliptic, 3–6 × 0.4–1.5 cm, apex subacute to obtuse. Inflorescence 8–13 cm; peduncle cylindrical, ebracteate; rachis 3–7 cm, laxly many flowered; floral bracts lanceolate, 3–5 mm, much shorter than ovary, apex acuminate. Flowers yellowish green; pedicel and ovary twisted, hooked at apex, 3–5 mm. Dorsal sepal oblong-ovate, 2.5–3.5 × 1–1.6 mm, apex obtuse; lateral sepals oblong-elliptic, oblique, 3–4 × 1.2–1.8 mm, apex acute. Petals lanceolate, oblique, 3.5–5 × 0.8–1 mm, apex acute. Lip oblong, base concave and dilated, 3-lobed above middle, 3–3.5 × 2–2.2 mm, tiny globose spur, lateral lobes linear, ca. 0.8 mm; mid-lobe linear-lanceolate, 1–1.2 mm, apex acute. Column ca. 1 mm; pollinia globose; caudicles nearly indistinct or very short, viscidia involute, hornlike, rostellum with short arms, stigma attached below the rostellum, transversely oblong. Fruit oblong, 5–8 mm long.

#### Flowering time.

June–August.

#### Habitat.

Terrestrial on grassy slopes at elevations of 3100–3600 m.

#### Distribution.

China (Sichuan, Yunnan).

#### Specimens examined.

CHINA: **Sichuan**, Yanyuan county, Guangding, 3200 m, 02.07.1978, *Luo Y. B., 233* (59) (PE); Kangding county, 3600 m, 03.08.1982, *Lang K.Y., Li L.Q.,and Fei Y.,1544* (PE); **Tibet**, Zayu county, Zhuwagen, 3500–4000 m, 08.08.2012, *Jin X. H., Jin W.T.,and Xu S., 13321* (PE); **Yunnan**, Lijiang, Yulong Snow Mountain, 3200 m, 06.07.2001, *HK Kadoorie Team,1088* (PE); Shangri-La, 3200 m, 01.06.1937, *Yu T.T.,11475* (PE).

### 18. Herminium gracile

Taxon classificationPlantaeAsparagalesOrchidaceae

King & Pantl., J. Asiat. Soc. Bengal, Pt. 2, Nat. Hist. 65: 131. 1896; Perner & Luo, Orchids of Huanglong. 2007, photos on p. 38 (as “Androcorys pugioniformis”).

Figs 7K , 12

Androcorys gracilis (King & Pantl.) Schltr., Notizbl. Bot. Gart. Berlin-Dahlem 7: 397. 1920.
Androcorys ophioglossoides Schltr., Repert. Spec. Nov. Regni Veg. Beih.4: 53. 1919, **syn. nov.** Type: CHINA. Kouy-tcheou, *Esquirol J., s.n.* (Holotype: B, lost); Yunnan, Fugong county, Lumadeng, Yaping, 3400 m, *Jin X.H et Zhang L., 11078* (Neotype: PE, here chosen).
Herminium esquirolii X.H. Jin, Schuit., Raskoti & L.Q. Huang, Cladistics 2015, **syn. nov.** Not Herminium ophioglossoides Schltr.

#### Type.

INDIA. Sikkim, Lachen valley, 07.1895, *Pantling R., 397* (Lectotype: CAL! [CAL 0000000673]; Isolectotype: K! [K000880291], Isotype: P! [P00378583]).

**Figure 12.**
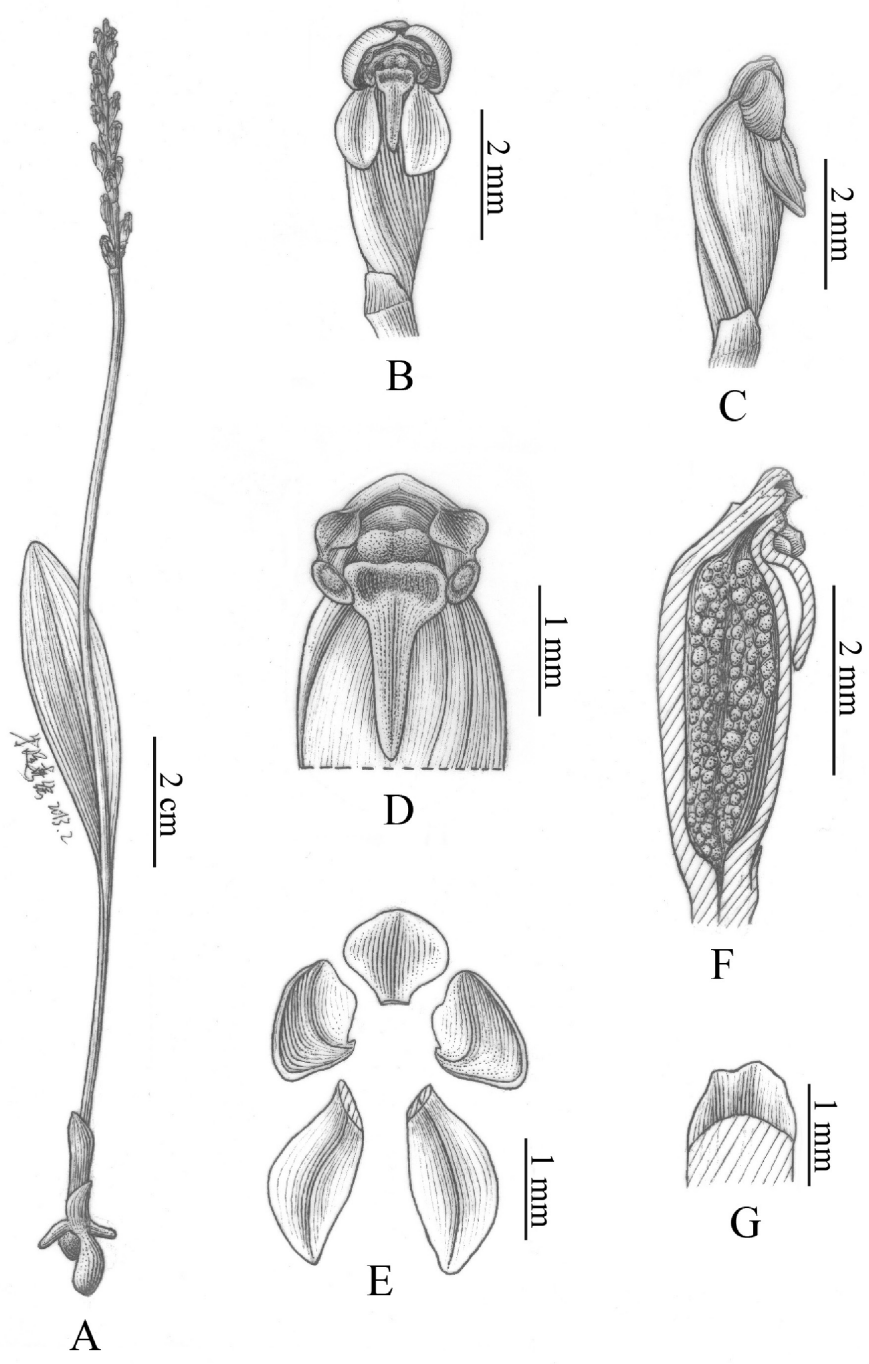
*Herminium gracile*. **A** Flowering plant **B** Flower (front view) **C** Flower (side view) **D** Ovary with column and lip **E** Dorsal, lateral sepals, petals (spread) **F** Section of the ovary **G** Floral bract.

#### Description.

Plant 15 cm tall. Tuber globose, 4–6 × 3–5 mm. Stem with 1 or 2 tubular sheaths and 1 leaf at base. Leaf shortly petiolate and sheathing, (narrowly) ovate-elliptic to oblong-spathulate, 0.7–65 × 0.4–1.0 cm, apex obtuse. Inflorescence 7–10 cm, ebracteate, rachis 0.7–4 cm long, rather laxly 3–20-flowered, floral bracts scale-like, ca. 1 mm long, apex subtruncate to almost 3-dentate. Flowers yellowish green or green, 1.5–2 mm across, pedicel and ovary fusiform, slightly beaked, 2–4.5 mm long. Dorsal sepal hooded with petals, broadly oblong-ovate, 1–1.7 × 0.4–1.5 mm, concave, apex obtuse; lateral sepals deflexed, parallel to lip, obliquely elliptic-oblong, 2–2.8 × 1–1.5 mm, apex obtuse. Petals oblong-elliptic to obliquely ovate, 1.0–2.3 × 1.0–1.3 mm, apex obtuse. Lip entire, oblong-lingulate, rather abruptly dilated at base, 1.0–2.6 × 0.3–0.5 mm, with two basal concavities separated by a longitudinal callus ridge, apex obtuse to subacute. Column ca. 1 mm tall; rostellum broadly triangular, anther locules divergent, with somewhat elongate, incurved anther-channels; pollinia ovoid, with short but distinct caudicles, viscidium globose, staminodes oblong, stigmas 2, pulvinate, attached to base of rostellum. Fruit fusiform, ca. 5 mm long.

#### Flowering time.

July–August.

#### Habitat.

Terrestrial on shady, moss-covered slopes and in alpine grassland, at elevations of 3300–4200 m.

#### Distribution.

Bhutan, China, India.

#### Note.

When describing *Herminium gracile*, King & Pantling erroneously interpreted the base of the lip with its two concavities as being part of the column, hence they and later authors believed that this species lacked the basal concavities of the lip that are seen in *H. pugioniforme*. In fact, the two taxa are quite similar in lip morphology, but differ in that *H. gracile* has well-developed caudicles (rudimentary in *H. pugioniforme*), distinct, elongated anther-channels (rudimentary anther channels), and deflexed lateral sepals that are held parallel with the lip (patent lateral sepals). In addition, *H. gracile* usually has a much more slender inflorescence with more widely spaced flowers. We find no significant differences in the protologue of *Androcorys ophioglossoides*; the fact that Schlechter stressed the uniqueness of this species makes us suspect that he did not compare it with *Herminium gracile*.

#### Specimens examined.

CHINA: **Qinghai**, Maqin county, Dawu town 3980 m, 23.07.1993, *Ho T. N. et al. 801* (PE); **Shaanxi**, Mei county, 3400 m, 30.06.1999, *Zhu C. & Xu W., 1354* (PE).

INDIA: **Sikkim**, Chumbi Valley, 3350 m, 07.1895, *Pantling R., 374A* (K); Singalila Range, 3658 m, 07.1896, *Pantling R., 374B* (K, P); Singalila Range, 3658 m, 07.1896, *Pantling R., 397* (E); North District, Lasha Chhu valley, NE of Thanggu, 18.08.1996, *Edinburgh expedition to Northern Sikkim Team, 321* (E); **West Bengal**, Darjeeling, Sandrekpho, 3650 m, 07.1881, *Gamble J. S., 9570* (K).

### 19. Herminium handelii

Taxon classificationPlantaeAsparagalesOrchidaceae

X.H. Jin, Schuit., Raskoti & L.Q. Huang, Cladistics 2015.

Figs 6K , 11D

Bhutanthera alpina (Hand.-Mazz.) Renz, Edinburgh J. Bot. 58: 102. 2001. Not Herminium alpinum (L.) Sweet, Hort. Brit.: 382. 1826 (= Chamorchis alpina (L.) Rich.).

#### Replaced name.

*Habenaria alpina* Hand.-Mazz., Symb. Sin. 7: 1336. 1936.

#### Type.

CHINA. Yunnan, between Mekong and Salwin, *Handel-Mazzetti H., 9716* (Holotype: WU! [WU0038983]; Isotypes: AMES! [00099695], E! [E00381981]).

#### Description.

Plant slender, 4–8 cm tall. Tubers globose, 0.5 × 0.4 mm. Stem at base with 2 leaves and 1–3 tubular sheaths. Leaves opposite, elliptic-lanceolate, 1.5–2.5 × 0.5–1 cm, apex obtuse or acute. Inflorescence 3–5 cm, peduncle ridged, 2–3 cm without peduncle scales, rachis 1 cm, densely 1–5-flowered; floral bracts triangular, ca 1mm. Flowers green; sepals, petals and lip margin flushed white; pedicel and ovary fusiform, 6 mm. Dorsal sepal broadly ovate, 2–3 × 1.5–2 mm, apex obtuse to subrounded; lateral sepals oblong, oblique, 2–3.5 × 2–2.5 mm, apex obtuse. Petals hooded with dorsal sepals, broadly ovate-orbicular, 1.5–2 × 1–1.5 mm, apex obtuse. Lip ovate-lanceolate, 3–3.5 × ca. 2 mm, fleshy, 3-lobed below middle; lateral lobes orbicular-ovate, very small, apex obtuse; mid-lobe lingulate-lanceolate, 0.8–1 mm, apex acute; spur cylindrical, 2–3 mm, apex obtuse. Column 1.5 mm, stout; anther with distinct connective and 2 divergent locules; rostellum erect triangular with 2 distinct arms; pollinia 2, short, to a small naked viscidium, stigma conjoined, pulvinate.

#### Flowering time.

July–August.

#### Habitat.

Terrestrial in alpine meadows and rock crevices at elevations of 4230–4300 m.

#### Distribution.

Bhutan, China, India, Nepal.

#### Specimens examined.

BHUTAN: Upper Kuru Chi district, Narim Thang, 25.07.1949, *Ludlow*, *Sherriff* and *Hicks, 21345* (BM).

CHINA: **Yunnan**, Gongshan county, 4231 m, 03.08.2013, *Jin X. H., Wang L.S., Wang Q et al., ST1778* (PE).

INDIA: **Sikkim**, Choktsering Chhu Valley, 14.07.1992, *Long*, *McBeath*, *Noltie & Watson, 362* (E).

NEPAL: **Bagmati**, Rasuwa, Near Ganesh temple, 4250 m, 20.07.2008, *Raskoti B. B., 0020089* (KATH); Langtang, Dupku Danda, 4200 m, 28.07.1986, *Miehe G.* and *S., 7000* (K).

### 20. Herminium himalayanum

Taxon classificationPlantaeAsparagalesOrchidaceae

(Renz) X.H. Jin, Schuit., Raskoti & L.Q. Huang, Cladistics 2015.

**Figure 13.**
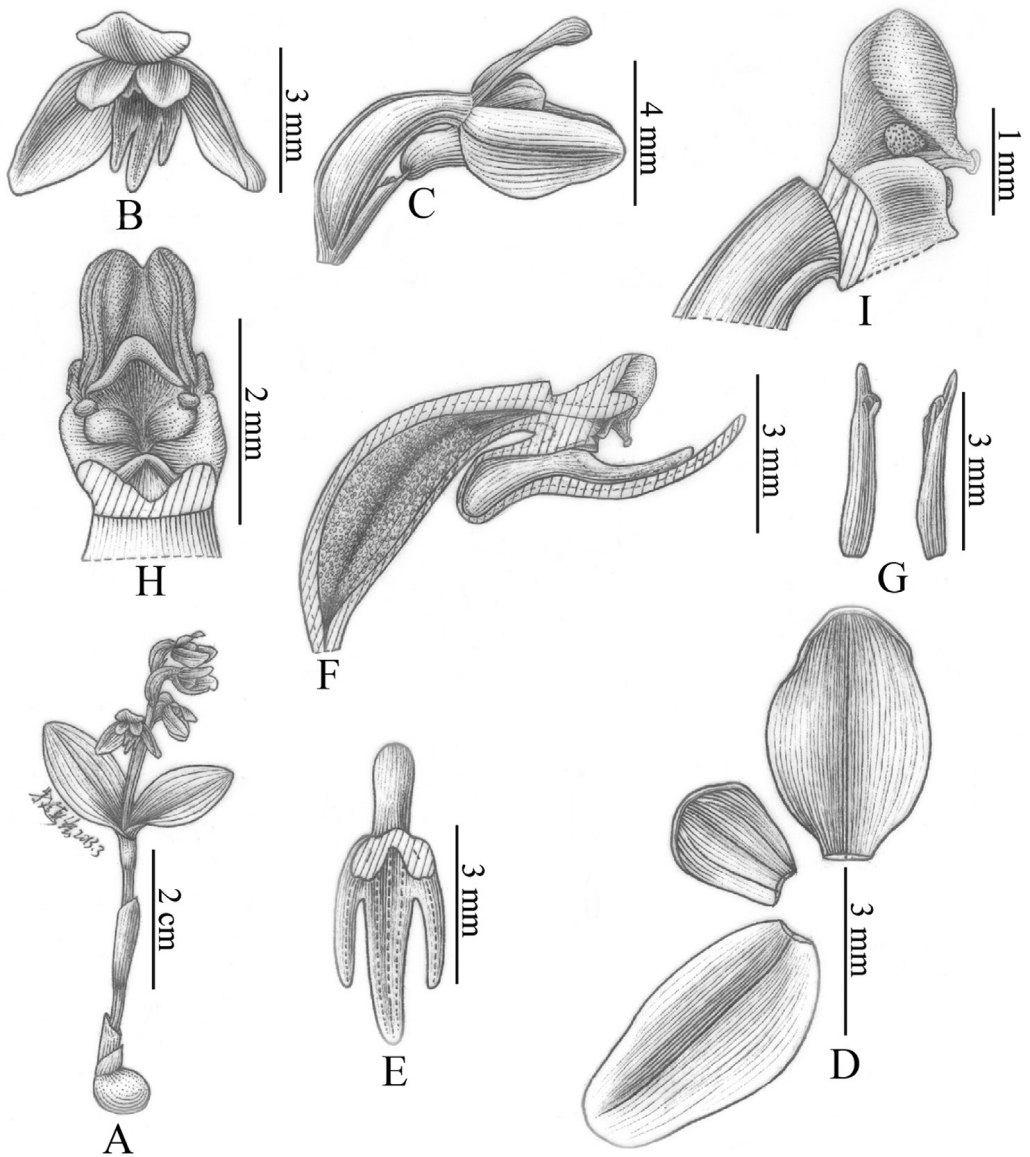
*Herminium himalayanum*. **A** Flowering plant **B** Flower (front view) **C** Flower (side view) **D** Dorsal sepal, petal and lateral sepal **E** Lip **F** Section of ovary with column and lip **G** Floral bracts **H** Column (front view) **I** Column (side view).

Figs 6M , 7M , 8K , 10F , 13

#### Basionym.

*Bhutanthera himalayana* Renz, Edinburgh J. Bot. 58: 104. 2001.

#### Type.

BHUTAN. Upper Bumthang Chu district, Pangotang-Tsampa (Chamka), *Ludlow, Sherriff* and *Hicks, 19304* (Holotype: E! [E00008024]).

#### Description.

Plant 5–6.5 cm tall. Tuber ovoid to globose, 0.7–0.9 × 0.4–1 cm. Stem with 2 leaves and tubular sheaths. Leaves subopposite, sheathed, lanceolate-elliptic, obtuse to subacute, 1.7–2.2 × 0.4–0.9 cm. Inflorescence subsecund, laxly 3–4-flowered; rachis glabrous, 1–2 cm long; floral bracts triangular-lanceolate, acute, 0.5–0.6 × 0.6–0.8 mm. Flowers 0.7–0.9 cm across; white, dorsal sepal with a green central nerve; pedicel and ovary twisted, 5–6 mm long. Dorsal sepal ovate-elliptic, subacute, 4–5 × 3 mm; lateral sepals ovate-lanceolate, obtuse to subacute, 6 × 2.8–3 mm. Petals spathulate, obtuse, cucullate at apex, 2–2.5 × 1.5–2 mm. Lip 3-lobed, spurred at base; lateral lobes linear, acute, 2–2.5 × 0.5–0.6 mm; mid-lobe linear to lingulate, obtuse, 3.5–4 × 0.5–0.8 mm; spur cylindrical-conical, obtuse, 1.5–2 mm long. Column 1.5–2 mm long; rostellum 0.25 mm long; pollinia 1.2 mm long.

#### Flowering time.

July–August.

#### Habitat.

Terrestrial on alpine grassy slopes at elevations of 3960–4570 m.

#### Distribution.

Bhutan, China.

#### Specimens examined.

BHUTAN: Thimphu district, Barshong (Parshong), 27.07.1914, *Cooper, 1979* (E); Pumo La, 08.1938, *Gould* 1294 (K); Upper Pho Chu district, Kesha La (Chesha La), 27.07.1949, *Ludlow*, *Sherriff and Hicks, 16640* (BM); Upper Bumthang Chu district, Marlung, 12.07.1949, *Ludlow*, *Sherriff and Hicks, 19413* (BM, E); Upper Kulong Chu district, Shingbe, 24.06.1949, *Ludlow*, *Sherriff and Hicks, 20401* (BM).

CHINA: **Tibet**, Yadong county, 4000 m, 01.08.2012, *Jin X.H., Jin W.T. & Xu S., 13211* (PE).

#### Note.

*Herminium himalayana* is closely related to *H. albomarginatum* but differs in having larger flowers (more than 5 mm across) and white sepals, and less than five flowers in an inflorescence.

### 21. Herminium hongdeyuanii

Taxon classificationPlantaeAsparagalesOrchidaceae

Raskoti, Phytotaxa , 98 (1): 23–26. 2013.

#### Type.

NEPAL. Chandragiri, 2200 m, 25.09.2012, *Raskoti B. B., 571* (Holotype: KATH!).

#### Description.

Plant 35–42 cm tall. Tubers subglobose, 10 × 5 mm. Stem with 2 tubular sheaths at base, upper part with 3 leaves. Leaves cauline, alternate, linear, 15–21 × 0.5–1.0 cm, apex acuminate, base sheathing. Inflorescence 20 cm; peduncle 4–6 cm, ridged, without peduncle-scales; rachis 13–15 cm, with several secund flowers; floral bracts lanceolate, 5–10 × 1–2 mm, 1-veined, exceeding or shorter than the ovary, apex acuminate. Flowers yellowish green, 2–3 mm across, secund, ovary fusiform, slightly arcuate, 5–9 mm long, ridged, apex slightly beaked; dorsal sepal suberect, ovate, 2 × 1 mm, apex sub-obtuse; lateral sepals spreading, ovate, oblique, 2.5 × 1.0 mm, apex sub-obtuse; petals forming a hood with dorsal sepal, oblong, 2.0–4.0 × 1.0 mm, apex obtuse, lip oblong, held horizontally, slightly curved, 1.0–3.0 × 0.5–1.0 mm, base dilated shallowly concave, contracted in the middle, with three ridges running from base to the midlobe, trilobed near apex; lateral lobes falcately triangular, 0.5 × 0.5 mm, apex sub-obtuse; midlobe triangular, 0.7 × 0.4 mm, apex sub-obtuse. Column ca. 1 mm; auricle ca. 0.5 mm, pollinia 0.5 mm, globose; viscidia ovoid, stigma transverse, ca. 1 mm, extending outwards from the rostellum.

#### Flowering time.

September.

#### Distribution.

Endemic to Nepal.

#### Specimen examined.

NEPAL. Chandragiri, 2200 m, 25.09.2012, *Raskoti B. B., 571* (KATH).

#### Habitat.

Terrestrial on humus-rich grassy slopes at an elevation of 2200 m.

#### Note.

This species is close to *Herminium lanceum* but differs in having smaller flowers and a ridged peduncle; the midlobe of the lip is longer than the lateral lobes, the lateral lobes are triangular and always wider than the midlobe.

### 22. Herminium humidicola

Taxon classificationPlantaeAsparagalesOrchidaceae

(K.Y. Lang & D.S. Deng) X.H. Jin, Schuit., Raskoti & L.Q. Huang, Cladistics 2015.

Frigidorchis humidicola (K. Y. Lang & D. S. Deng) Z. J. Liu & S. C. Chen, J. Fairylake Bot. Gard.6 (3): 17. 2007.
Bhutanthera humidicola (K.Y. Lang & D.S. Deng) Ormerod, Taiwania 48: 139. 2003.

#### Basionym.

*Peristylus humidicola* K. Y. Lang & D. S. Deng, Novon 6: 190. 1996 (‘*humidicolus*’).

#### Type.

CHINA. Qinghai, Magen county, Dawu Xiang, Muchang, SE of Magen, 3980 m, 05.08.1993, *Ho et al., 807* (Holotype: HNWP; Isotype: GH! [GH00243080], PE!).

#### Description.

Plant 3–4.5 cm tall. Tuber globose 10–12 × 5–8 mm. Stem sheathed at base, 2–3 leaved. Leaves crowded, ovate-elliptic to ovate-lanceolate, 2.5–3 × 1–1.5 cm, apex acute; petioles enclosed by 2 tubular sheaths forming a pseudostem 8–12 mm. Inflorescence 1.5–2 cm, equal or exceeding leaves, subcorymbose, densely 4–5-flowered. Flowers greenish-yellow; sepals white-margined apically; petals and lip becoming deep purple after pollination; pedicel and ovary 11–15 mm. Dorsal sepal suborbicular, 3–4.5 × 2.5 mm, margin denticulate toward apex; lateral sepals ovate-elliptic, 4 × 3 mm, slightly longer than dorsal sepal. Petals orbicular, 2–3 × ca.1.5 mm, fleshy, apex obtuse. Lip 3–4 mm, fleshy, spurred, 3-lobed toward its base; lateral lobes triangular, ca. 0.5 mm; mid-lobe lingulate-linear, 2–3 × ca. 0.7 mm; spur 1–2 mm long, rounded-obtuse. Column 1.5 mm, pollinia obovoid, caudicles short, viscidium disc like, rostellum triangular, shortly armed; stigmas clavate, situated below the rostellum. Fruit oblong, 6–10 mm long.

#### Flowering time.

August.

#### Habitat.

Terrestrial in alpine meadows and wet valleys at elevations of 3600–4500 m.

#### Distribution.

Endemic to China (Gansu, Qinghai, Tibet).

#### Specimen examined.

CHINA: **Qinghai**, Gande county, Along Road S101 from Gande to Maqin, 4172 m, 08.08.2014 *Jin X. H., Jin W.T. and Cui Y.,1451* (PE).

### 23. Herminium jaffreyanum

Taxon classificationPlantaeAsparagalesOrchidaceae

King & Pantl., J. Asiat. Soc. Bengal, Pt. 2, Nat. Hist. 65: 130. 1896.

Monorchis jaffreyana (King & Pantl.) O. Schwarz. Mitt. Thuring. Bot. Ges. 1:95.1949.

#### Type.

INDIA. Sikkim, near the top of Sinchal, 8600 ft, *Pantling R., 237* (Holotype: CAL; Isotypes: GH! [GH00100255], K! [K000079029, K000079030]).

#### Description.

Plant 8–20 cm tall. Tuber oblong to ellipsoidal, 10–20 × 10–15 mm. Stem 6–12 cm, with tubular sheaths at base and 2 alternate leaves near middle. Leaves oblong, 10–22 × 0.7–1 cm, apex acute. Inflorescence 6–12 cm; peduncle cylindrical, with or without a peduncle scale; rachis 10–14 cm, densely many-flowered; floral bracts ovate-lanceolate, 3–7 mm, nearly equal to ovary, apex acuminate. Flowers pale green; pedicel and ovary arcuate, 3–7 mm, apex beaked. Flower 4 mm across, greenish. Dorsal sepal broadly ovate-elliptic, concave, 2–3 × 0.5–1 mm, apex obtuse, lateral sepals ovate-lanceolate, slightly oblique, 2–3 × 0.5–1 mm apex obtuse. Petals spreading, oblong, 1.5–3 × ca. 0.2–0.5 mm, apex obtuse, lip oblong, base concave and dilated, 3-lobed above middle, lateral lobes reduced, ovate; mid-lobe triangular, apex obtuse. Column 7 mm, broad, anther discrete, parallel, pollinia obovoid, caudicles very short, viscidium globose, stigma transversely oblong situated below the rostellum.

#### Flowering.

July–August.

#### Habitat.

Terrestrial or epiphytic in humid area at elevations of 2500–3300 m.

#### Distribution.

India, Nepal.

#### Specimens examined.

INDIA. **Sikkim**, Singalila, 3048 m, 08.1892, *Pantling R., 237* (K); Sikkim, 2134 m, *Hooker J.D., 279* (K-LINDL); Singalila, 3050 m, 08.1896, *Pantling R., 237* (E); Darjeeling, “Rungbool” (?), 2150 m, 8.1878, *Gamble J. S., 3967A* (K).

NEPAL: **Koshi**, Terhthum district, Tute Deurali, 2500 m, 29.08.2007, *Raskoti B.B., 020139* (KATH).

#### Note.

There are several specimens in various herbaria labelled as *Pantling 237*, but these are all from localities and elevations not corresponding to the locality and elevation cited in the protologue. Therefore, these are not isotype specimens.

### 24. Herminium josephi

Taxon classificationPlantaeAsparagalesOrchidaceae

Rchb.f., Flora 55: 276. 1872.

Figs 6D , 7D , 8D , 8G , 9D , 10B , 11A

Monorchis josephi (Rchb.f.) O. Schwarz, Mitt. Thüring. Bot. Ges. 1: 95. 1949.
Androcorys josephi (Rchb.f.) Agrawala & H.J. Chowdhery, Kew Bull. 65: 106.2010.
Herminium grandiflorum Lindl. ex Hook.f., Fl. Brit. India 6: 129.1890, nom. invalid.
Herminium duthiei Hook. f., Fl. Brit. India 6: 130. 1890, *syn. nov*. Type: INDIA. Garwhal, near Kuris pass, 11000–12000 ft, 10.09.1885, *Duthie J. F., 4424* (Holotype: K! [K000079026]).
Peristylus duthiei (Hook.f.) Deva & H.B. Naithani, Orchid Fl. N.W. Himalaya: 181.1986, *syn. nov*.
Monorchis duthiei (Hook.f.) O. Schwarz, Mitt. Thüring. Bot. Ges. 1: 95.1949, *syn. nov*.
Herminium forrestii Schltr., Notes Roy. Bot. Gard. Edinburgh 5: 96.1912. Type: CHINA. Yunnan, Lichiang Range, *Forrest G., 2590* (Holotype: E! [E00059065]; Isotype: K! [K000079036]).
Monorchis forrestii (Schltr.) O. Schwarz, Mitt. Thüring. Bot. Ges. 1: 95.1949.

#### Type.

INDIA. Sikkim, *Hooker, 264* (Holotype: K-LINDL! [K000079043]; Isotypes: K! [K000881604], GH! [GH00100256], P! [P00378620]).

#### Description.

Plant 5–26 cm tall. Tubers ovoid, 10–15 × 5–10 mm. Stem with 2 or 3 tubular sheaths and 2 leaves at base. Leaves opposite or subopposite, oblong-elliptic, sphathulate or lanceolate, 2.5–10 × 0.5–2 cm, apex acute. Inflorescence 6–20 cm; peduncle cylindrical, ebracteate; rachis 1.5–5 cm, subdensely many-flowered; floral bracts ovate-triangular, 2–3 mm, much shorter than pedicel and ovary, apex acute. Flowers green to yellowish green; pedicel and ovary twisted, 4–6 mm, apex strongly beaked. Dorsal sepal broadly ovate, 2.5–3.5 × 1.5–2 mm, apex obtuse; lateral sepals lanceolate, oblique, 3–4.5 × 1–1.5 mm, apex obtuse. Petals ovate-lanceolate, falcate, 3–3.5 × 1–1.5 mm, apex acute. Lip entire, ovate, (3–)4–5 × (2–)3–4 mm, disk with 2 closely spaced, parallel, longitudinal keels in basal half, apex acute. Column 1–1.5 mm; pollinia obovoid; caudicles short, viscidia involute, hornlike, rostellum triangular, stigma transversely oblong, situated below the rostellum.

#### Flowering time.

July–September.

#### Habitat.

Terrestrial in *Quercus-Abies* forests, forest margins, and alpine meadows at elevations of 1200–4550 m.

#### Distribution.

Bhutan, China, India, Nepal.

#### Selected specimens examined.

BHUTAN: Phoji Ding, 3720 m, 03.08.1971, *Ramesh Bedi, 618* (K); Gasa Dist., W of Thanza, 4140 m, 04.09.2000, *Miehe G. & Miehe S., 00-350-30* (K).

CHINA: **Sichuan**, Kangding county, 3300 m, 26.07.1934, *Smith H., 10832* (PE); Muli county, 2800 m, 02.08.1937, *Yu C. T., 7550* (PE); **Tibet**, Gyirong county, 3100 m, 20.07.1975, *Qinghai-Tibet Team, 7665* (PE); Mainming, Namchabarwa NW slope, Pei No. 4 village - Nam La Tso, 3920 m, 12.09.1989, *Dickoré B, 4983* (K); Yadong, Chumbi Valley, Kala Gompa, 3350 m, 22.06.1945, *Bor* and *Kirat Ram, 19707* (K); Yatung to Gantsa, 3050–3650 m, 04.08.1936, *Spencer Chapman F., 472* (K); Zayu county, Sangjiu, 3400 m, *Jin X. H., Wang L. S., Wang Q. et al., ST2778* (PE); Zayu county, 4131 m, 29.08.2010, *Jin X. H., Liu B., Quan X., Sun M., Zhao W., SET-ET 1029* (PE); Champitang, 3650 m, 01.08.1936, *Spencer Chapman F., 823* (K); **Yunnan**, Che-tso-lo, 1200 m, 14.09.1924, *Tsai H. T., 58580* (K); Fugong county, 4000 m, 24.08.1934, *Tsai H. T., 58146* (PE); Lijiang, 3500 m, 03.08.1981, *Beijing Hengduan Mountain Team, 02547* (PE); Weixi Lisu county, 3200 m, 19.07.1981, *Beijing Hengduan Mountain Team, 01668* (PE).

INDIA. **Sikkim**, Lachen, 3353 m, 12.07.1849, *Hooker J. D., sn* (K); Lachen, 3048–4267 m, 31.07.1849, *Hooker J. D., sn* (K); Sikkim, 3048–4267 m, *Hooker J. D., 264* (K).

NEPAL: **Bagamati**, Rasuwa District, Ghodatabela, 3000 m, *Saiju H. K. and Roy B., 38* (KATH); Rasuwa District, Langtang, 3550 m, 13.07.2001, *Bhatta G. D., Karkee D. B. and Pradhan N. B., 119 B* (KATH); **Gandaki**, Kaski District, Ghorepani-Pun Hill, 3100 m, 13.07.1983, *Rajbhandari K. R., 7677* (KATH); **Janakpur**, Dolakha District, Chutra-Charikot, 3820 m, 22.06.1980, *Shakya P. R. and Roy B., 8563* (KATH); Dolakha District, Kalinchok Danda, 3600 m, 01.07.1975, *Shakya P. R., Rajbhandari K. R., Pradhan P. and Saiju H. K., 2747* (KATH); **Sagarmatha**, Solukhumbu District, Tanboche-Firiche, 4550 m, *Banerji M. L. and Shakya P. R., 5687* (KATH), Solukhumbu district, 4000 m, 22.08.1995, *Miyamoto et al., 9592370* (TI); **Seti**, Darchula District, Chheti-Nechchra, 3400 m, 22.08.1984, *Shakya P.R., Adhikari M. K. and Subedhi N. N., 8014* (KATH).

#### Note.

While the type material of *H. duthiei* is referable to *H. josephi*, albeit a small-flowered form, most specimens in herbaria labelled as *H. duthiei* are in fact *H. macrophyllum*. It is possible that some degree of hybridization occurs between the two species.

### 25. Herminium kalimpongense

Taxon classificationPlantaeAsparagalesOrchidaceae

Pradhan, Indian Orchids: Guide Identif. & Cult.1: 52–53. 1976.

Androcorys kalimpongensis (Pradhan) Agrawala & H.J. Chowdhery, Kew Bull. 65: 106. 2010.

#### Type.

INDIA. West Bengal, Kalimpong, 16.06.1974, *Pradhan U.C.*, (Holotype: Herbarium Pradhan, not located).

#### Description.

Plant 12–15 cm tall. Tuber 1–1.5 × 1 cm, globose to ovoid globose. Stem 0.8–1 cm long with 1, sessile, radical leaf. Leaf cordate, 2–2.5 cm, somewhat fleshy. Scape slender, 12–15 cm with tubular, acute, peduncle-scales, rachis with 3–4 well-spaced flowers, floral bracts 1 cm, apex acuminate. Flower half-opening, sepals subequal. Dorsal sepal 6 × 2 mm, concave, apex obtuse; lateral sepals 6 × 1 mm, apex acute. Petals 6 × 1 mm, hooded with dorsal sepal, apex acute. Lip 7 × 1.5 mm, entire, base broader than apex, spurless. Column well-developed, anther broad, pollinia ovate-globose with small viscidia, stigma transversely oblong.

#### Flowering.

August.

#### Habitat.

Not known. Collected at an elevation of 2000 m.

#### Distribution.

India.

#### Note.

Our description is based on the protologue in Pradhan (1976). Although the cordate leaf of this species as described is different from that of all other species of *Herminium*, the floral characters suggest that it belongs in this genus. However, further study is required to determine its taxonomic position.

### 26. Herminium kamengense

Taxon classificationPlantaeAsparagalesOrchidaceae

A.N. Rao, J. Econ. Tax. Bot. 25(2): 287. 2001.

Figs 9P , 10E

#### Type.

Yarlung Zangbo–Brahmaputra Region. Kameng district, Bomdila, 2500 m, *Rao A.N., 30600A* (Holotype: TIPI!).

#### Description.

Plant 15–35 cm tall. Tubers ca. 1.5 × 0.5 cm. Stem up to 18 cm tall, slender, with about 2 tubular sheaths in the lower half, 23-leaved. Leaves linear-oblong or narrowly laceolate, acuminate, 27 × 1.5 cm, sheathing at base. Inflorescence 24 cm long; rachis about 6–12 cm long, many flowered. Flowers about 7 mm long, green, floral bracts 6–10 × 1.5–2 mm, ovate-lanceolate, acuminate, longer than ovary, pedicel and ovary 6–9 mm long. Dorsal sepal ovate, obtuse, 1.5–2.5 × 1–1.5 mm,; lateral sepals obliquely ovate, 2–3 × 1.4–2 mm, apex obtuse. Petals 2–3 × 0.7–1 mm, subfalcate, oblong-lanceolate, apex subacute. Lip broadly ovate, with a circular concavity at base; 4 × 3 mm, base dilated, 3-lobed above middle, lateral lobes oblong, 1.5 × 0.5 mm, midlobe 0.5 × 0.5 mm long, triangular; lateral lobes 1.5 mm long, narrowly oblong, divergent, projected forwards. Column about 1 mm long, with 2 staminodes. Pollinia sectile, obovoid, with minute viscidia, rostellum triangular, stigma transversely oblong situated below the rostellum. Fruit oblong, 6–10 mm long.

#### Flowering.

August–September.

#### Habitat.

Terrestrial in *Quercus* forest on moist slopes at elevations of 2200–2500 m.

#### Distribution.

China, India, Nepal.

#### Specimens examined.

Yarlung Zangbo–Brahmaputra Region: Kameng district, Bomdila (2500 m), *Rao A.N., 30600AB* (CAL). NEPAL: **Koshi**, Dhankutta District, between Sidhuwa and Chitrye, 2200 m, 19.08.2012, *Raskoti B. B., 00357* (KATH).

### 27. Herminium kumaunense

Taxon classificationPlantaeAsparagalesOrchidaceae

Deva & Naithani, Orchid Fl. N.W. Himalaya: 159. 1986 (‘ kumaunensis’).

#### Type.

INDIA. Lohit district, Jachup, *Haridasan K., 3670* (Holotype: A.P. Forest Herbarium).

#### Description.

Plant upto 12 cm tall. Tubers 2, ovoid, ca. 1.0 × 0.5 cm. Leaves 2, basal, unequal, about 6 × 0.7 cm apex acute. Inflorescence 5 cm long, with lax flowered. Flowers small, green, ovary beaked and strongly hooked; floral bract lanceolate, apex acute, slightly shorter than the ovary. Dorsal sepal ovate-lanceolate, acute, ca. 2.5 mm long; lateral sepal obliquely lanceolate, apex acute. Petals oblong, acute, ca. 2 mm long, slightly contracted at the middle. Lip subequal to sepals, broadly saccate in the lower half, obscurely 3-lobed in the upper half; lateral lobes ovate, acute, shorter than the middle; mid-lobe oblong, obtuse. Column short, pollinia ranal shaped, rostellum triangular.

#### Flowering time.

August.

#### Habitat.

Terrestrial on grassy slopes at an elevation of 3000 m.

#### Distribution.

India.

#### Notes.

The description of this species is based on the protologue. Most of the characters are similar to *Herminium pygmaeum* except for the petals being constricted in the middle. More specimens and further study are necessary to determine its taxonomic status.

### 28. Herminium lanceum

Taxon classificationPlantaeAsparagalesOrchidaceae

(Thunb. ex Sw.) Vuijk, Blumea 11: 228. 1961.

Figs 5E , 6F , 7Q , 8F , 9N , 11E

Spiranthes lancea (Thunb. ex Sw.) Bakh. f. & Steenis, Syn. Pl. 2: 507.1807.
Aceras angustifolium Lindl., Edwards’s Bot. Reg. 18: t. 1525.1832. Type: NEPAL. Gossainthan, *Wallich N, 7061* (Holotype: K-LINDL! [K000883991]; Isotype K! [K000881603]).
Aceras lanceum (Thunb. ex Sw.) Steud., Nomencl. Bot., ed. 2, 1: 12.1840.
Aaceras longicrure C. Wright ex A. Gray, Mem. Amer. Acad. Arts, n.s. 6: 411 .1859. Type: JAPAN. Katonasima, *Wright C. s.n.* (Holotype not located; a hitherto unnamed specimen in K-LINDL, *Wright C., 338*, from the Loo Choo Islands, may be an isotype).
Aceras angustifolium var. longicrure (C. Wright ex A. Gray) Miquel; Prolus. Fl. Jap. 139.1866.
Platanthera angustifolia (Lindl.) Rchb.f., Otia Bot. Hamburg. 1: 39.1878.
Herminium angustifolium (Lindl.) Ridl. in Forbes, Nat. Wand. East. Archip. 519. 1885.
Herminium angustifolium var. longicruris (C. Wright ex A. Gray) Makino, Bot. Mag. (Tokyo) 10: 109. 1896.
Herminium altigenum Schltr., Repert. Spec. Nov. Regni Veg. Beih.12: 334. 1922. Type: CHINA, Tibet, Batang-Litang, Gambu gong ka, 4700 m, *Limpricht W., 2327* (Holotype: BRSL; Isotype B, lost).
Herminium minutiflorum Schltr., Repert. Spec. Nov. Regni Veg. 19: 373. 1924. Type: CHINA, Lo-fou-shan, 1000 m, 9.1917, *Levine C.O., 1479* (Holotype: B, lost).
Herminium stenostachyum Tang & F. T. Wang, Bull. Fan Mem. Inst. Biol. 7: 130. 1936. Type: CHINA. Yunnan, Sjemen, 5500 ft, *Henry A., 13556* (Holotype: K! [K000079032]).
Herminium angustifolium var. nematolobum Handel-Mazzetti, Symb. Sin.7: 1332. 1936. Type: CHINA. Yunnan, Taiwah-se, 1916, *Schoch 265* (Holotype: WU! [WU0061579]).
Monorchis angustifolia (Lindl.) O. Schwarz, Mitt. Thüring. Bot. Ges. 1: 95. 1949.
Monorchis minutiflora (Schltr.) O. Schwarz, Mitt. Thüring. Bot. Ges. 1: 95. 1949.
Herminium angustifolium var. brevilabre Tang & F. T. Wang, Acta Phytotax. Sin.1: 61.1951. Type: CHINA. Yunnan, *Tsai H.T., 61889* (Holotype: PE).
Herminium longicrure (C. Wright ex A. Gray) Tang & F. T. Wang, Acta Phytotax. Sin.1: 61.1951, not Benth. & Hook. f. (1883).
Herminium lanceum var. longicrure (C. Wright ex A. Gray) H. Hara, J. Jap. Bot. 44: 60. 1969.

#### Basionym.

*Ophrys lancea* Thunb. ex Sw., Kongl. Vetensk. Acad. Nya Handl. 21:223. 1800.

#### Type.

INDONESIA. **JAVA**, *Thunberg C. P., 21289* (Holotype: UPS!).

#### Description.

Plant 10–66 cm tall. Tubers ovoid, globose or ellipsoid, 10–15 × 5–20 mm. Stem with two tubular sheaths at base and 2–5 leaves. Leaves alternate, linear-lanceolate, 0.5–2.5 × 0.4–1.5 cm, apex acuminate. Inflorescence 5–40 cm; peduncle cylindrical, with linear-lanceolate peduncle-scales 1–7 cm; rachis 5–35 cm, laxly many flowered; floral bracts lanceolate, 3–12 mm, equal or longer than ovary, apex acuminate. Flowers pale yellowish green, pedicel and ovary 4–10 mm, slightly beaked at apex. Dorsal sepal broadly ovate, concave, 2.2–4 × 1–1.5 mm, apex obtuse; lateral sepals ovate, oblique, 2–4 × 1–2 mm, apex acute. Petals connivent with dorsal sepals, linear, 2–4 × 0.5–1 mm, apex acute. Lip oblong, 4–8 × 1–2 mm, base dilated, 3-lobed near middle, lateral lobes linear, 3 × 0.3 mm; mid-lobe oblong, 1 × 0.3 mm, apex obtuse. Column 1.4 mm; pollinia globose; caudicles short, viscidia orbicular, rostellum triangular, stigma transversely oblong, situated below the rostellum. Capsule oblong, 1 cm long.

#### Flowering time.

June–October.

#### Habitat.

Terrestrial in pine forests and on grassy slopes at elevations of 700–3743 m (–4700 m: type of *H. altigenum*), occasionally epiphytic on trunks of *Quercus* sp.

#### Distribution.

Bhutan, China, East Timor, India, Indonesia, Japan, South Korea, Malaysia, Myanmar, Nepal, Pakistan, Papua New Guinea, Philippines, Thailand, Vietnam.

#### Selected specimens examined.

CHINA: **Fujian**, Chong county, 2060 m, 11.08.1964, *Jian et al., 400644* (PE); **Gansu**, Kang county, 1200 m, 18.08.1963, *Zhang Z. Y., 16695* (PE); Choni Dist., 3350 m, *Purdom W., s.n.* (K); **Guangdong**, Boro county, Luofushan, 1000 m, 17.08.1917, *Levine C.O., s.n.* (PE); **Guangxi**, Longping, Huaping forest 750 m, 25.08.1962, (collector not mention), *Q13 (6*) (PE); **Guizhou**, Zhenfeng county, Nongchang town, peak of Longshan, 1600 m, 30.07.1996, *Luo Y.B, 118* (K, PE); **Henan**, Lushi county, 1000 m, 06.09.1935, *Liou K. M., 5454* (PE); **Hubei**, Shengnongjia, forest region, Songyu commune, Nicha river, Silong temple, 2200 m, 02.09.1976, *Shennongjia Team, 22845* (PE); without data, *Henry A., 6421* (K); Changyang, *Wilson E. H., 2236*, *6202* (K); Ichang, Patung Dist., *Henry A., 856*, *2042*, *3912* (K); **Hunan**, Sangzhi county, 1500 m, 13.07.1958, *Li H. J., 4098* (PE); **Jiangxi**, Dayu county, 1020 m, 19.06.1962, *Yue et al., 1372* (PE); **Shaanxi**, Shanyang county, 1950 m, 31.08.1952, *Wang Z. B., 16359* (PE); **Sichuan**, Nanchuan county, 1580 m, 01.08.1957, *Li G., 63155* (PE); Yajiang county, 3220 m, 13.08.1960, *Guan Z.T., 510358* (PE); Kikiang Hsien, 1050–1350 m, 11.06.1928, *Fang W. P., 1332* (K); Taiwan, Tattaka, Musha Dist., 2100 m, 09.07.1912, *Price W. R., 763* (K); Urai, 30.03.1921, *Price W. R., 320* (K); **Tibet**, Medog county, near Deergong, 1600 m, 20.08.1974, *Qinghai-Tibet Team, 4436* (PE); Mainling, Namchabarwa NW slope, above Pei No. 4 village, 3400 m, 10.09.1989, *Dickoré B, 4915* (K); Nyalam county, Lixin to Zhangmu, 2600 m, 12.10.1990, *Li B.S and Li H., 14236* (PE); Zayu county, 2000 m, 09.09.1935, *Wang Q. W., 66224* (PE); **Yunnan**, Dali (Ta-li) Hsien, 2400 m, 21.10.1937, *Tsai H. T., 53894* (K); Talifou, 04.07.1882, *Delavay J. M., s.n.* (K); Ta-li Hsien, 2400 m, 21.10.1937, *Tsai H. T., 53894* (K); Fugong county, 2555 m, 26.08.2005, *Jin X. H., 7805* (PE); near Lichiang, 3400 m, 30.07.1914, *Schneider C., 2061* (K); Menghai County, 1200 m, 15.09.1991, *Tsi Z. H., 91-537* (K); NE of Tengyueh, 3050 m, 06.1912, *Forrest G., 8364* (K).

EAST TIMOR: Fatumasse, 05.1896, *Newton F., s.n.* (K).

INDIA: Darjeeling, 2280 m, 21.08.1870, *Clarke C.B.*, (SIN); **Uttarakhand**, Kumaon, Budhi, Kali valley, 3743 m, 14.09.1900, *Inayat, 14104* (K); Garwhal near the Kuari Pass, 3353–3658 m, 10.09.1885, *Duthie J. F., 4424* (K); Rilam valley, 2743 m, 21.08.1884, *Duthie J. F., 3413* (K).

INDONESIA: **Bali**, Mt Agung, 1500–2000 m, 06.04.1936 (K); **Java**, Idjen, 1924, *Franck C. W., 569* (P); **Sumbawa**, Mt Batulanteh, 300 m, 22.04.1961, *Kostermans, 18388A* (K).

JAPAN: de ki, Koya San, 05.1888, *SC*, (P), Osumi, 08.1887, SC (P); Satsuma, Kawagiri, 07.1888, *SC*, (P), Hakodaté, 18.09.1993, *Faurie U. J., 11018* (P), Hakone, 30.08.1871, *Savatier P., 1318* (P); Nambu Prov., 1865, *Maximowicz C. J., s.n.* (K); Tosa Prov., 28.07.1888, *Elwes H. J., s.n.* (K); Nagasaki, 07.1862, *Oldham R., 808*, *848* (K); **Ryukyu Islands**, *Wright C., 338* (K-LINDL); Yona, Kunigami-son, Okinawa-hontoo, 10.04.1974, *Furuse M., 5534* (K).

KOREA REPUBLIC: **Ile Quelpaert**, Hallaisan, 10.1907, *Taquet E.J. 393* (P).

NEPAL: **Gandaki**, Lamjung District, Bahun Danda, 1500 m, 06.11.2001, *Subedi A., Chaudhary R. P. and Shakya L. R., 820* (TUCH); **Janakpur**, Ramechhap District, Patkhare to Bhandar, 2200–2300 m, 05.08.1985, *Ohba H., Kikuchi T., Wakabayanshi M., Suzuki M., Kurosaki N., Rajbhandari K. R. and Wu S. K., 8571357* (KATH); **Koshi**, Dhankuta District, Sidhuwa, 2270 m, 27.08.1989, *Grey-Wilson C., Zmarzty S., Sinnott M., Long D., McBeath R., Noltie H., and Subedi M., 27* (KATH); **Mechi**, Ilam District, Hile-Chintapu, 2933 m, 07.10.1977, *Pradhan P., Rajbhandari K. R., and Niraula R., 288* (KATH).

PHILIPPINES: **Luzon**, Bontoc Subprovince, 1911, *Vanoverbergh M, 1302* (K, P); Bontoc Subprovince, 13.07.1914, *Vanoverbergh M*, 623 (P); Benguet Subprovince, Pauai, 1911, *Santos J. K., 31930* (K, P); Mt. Boadan, 6000ft, 25.09.1921, *Ramos M. and Edano G. 71* (K, SIN).

THAILAND: Chiengmai, 1900–2100 m, 17.08.1965, *UNESCO Training Expedition, 1079* (SIN). VIETNAM: **Tokin**, Chapa, 07.1928, *Pételot P.A., 5161* (P); Lam Dong, Lac Duong, Bi Doup Mt., *HLF 5201* (LE), *HLF 5282* (LE).

### 29. Herminium latilabre

Taxon classificationPlantaeAsparagalesOrchidaceae

(Lindl.) X.H. Jin, Schuit., Raskoti & L.Q. Huang, Cladistics 2015.

**Figure 14.**
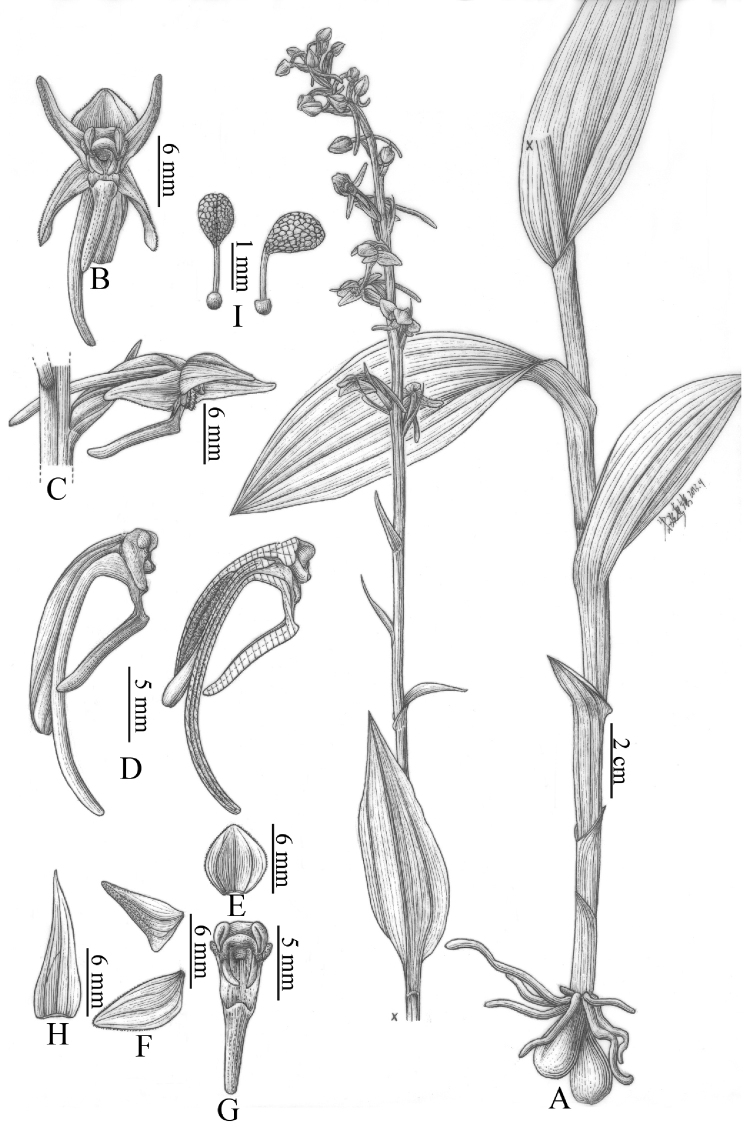
*Herminium latilabre*. **A** Flowering plant **B** Flower (front view) **C** Flower (side view) **D** Ovary with lip and spur (side view) **E** Dorsal sepal **F** Lateral sepal and petal **G** Lip **H** Floral bract **I** Pollinia.

Figs 5B , 10C , 11H , 14

Platanthera acuminata Lindl., Gen. Sp. Orchid. Pl. 289. 1835. Syntypes: NEPAL, Wallich N, 7040A (Syntype: K-LINDL; Isosyntype: K); INDIA, Kumaon, *Wallich N. (leg. Blinkworth)*, 7040B (Syntype: K-LINDL; Isosyntype: K).
Habenaria cumminsiana King & Pantl., J. Asiat. Soc. Bengal, Pt. 2, Nat. Hist. 64: 343. 1895. Syntypes: INDIA, Sikkim, Gantong, Cummins s.n. (not seen); same locality, 3650 m, 08.1894, *Pantling R., 329* (Holotype: CAL, n.v.; Isotype K! [K000974255]).
Habenaria bonatiana Schltr., Repert. Spec. Nov. Regni Veg. 12: 104.1913. Type: CHINA. Yunnan, *Maire E. E., 7558* (Holotype: B, lost; Isotype: AMES! [AMES00099726]).
Platanthera cumminsiana (King & Pantl.) Renz, Edinburgh J. Bot. 58: 117. 2001.
Platantheroides latilabris (Lindl.) Szlach., Richardiana 4: 107. 2004.
Platantheroides cumminsiana (King & Pantl.) Szlach., Richardiana 4: 106. 2004.
Habenella latilabris (Lindl.) Szlach. & Kras-Lap., Richardiana 6: 36. 2006.
Habenella cumminsiana (King & Pantl.) Szlach. & Kras-Lap., Richardiana 6: 35. 2006.

#### Basionym.

*Platanthera latilabris* Lindl., Gen. Sp. Orchid. Pl. 289. 1835.

#### Type.

INDIA. North, *Wallich N* (*leg. Blinkworth), 7040 p.p.* (Holotype: K-LINDL!, Isotype: K! [K000974253]).

#### Description.

Plant 15–68 cm tall. Tuber ellipsoid, 1–2.5 cm. Stem with 2 or 3 tubular sheaths at base and 3–4 leaves. Leaves spirally arranged, well-spaced, ovate, 5–12 × 2–5 cm, apex acuminate. Inflorescence 11–28 cm, peduncle cylindrical with 1–4 peduncle scales; rachis 4–30 cm, laxly many flowered; floral bracts lanceolate, apex acuminate, equal or longer than ovary, 1–2 cm. Flowers yellowish green; ovary and pedicel cylindrical-fusiform, arcuate, and beaked, 1–1.5 mm. Dorsal sepal connivent with petals, broadly ovate, cymbiform, 4–7 × 3–5 mm, margin ciliate, apex obtuse; lateral sepals refluxed, oblong-ovate, oblique, 5–8 × 3–4 mm, margin ciliate, apex obtuse. Petals oblong, oblique, 4–7 × 3–5 mm, apex subacute. Lip oblong-lingulate, 6–13 × 1–1.5 mm, entire, apex obtuse; disk with a conic callus in front of mouth of spur; spur cylindrical, 8–18 mm, longer than ovary. Column 0.5 mm; staminodes subsquare; anther locules nearly parallel; pollinia obovoid, with very short caudicles and orbicular viscidia; rostellum triangular; stigma lobes separate, raised, oblong to narrowly oblong, parallel, spreading on either side of base of lip. Fruit fusiform, 1–1.5 mm long.

#### Flowering time.

July–August.

#### Habitat.

Terrestrial inforest margins and grasslands at elevations of 1600–3650 m.

#### Distribution.

Bhutan, China, India, Myanmar, Nepal, Pakistan.

#### Specimens examined.

CHINA: **Sichuan**, Huei-li Hsien, 2900 m, 16.09.1932, *Yu T. T., 1499* (PE); Muli county, 2400 m, 01.10.1983, *Qinghai-Tibet Team, 14888* (PE); **Yunnan**, Gongshan county, 2662 m, 02.08.2007, *Jin X. H.,9135* (PE); Lijiang, near Yulongshan, 2900 m, 05.08.1981, *Beijing Hengduan Mountain Team, 2692* (PE); Wenshan county, 14.08.1985, *Ji Z.H, Song S. Y., Wang Z. T., 428* (PE).

INDIA: **Sikkim**, Lachen Valley, 3050 m, 07.1897, *Pantling R., 331A* (K); Singalila, 3650 m, 07.1896, *Pantling R., 331B* (K).

NEPAL: **Bagamati**, Rasuwa District, Sherpagaun, 2700 m, 06.09.1971, *Dobremez J. F., 974* (KATH); **Dhaulagiri**, Mustang District, Kalopani, 2600 m, 14.08.1975, *Joshi D. P. and Bhattacharya T. K., 75/3270* (KATH); **Karnali**, Mugu District, Rara, 3640 m, 08.1963, *Itoh K., and Rajbhandary S., 1215* (KATH).

### 30. Herminium longilobatum

Taxon classificationPlantaeAsparagalesOrchidaceae

S.N. Hegde & A.N. Rao, Himalayan Pl. J. 1(2): 47. 1982.

Fig. 10F

#### Type.

Yarlung Zangbo–Brahmaputra Region, Towang, 2800 m, *Hegde, 3208-A* (Holotype: TIPI!).

#### Description.

Plant 30–50 cm tall. Tubers ovoid, globose to oblong. Stem with 2 tubular sheaths at base and few well-spaced leaves near middle. Leaves 8–20 × 0.5–1.5 cm, linear to oblong, acuminate. Inflorescence 8–30 cm; peduncle cylindrical, with peduncle-scales 0.5–2.4 cm; rachis 5–18 cm, densely many flowered; floral bracts ovate-lanceolate, 1.5–5 mm, shorter than ovary, apex acuminate. Flowers pale green; ca. 4 mm across, pedicel and ovary arcuate, 4–7 mm, shortly beaked. Dorsal sepals 2.0 × 1.5 mm, concave, ovate, apex subacute. Petals lanceolate, 2.0 × 1.5 mm, apex subacute. Lip oblong, 3-lobed, with a callus at base, midlobe very short, lanceolate, obtuse; lateral lobes 10 mm long, more than 10 times longer than the midlobe, filiform, coiled. Column 1 mm, short; anther cells 2, parallel; staminodes 2, lateral; rostellum 2-lobed; stigmas 2, transversely oblong, divergent. Fruit oblong, 7 mm long.

Flowering: August–September.

#### Habitat.

Terrestrial in oak forest at elevations of 2500–2800 m.

#### Distribution.

China, India, Nepal.

#### Specimen examined.

Yarlung Zangbo–Brahmaputra Region: **Kameng** district, Towang, *Hegde, 3208 A-C* (Orchid Herbarium, Tipi); Phudong-Dirang, Hajra 54808 (ASSAM). NEPAL: **Sagarmatha**, Solukhumbu district, below Sete, 2500 m, *Raskoti B. B., 01299* (KATH).

#### Note.

*Herminium longilobatum* is similar to *Herminium lanceum* but the lateral lobes of lip are much longer (10 vs. 3 mm) and the lip often has a callus at the base.

### 31. Herminium mackinnonii

Taxon classificationPlantaeAsparagalesOrchidaceae

Duthie, Journ, As. Soc. Beng. Nat. His.71:44.1902.

Monorchis mackinnonii (Duthie) O. Schwarz, Mitt. Thüring. Bot. Ges. 1: 95.1949.

#### Type.

INDIA. Lehari-Garhwal, near Mussoorie, 6000 ft, 30.08.1901, *Mackinnon P. W., 25421* (Holotype: K! [K000079024]; Isotype: AMES! [AMES00100258]).

#### Description.

Plant 15–30 cm tall. Tuber oblong, 7–1.5 × 5–12 mm. Stem with tubular sheaths and 2 basal, subopposite leaves. Leaves linear-lanceolate, 10–16 × 1–1.5 cm, acute to acuminate. Inflorescence 10–20 cm, cylindrical, with or without peduncle-scales, rachis 10–15 cm, subdensely many-flowered, floral bracts 5 mm, broadly lanceolate, acuminate, shorter than the ovary. Flowers subsecund, spreading, 5–10 mm across, ovary straight, slightly beaked at 6 mm. Dorsal sepals ovate-oblong, 2.5–3.5 × 1.5–2 mm, obtuse; lateral sepals obliquely ovate-oblong, 2–3 × 1.5–2 mm, acute. Petals linear-lanceolate, 3 × 1 mm, apex acute. Lip deflexed, 3-lobed above middle, basal part concave, dilated and with a small concavity atthe base, lateral lobes linear, apex acute, midlobe lanceolate, apex obtuse. Column 1 mm, anther cells parallel, pollinia ovate ellipsoid, caudicles very short, viscidium discoid, rostellum triangular armed, stigma 2, transversely oblong, lying beneath the rostellum.

#### Flowering time.

July–August.

#### Habitat.

Terrestrial or epiphytic on trunk or large branches of oak trees at elevations of 2400–2700 m.

#### Distribution.

India, Nepal.

#### Specimens examined.

NEPAL: **Koshi**, Bhuje Danda to Tamur River, 2700 m, 03.08.1989, *Grey-Wilsonet al., 135* (KATH); **Mechi**, Ilam District, Hille-Chintapu, 2450 m, 07.10.1977, *Pradhan P., Rajbhandari K. R. and Niraula R.*, 289 (KATH).

### 32. Herminium macrophyllum

Taxon classificationPlantaeAsparagalesOrchidaceae

(D. Don) Dandy, J. Bot. 70: 328. 1932.

Figs 6G, P , 7G , 8P , 9F, G , 11B

Herminium congestum Lindl., Edwards’s Bot. Reg. 18: t.1499.1832. Type: NEPAL. Gossain Than, 1821, *Wallich N., 7068* (Holotype: K-LINDL! [K000079039]; Isotypes K-WALLICH! [K001126683], K!, E! E00188162], BM! [BM000034357], E! [E00188162]).
Spiranthes macrophylla (D. Don) Spreng., Syst. Veg. 3: 708.1826.
Peristylus macrophyllus (D. Don) Lawkush, Vik. Kumar & Bankoti, Indian J. Forest. 36(3): 388. 2013.
Peristylus duthiei var. inayatii Deva & H.B. Naithani, Orchid Fl. N.W. Himalaya 185.1986, *syn. nov.* Type: INDIA. Uttarakhand, Pithoragarh District, Kumaon, Ralam Valley; 19.8.1900; *Inayat 24103* (Holotype: DD).

#### Basionym.

*Neottia macrophylla* D. Don, Prodr. Fl. Nepal. 27. 1825

#### Type.

NEPAL. 1819, *Wallich N. s.n.* (Holotype: BM! [BM000034355]).

#### Description.

Plant 8–25 cm tall. Tubers ovoid, 6–15 × 5–10 mm. Stem with 2 or 3 tubular sheaths and 2–3 leaves at base. Leaves oblong-elliptic or oblanceolate, 3–10 × 0.6–2 cm, apex subacute to obtuse. Inflorescence 6–15 cm; peduncle cylindrical, without or rarely with 1 foliaceous peduncle-scale; rachis 2–9 cm, subdensely flowered; floral bracts ovate-triangular, 1–3 mm, shorter than ovary, apex acute. Flowers white or green, or green with white tips; pedicel and ovary twisted, hooked at apex, 3–6 mm. Dorsal sepal ovate, 0.5–2 × 0.8–1.2 mm, apex obtuse; lateral sepals oblong, 1.5– 2 × 0.5–1 mm, apex acute. Petals obliquely ovate-triangular, fleshy, 1.5–2 × 0.7–1.2 mm, apex acute. Lip ovate-lanceolate, 1.6–3 × 0.6–1 mm, with spur like globose saccate base, dilated, disk at base concave, with a single raised crest, indistinctly 3-lobed at base; lateral lobes obscure, margin crenulate, mid-lobe ovate-triangular, 0.5 × 0.5 mm, apex acute. Column ca. 1 mm; pollinia subglobose; caudicles short, viscidia involute, hornlike, rostellum triangular, stigma transversely oblong, situated below the rostellum.

#### Flowering time.

June–August.

#### Habitat.

Terrestrial in *Quercus-Abies* forests and on alpine grassy slopes and meadows at elevations of 2400–4620 m.

#### Distribution.

Bhutan, China, India, Nepal, Pakistan.

#### Notes.

This species has often been confused with *H. duthiei*, which we consider to be a synonym of *H. josephi*. Most specimens identified as *H. duthiei* in the past are referrable to *H. macrophyllum*.

#### Specimens examined.

BHUTAN: Pele-la and Ri-Tang, 2500–3350 m, 30.06.1938, *Gould R. J., 735* (K); Sela-la, 3962 m, 30.06.1971, *Ramesh Bedi, 349* (K); Gasa, Robluthang, 4620 m, 25.07.2000, *Miehe G. and Miehe S., 00-239-10* (K); Bumthang, between Gorsuem and Tsochen Chen, 3660 m, 22.06.2000, *Miehe G. and S., 00-119-02* (K); Paro, Chomolhari Chu, 4060 m, 05.07.2000, *Miehe G. and Miehe S., 00-161-09* (K).

CHINA: **Tibet**, Gyirong county, 3800 m, 16.07.1975, *Qinghai-Tibet Team, 6388* (PE); Gyirong county, 3400 m, 22.06.1972, *Tibetan Herb Survey Team, 494* (PE); Champilang, 3650 m, 01.08.1936, *Spencer Chapman F., 818* (K).

INDIA. **Sikkim**, Lachen, 07-08.1849, 3350–3650 m, *Hooker J. D., 265* (K, K-LINDL); sine loc., 3950–4250 m, *Hooker J.D., 266* (K); Nathula Range, 4300 m, 28.06.1985, *Lachuypa S. T., 31* (K); Singalila, 3050 m, 08.1896, Lachen Valley, 3650 m, 08.1895 & 97, Lachong Valley, 2750 m, 07.1897, *Pantling R., 384* (K); Chemathang, 4250, 25.06.1983, *Starling et al., A.G.S.E.S. 138* (K); **Uttarakhand**, Garhwal, Kidar Kantha, 4570 m, 11.06.1904, *Drummond J.R., 22792* (K).

NEPAL: **Dhaulagiri**, Mustang District, Thorong Phedi 4500 m, 20.08.1999, *Mikage M.*, *Yoshimitsu M., Kaneda A., Mouri C., Tatsukawa S., Asada Y., Senoo M.,9962979* (KATH); **Karnali**, Mugu District, Chankheli lekh, 3250 m, 13.08.1985, *Shakya P. R.*, *Subedi N. N.* and *Uprety R.*, *8600* (KATH); Gandaki Zone Kaski District, Birethanti-Ghorepani, 3100 m, 12.07.1983, *Rajbhandari K. R.,7667* (KATH); Langtang, Palpa, 3550 m, 07.09.1986, *Miehe G. and S., 11011 and 11034* (K); Serpagaon–Langtang, 2750 m, 13.07.1967, *Malla, s.n.* (K); Taplejung, Ghunsa, 3400 m, 26.09.1985, *Damant, 96* (K); Ringmo–Taksindhu, 3000 m, *Banerjee and Shakya P.R., 5567* (K); Kunde, Khumbu, 3950 m, 27.07.1966, *Schilling A.D., 948* (K); east of Jumbesi, above Ringmo, Sola, 2800 m, 22.07.1966, *Schilling, A.D., 1032* (K); Yalung, 4100 m, 22.06.1969, *Shrestha T.B., 15837* (K); Kalingchok, 12.09.1970, *Kanai, Chuma and Nagano, 674065* (K); environs of Ghunsa, 3520 m, 08.09.1989, *Crawford S. et al., KEKE 431* (K); Tharepati, east of Kathmandu, 3200 m, 27.06.1973, *Grey-Wilson and Phillips, 179* (K); Lisue, .3200 m, *Itoh and Rajbhandari, 1118* (K).

### 33. Herminium mannii

Taxon classificationPlantaeAsparagalesOrchidaceae

(Rchb.f.) Tang & F.T. Wang, Bull. Fan Mem. Inst. Biol. 7: 128. 1936.

Figs 6Q , 10H

Habenaria gracillima Hook.f., Fl. Brit. India 6: 163. 1890. Type: INDIA. Khasia Hills, Munnipore, 4000–5000 ft, *Clarke C. B. s.n.* (not found).
Peristylus gracillimus (Hook.f.) Kraenzl., Orchid. Gen. Sp. 1: 513. 1898.
Habenaria duclouxii Rolfe, Notes Roy. Bot. Gard. Edinburgh 8: 25. 1913. Type: CHINA. Yunnan, *Ducloux F., 226* (Lectotype, here chosen: K! [K000827046W]). Syntype: Yunnan, western slopes of the Tsan-Shan Range near head of Yang-pi pass, 9.1905, *Forrest G., 906* (Syntype: E! [E00381984]).
Habenaria spiranthiformis Ames & Schltr., Repert. Spec. Nov. Regni Veg. Beih.4: 52. 1919. Type: CHINA. Yunnan, *Maire E. E., 2616* (Holotype: AMES! [AMES00100097]).
Platanthera mannii (Rchb.f.) Schltr., Repert. Spec. Nov. Regni Veg. Beih. 4: 114. 1919.
Herminium yuanum Tang & F.T. Wang, Bull. Fan Mem. Inst. Biol. 7: 129. 1936. Type: CHINA. Sichuan, Li county, 2900 m, 17.09.1932, *Yu T. T., 1543* (Holotype: PE! [PE00027291]).
Herminium spiranthiforme (Ames & Schltr.) Tang & F.T. Wang, Acta Phytotax. Sin.1: 62. 1951.
Peristylus mannii (Rchb. f.) Mukherjee, Notes Roy. Bot. Gard. Edinburgh 21(3): 153. 1953.

#### Basionym.

*Coeloglossum mannii* Rchb.f., Linnaea 41: 54. 1876.

#### Type.

INDIA. Assam, Khasia Hills, 5000 ft; *Mann s.n.* (Holotype: W).

#### Description.

Plant 10–43 cm tall. Tubers oblong, 1–2.5 × 0.5–1 cm. Stem with 2–4 leaves and 2 tubular sheaths at base. Leaves linear, 3–14 × 0.2–0.4 cm, apex acuminate. Inflorescence 10–37 cm; peduncle cylindrical, peduncle-scales 1–5, lanceolate, 2–20 mm; rachis 4–16 cm, laxly many-flowered; floral bracts ovate-lanceolate, 2–4 mm, shorter than ovary, apex acuminate. Flowers pale yellowish green; ca. 3 mm across, pedicel and ovary 2–5 mm. Dorsal sepal ovate, concave, 2–2.5 × 0.8–1 mm, apex obtuse; lateral sepals oblong-lingulate, 2–2.5 × 0.8–1 mm, apex obtuse. Petals hooded with dorsal sepals, obliquely ovate-lanceolate, 2–2.5 × ca. 0.8 mm, apex obtuse. Lip oblong-ovate, 2.5–3 × 0.8–1 mm, base deeply concave, ecallose, 3-lobed near middle; lateral lobes oblong, 0.8–1.5 mm; mid-lobe oblong, slightly attenuate, 1–2 mm, spur pendulous, scrotiform, 1–1.5 mm, apex obtuse. Column 0.3–0.5 mm; pollinia globose, caudicles short, viscidia ellipsoid, rostellum triangular, shortly armed, stigma transversely oblong-clavate, extended outwards at base of lip.

#### Flowering time.

August–October.

#### Habitat.

Terrestrial in forest margins and on grassy slopes at elevations of 1700–3400 m.

#### Distribution.

China, India, Myanmar, Nepal.

#### Specimens examined.

CHINA: **Sichuan**, Danba county, 3000 m, 06.08.1940, *Qu G.L.,7539A* (PE); Jinchuan county, 3100 m, 15.06.1958, *Li X., 77748* (PE); Luding county, 1300 m, 12.07.1982, *Lang K.Y.*, *Li L.Q.*, *Fei Y., 624* (PE); Mianning, 1800 m, 30.09.1938, *Yu T. T., 17814* (PE); Mianning, 2000 m, 25.07.1959, *Wang Z.B., 2168* (PE); Muli, 2300 m, 18.07.1937, *Yu T.T., 7319* (PE); Yajiang county, 3080 m, 30.07.1960, *Guan Z.T., 41–0728* (PE); **Yunnan**, Gongshan county, 3337 m, 11.09.2005, *Jin X. H.,7983* (PE); Songming County (Sung-Ming KuoTung), 2200 m, 10.10.1950, *Mao P.I., 161* (PE); mid-W Yunnan, 1520 m, 09.1924, *Forrest G., 25104* (K); Wenshan county, Bozhushan, Xiaoheijing, 2400 m, 31.07.1993, *Shui Y. M., 003196* (PE).

INDIA: **Assam**, Khasi Hills, Mawphlang, 1800 m, 31.07.1954, *Chand T. R., 7890* (K); **Meghalaya**, 11.09.1984, *Naithani, H. B.*, 1175 (DD); **Nagaland**, Kegurma Edge, 2100 m, 10.11.1885, *Clarke C. B., 61858* (K).

NEPAL: **Koshi**, Taplejung, Guphapokhari at 2900 m, 29.08.2007, *Raskoti B. B., 250* (TUCH).

### 34. Herminium monophyllum

Taxon classificationPlantaeAsparagalesOrchidaceae

(D. Don) P. F. Hunt & Summerh., Kew Bull. 20: 51. 1966.

Figs 5G , 6E , 7E , 8E , 9E , 10D

Androcorys monophylla (D. Don) Agrawala & H. J. Chowdhery, Kew Bull. 65: 106. 2010.
Herminium gramineum Lindl., Gen. Sp. Orchid. Pl.: 305. 1835. Type: NEPAL. Precise locality unknown, 1821, *Wallich N., 7413* (Holotype: K-LINDL! [K000079040]; Isotypes: K-WALLICH! [K001127309 & K001127310]).
Spiranthes monophylla (D. Don) Spreng., Syst. Veg. 3: 709. 1826.
Monorchis monophylla (D. Don) O. Schwarz, Mitt. Thüring. Bot. Ges. 1: 95. 1949.

#### Basionym.

*Neottia monophylla* D. Don, Prodr. Fl. Nepal. 27. 1825.

#### Type.

NEPAL. Precise locality unknown, 1819, *Wallich N. s.n.* (Holotype: BM! [BM000034436]).

#### Description.

Plant 6–15 cm tall. Tuber globose, 5–9 × 4–6 mm. Stem slender, 2.5–7; sheaths tubular, 1–2.5 cm long, with 1 leaf at base. Leaf linear-oblong, apex acute, 3.5–5.5 × 0.2–0.6 cm. Inflorescence 4–10 cm, laxly to subdensely many-flowered, rachis glabrous, 2–6 cm, floral bracts lanceolate, apex trifid, 3–8 × 1–1.5 mm. Flowers subsecund, yellowish green, 4–7 mm across, pedicel and ovary arcuate, 3–4.5 mm long, apex beaked. Dorsal sepal broadly ovate-oblong, 2–2.5 × 1.2–1.5 mm, apex obtuse; lateral sepals ovate, 2–3 × 1–1.2 mm, apex obtuse. Petals linear, falcate, 2.5 × 0.8 mm, apex obtuse. Lip ovate, concave, entire, 2.5 × 1.5 mm, apex obtuse. Column 2–4 mm tall, pollinia clavate, caudicles short, viscidium disc like, rostellum triangular, stigma transversely oblong, situated below the rostellum.

#### Flowering time.

July–August.

#### Habitat.

Terrestrial in open forest or forest margins at an altitude of 1000–2000 m.

#### Distribution.

Bhutan, India, Nepal.

#### Specimens examined.

INDIA: **Himachal Pradesh**, Simla, 1800 m, *Drummond J. R. (leg. Donie J. M.) s.n.* (K); **Uttarakhand**, below Mussoorie, 1370 m, 01.08.1932, *Stewart R. R., 12317* (K).

NEPAL: **Bagmati**, Lalitpur district Godawari 1820m, 05.08.1978, Anon, 297 (KATH); Sindhupalnchok District Bhungloo, 1850 m, 11.09.1964, *Benerji M. L., Shrestha T. B. and Upadhyaya A. V., 2656* (KATH); Kathmandu district, Sivapuri, 1880 m, 01.08.1979, *Dahal S. and Dawadi N., 834* (KATH); **Mahakali**, Baitadi District, Ganna-Nala Bagar, 1000 m, 15.07.1984, *Shakya P. R., Adhikari M. K. and Subedi M. N., 7859* (KATH); **Seti**, Doti District, Doti-Baga lekha, 1950 m, 30.06.198, *Shakya P. R., Sharma L. R. and Amatya K. R., 6223* (KATH); Ganesh Himal, Haku, 1700 m, 07.07.1984, *Farille M., Jordan D. and Lachard G., 847034* (K); Kumaon, near Sasa, 2100–2400 m, 14.07.1886, *Duthie J. F., 5997* (K); Kumaon, Shania, Sargu Valley, 31.07.1900, *Inayat, 24107* (K).

### 35. Herminium monorchis

Taxon classificationPlantaeAsparagalesOrchidaceae

(L.) R. Br. in W. T. Aiton, Hortus Kew. 5: 191. 1813.

Figs 5A , 6A , 7A , 8A , 9A , 10A

Orchis monorchis (L.) Crantz, Stirp. Austr. Fasc., ed. 2, 2: 478. 1769.
Epipactis monorchis (L.) F.W. Schmidt, Samml. Phys.-Ökon. Aufs.1: 246. 1795.
Arachnites monorchis (L.) Hoffm., Deutschl. Fl. Bot. Taschenb. 4: 179.1804.
Satyrium monorchis (L.) Pers., Syn. Pl. 2: 507. 1807.
Monorchis herminium O. Schwarz, Mitt. Thüring. Bot. Ges. 1:95. 1949.
Herminium alaschanicum Maxim. var. tanguticum Maxim., Bull. Acad. Imp. Sci. Saint-Pétersbourg 31: 105. 1887. Type: China, Gansu, Tangut, 3050 m, 1880, *Przewalski N. M., s.n.* (Holotype: LE, n.v.; Isotype: K!).
Herminium tanguticum Rolfe., J. Linn. Soc., Bot. 36: 51. 1903. Type: China, Gansu, Tangut, 3050 m, 1880, *Przewalski N. M., s.n.* (Holotype: K! [K000079031]; Isotype: LE, n.v.).
Herminium haridasanii A.N.Rao, J. Econ. Taxon. Bot. 16: 725. 1992, syn. nov. Type: Yarlung Zangbo–Brahmaputra Region, Lohit Distr., Hot Springs, 3000 m, *Haridasan* 3764-A (Holotype: Orchid Herbarium Tipi, n.v.).

#### Basionym.

*Ophrys monorchis* L., Sp. Pl. 2: 947.1753.

#### Type.

Without country and precise locality, *Anon, s.n.* (Lectotype: LINN! [LINN-HL1056–22]).

#### Description.

Plant 5–38 cm tall. Tubers globose, 5–10 × 4–10 mm. Stem with 1 or 2 tubular sheaths at base and (1–)2–3leaves. Leaves subopposite to well-spaced, elliptic or elliptic-lanceolate, 2–13 × 0.5–2.9 cm, apex acute. Inflorescence 3–38 cm; peduncle cylindrical, with 0–4 ovate-lanceolate to linear peduncle-scales (sometimes leaf-like), rachis 2–24 cm, densely to sublaxly many flowered; floral bracts linear-lanceolate, 2–7 mm, shorter than or as long as ovary, apex acuminate. Flowers yellowish green; pedicel and ovary twisted, beaked and strongly hooked at apex, 3–7 mm. Dorsal sepal ovate-lanceolate, 2–3 × 1–1.5 mm, apex obtuse; lateral sepal ovate-lanceolate, slightly oblique, 2–2.7 × 0.8–1.1 mm, apex subacute. Petals rhombic-caudate, falcate, fleshy, 2.5–4.7 × 1–1.5 mm, apex acute. Lip oblong, 2–5 × 1.1–1.5 mm, base concave forming an almost spur-like sac, 3-lobed near middle; lateral lobes linear-triangular, 0.9–1.2 × 0.4 mm, apex acute; mid-lobe linear-triangular, 2.2–3 × 0.4–1 mm, apex (sub)acute. Column 0.8 mm; pollinia subglobose; caudicles very short, viscidia large, involute, hornlike, rostellum triangular, stigma transversely oblong, situated below the rostellum. Fruit oblongoid, suberect, c. 9 mm long.

#### Flowering time.

(June–)July–August.

#### Habitat.

Terrestrial in broad-leaved or coniferous forests, extensively grazed meadows on chalk and limestone, swampy grassland, alpine meadows and valleys; in Asia at elevations of 600–4700 m; in Europe (including the Caucasus) 0–2400 m.

#### Distribution.

China, India, Japan, Korea, Mongolia, Nepal, Pakistan, Russia, C and W Asia, most parts of Europe, excluding the Mediterranean zone and the far North.

#### Selected specimens examined

(only a small sample of the numerous European specimens studied are cited below). AUSTRIA: Tirol, Falzthurntal, 1100 m, 10.07.1936, *Milne-Redhead, E. 2380* (K).

CHINA: **Gansu**, Xiahe county, 2400 m, 07.07.1937, *Fu K.T., 1078* (PE); Central Gansu, Lien hoa shan between Taochow and Totao, 3050 m, 14.07.1920, *Rock J. F., 12736* (K); **Hebei**, Santaoho, 1700 m, 23.08.1934, *Wang C.W., 62296* (PE); **Henan**, Yuchaishan, Honan, 1150, 03.07.1932, *Hao K.S., 3703* (PE); **Jilin**, Jiao county, near Peitayang, 500 m, 14.07.1931, *Kung H.W., 1848* (PE); **Inner Mongolia**, Nearby HaiLa Er Shi, 600 m, 04.07.1951, *Wang Z. D., 1167* (PE); **Qinghai**, Datong county, near Guanghui Temple, 1950 m, 25.07.1936, *Liu K. M.,6077* (PE); **Shaanxi**, Mei county, Taibeishan, Tangyu, Shangbansi, 2700 m, 02.07.1999, *Zhu C., Wang X,1428* (PE); **Shandong**, Taishan, 1800 m, 18.07.1956, *Chang C.,41* (PE); **Shanxi**, Ningwu county, Dong zhai,near Fen river, 1670 m, 15.07.1957, *Liu J., 1598* (PE); **Sichuan**, Xiangcheng Xian, vicinity of Reda twon, Rizhao Shen Shan, 3600–3850 m, 15.07.2004, *Boufford D. E. et al., 30665* (K); Jiulong Xian, Tanggu Cun, N of Tanggu Xiang village along Jiulong - Kangding Rd, 3900 m, 21.07.2005, *Boufford D. E. et al., 33293* (K); **Tibet**, Bomi county, 2700 m, 12.07.1965, *Zhang Y.T. and Lang K. Y., 552* (PE); Lhasa, north of Naiguodong, 4300 m, 17.08.1965, *Zhang Y.T. and Lang K.Y., 1633* (PE); Mainling county, 3100 m, 29.07.1974, *Qinghai-Tibet Team, 3727* (PE); Champilang, 3650 m, 01.08.1936, *Spencer Chapman F., 455* (K); **Yunnan**, Shangri-La, 3000 m, 06.07.1996, *Luo Y.B., 71* (PE); Shangri-La, 3200 m, 25.07.1937, *Yu C. T., 12433* (PE).

ESTONIA: Tartu, Emajogi River, 2 km east of Ropka, 20.06.1930, *Sirgo V. s.n.* (K).

FRANCE: Seine-et-Oise, Bois du Coudray near Marles, 20.06.1852, *Maillard A. s.n.* (K)

GERMANY: Würtemberg, Donnstetten, 07.1876, *Kemmler s.n.* (K).

INDIA: **Himachal Pradesh**, Spiti, Tabo, 3050 m, 18.08.1933, *Gill, H.A.C., 2043* (K); Lahul, Kyelang, 3140 m, 04.07.1941, *Bor N.L., 14970* (K); **Kashmir**, Jilail, 2750 m, 25.08.1876, *Clarke C.B., 30737* (K); **Sikkim**, Nathong, 13.07.1877, *King G., 4365* (K); **Uttarakhand**, Kumaon, Barphu, Gari Valley, 12.08.1900, *Inayat, 24106* (K).

ITALY: Cuneo, S. Anna di Valdieri, 1700 m, 08.07.1906, *Ferrari, E. and Gola, G.* (K).

JAPAN: **Khusiro**, 08.08.1893, *Faurie U.J., 10809* (K, P).

SOUTH KOREA: Ouen san, 07.1906, *Faurie U.J., 242* (P).

MONGOLIA: Ik’e lala, 26.06.1924, *Licent, E. 7508* (K); Orkhon Valley, 14.07.1899, *du Chazaud 91* (P).

NEPAL: **Janakpur**, Dolakha District, Kalinchok, 3600 m, 10.07.1985, *Bhattrai N., Shrestha M., Pradhan N.*, and *Shakya S., 10A* (KATH); **Koshi**, Sankhuwasabha District, Ta Dasa to Makalu Base Camp, 4000–4680 m, 27.08.1988, *Suzuki M., Narushashi N., Kurosaki S., Kadota Y., Subedhi M. N., Minaki M., Noshiro S. and Ikeda H., 8850* (KATH); just north of Shimen, 4000 m, 05.08.1973, *Grey-Wilson and Phillips, 524* (K).

ROMANIA: Transylvania, Cluj, Ratacita Valley, 800 m, 24.07.1949, *Tretiu T. and M. 3098* (K)

RUSSIA: Pskow Prov., Ostrow Dist., Kudep River near Kosly, 07.07.1898, *Dzeiwer A. and Puring N. s.n.* (K); Balkaria, Khyzny-su, Ogary-Kishlyk, 2050 m, 03.07.1927, *Bush E. and N. s.n.* (K); Minnusinsk Dist., 06.06.1913, *Kuznetsov I. V. 2553* (K); Ussuri, *Maack R. K. s.n.* (K, P); Siberia, Altai Mts., *von Ledebour C. F. s.n.* (P); Siberia, Irkutsk, 06.1888, *Karo F. K. s.n.* (P); northern shore of Lake Baikal, 01.07.1855, *Radde G. s.n.* (K); Dauria, 1831, *von Fischer K. F. s.n.* (P); Manschuria, Amur River near Bachurowa, 26.06.1895, *Komarov V.* 439 (K); Amur region, Blagowjestschensk, 07.1898, *Karo F 211* (K); Coast of Manschuria (Lat. 44–45 N), 07.08.1859, *Wilford C. 1168* (K); SE Manschuria, near Possiet Bay, 1860, *Maximowicz C. J., s.n.* (K); Khabarovskiy territory, Ayano-Mayskiy Dist., Chouyka River near Nelkan village, 09.08.1978, *Kharkevich S. and Buch T., s.n.* (K).

SWEDEN: Örnsberg on Kuinekulle, 05.07.1841, *Lagerheim N. and C. s.n.* (K).

SWITZERLAND: Meiringen, 600 m, 06.1914, *Gamble J. S. s.n*.

UNITED KINGDOM: Dorset, Batecombe Down, 60 m, 21.07.1956, *Summerhayes, V. S. 2940* (K).

### 36. Herminium neotineoides

Taxon classificationPlantaeAsparagalesOrchidaceae

Ames & Schltr., Repert. Spec. Nov. Regni Veg. Beih.4: 42. 1919.

Peristylus neotineoides (Ames & Schltr.) K. Y. Lang, Acta Phytotax. Sin. 25: 453. 1987.
Monorchis neotineoides (Ames & Schltr.) O. Schwarz. Mitt. Thüring. Bot. Ges. 1: 95. 1949.

#### Type.

CHINA. Sichuan, Western Szechuan, 12000 ft, 1907, *Wilson E. H., 1768* (Holotype: AMES! [AMES 00100259]; Isotypes: AMES! [AMES00100261], K! [K000796955]).

#### Description.

Plant 20–38 cm tall. Tubers oblong-ovoid, 1–2 × 1–1.5 cm. Stem with 2–3 tubular sheaths and 2 leaves at base. Leaves subopposite, oblong-lanceolate, 4–15 × 0.8–3 cm, apex acute to acuminate. Inflorescence 25–28 cm, peduncle cylindrical, with 1 peduncle-scale; rachis 8–18 cm, densely many flowered; floral bracts lanceolate, 5–15 × 1–2 mm, longer than ovary, apex acuminate. Flowers green; pedicel and ovary 4–5 mm. Dorsal sepal elliptic, 2–2.5 × 1–1.5 mm, apex obtuse; lateral sepals spreading, elliptic, 2–2.5 × 1.5 mm, apex obtuse. Petals hooded with dorsal sepal, ovate-lanceolate, oblique, 2–2.5 × ca. 1 mm, concave, apex obtuse. Lip ovate, 2–3 × 2–2.5 mm, base concave, 3-lobed toward apex; disk with a subglobose callus; lateral lobes lanceolate, falcate, apex obtuse; mid-lobe ovate-lanceolate, slightly longer and broader than lateral lobes, apex obtuse; spur saccate; Column ca. 1.5 mm, viscidia ovate, rostellum triangular, 1 mm, stigma transversely oblong.

#### Flowering time.

July.

#### Habitat.

Terrestrial on grassy slopes and pastures between *Pinus* trees at elevations of 3100–4000 m.

#### Distribution.

China (Sichuan).

#### Specimens examined.

CHINA: **Sichuan**, Kangding county, 3150 m, 24.07.1963, *Guan K. & Wang W. et al. 340* (PE).

### 37. Herminium ophioglossoides

Taxon classificationPlantaeAsparagalesOrchidaceae

Schltr., Notes Roy. Bot. Gard. Edinburgh 5: 96. 1912.

Fig. 9N

Monorchis ophioglossoides (Schltr.) O. Schwarz, Mitt. Thüring. Bot. Ges. 1: 95. 1949.

#### Type.

CHINA. Yunnan, Lijiang, 10000 ft, 07.1906, *Forrest G., 2466* (Holotype: E! [E00188160]; Isotypes: K! [K000079034], P! [P00378738]).

#### Description.

Plant 6–26 cm tall. Tubers globose to ovoid, 10–30 × 5–15 mm. Stem with 2–3 tubular sheaths and 1–3 leaves at base. Leaves elliptic-lingulate, 2–10 × 0.5–3 cm, apex obtuse. Inflorescence 4–20 cm; peduncle cylindrical, ebractiate or rarely single foliaceous sterile bract, 0.5–3 cm; rachis 1–14 cm, laxly many flowered; floral bracts lanceolate, 2–5 mm, shorter than ovary, apex acuminate. Flowers yellowish green; pedicel and ovary arcaute, twisted, beaked at apex, 4–10 mm. Dorsal sepal oblong-lanceolate, 3.5–5 × 1–2 mm, apex obtuse; lateral sepals oblong-lanceolate, 3–5 × 1–2 mm, apex obtuse. Petals narrowly lanceolate-caudate, falcate, 5–7 × 0.6–0.9 mm, apex obtuse. Lip oblong, 5–7 × 1.5–2.5 mm, base deeply concave with 1 mm, globose spur, 3- lobed at or below middle; lateral lobes linear, 2–4 × ca. 0.5 mm at base; mid-lobe linear, 3–5 × ca. 0.4 mm, apex acute. Column ca. 1 mm; pollinia globose; caudicles indistinct or very short, viscidia involute, hornlike, rostellum triangular, with arm-like lobes, stigma transversely oblong, situated below the rostellum. Fruit oblong, 5–11 mm long.

#### Flowering time.

June–July.

#### Habitat.

Terrestrial on grassy slopes at elevations of 2100–3600 m.

#### Distribution.

China (Qinghai, Sichuan, Yunnan).

#### Specimens examined.

CHINA: **Qinghai**, Tongde county, Hebei town, Naxiuma, 3550 m, 27.07.1990, *Wu et al. 4978* (PE); **Sichuan**, Muli county, 3500 m, 16.06.1937, *Yu C. T., 6324* (PE); South Szechuan between Yenyuan Hsien and Hunbea, 2900 m, 12.06.1914, *Schneider C., 1465* (K); **Yunnan**, Lijiang County, near Jade Lake, 2800 m, 04.07.1996, *Luo, Y. B., 59* (K); Lijiang, east of Yulong mountain, 2900, 05.08.1981, *Beijing Hengduan Mountain Team,02693A* (PE); Lichiang Range, 3048 m, 06.1906, *Forrest G., 2466* (K); Zhongdian,3200 m, 01.06.1937, *Yu T. T.,11475* (PE); Zhongdian county, Tuguanchun, 2870 m, 20.07.1997, *Luo Y. B.,153* (9726) (K, PE); Hee-chan-men Pass, 14.06.1887, *Delavay J. M., s.n* (K).

### 38. Herminium oxysepalum

Taxon classificationPlantaeAsparagalesOrchidaceae

(K.Y. Lang) X.H. Jin, Schuit., Raskoti & L.Q. Huang, Cladistics 2015.

#### Basionym.

*Androcorys oxysepalus* K.Y. Lang, Guihaia 16: 106. 1996.

#### Type.

CHINA. Yunnan, Shangri-La, 3700 m, 10.08.1981, *Hengduan Mountain Team of Institute of Botany, 2917* (Holotype: PE!).

#### Description.

Plant 6–16 cm tall. Tuber globose, ca. 5 × 5 mm in diam. Stem slender, with 2 tubular sheaths and 1 leaf at base. Leaf elliptic or oblong, 1.5–1.7 × 0.8–0.9 cm, apex obtuse. Inflorescence 5–12 cm, rachis 1.8–4 cm, 6–7-flowered; floral bracts ovate, ca. 0.1 mm, shorter than ovary, apex acute. Flowers green, ovary and pedicel fusiform, slightly beaked, 3–3.3 mm. Dorsal sepal broadly ovate, concave, ca. 1–1.5 × 1–1.6 mm, margin denticulate, apex acute; lateral sepals spreading, oblong-lanceolate, concave, ca. 2.5–3 × 1–1.3 mm, margin denticulate, apex acuminate. Petals oblong-lanceolate, oblique, concave, ca. 1.5–2 × 0.8–1 mm, apex obtuse. Lip deflexed, lingulate-lanceolate, ca. 1.5–2 mm, fleshy, base dilated, apex obtuse. Column ca. 0.5 mm, anther with 2 divergent, hooded locules and broad connective; pollinia 2, clavate, attached to a viscidium by short caudicle; rostellum triangular; stigma 2 pulvinate, attached to base of rostellum. Fruit fusiform, 5–7 mm long.

#### Flowering time.

August.

#### Habitat.

Terrestrial in *Abies* forest at an elevation of 3700 m.

#### Distribution.

China (Yunnan).

#### Specimens examined.

CHINA: **Yunnan**, Zhongdian, 3700 m, 10.08.1981, *Hengduan Mountain Team of Institute of Botany, 2917* (PE); **Tibet**, Yadong county, 25.08.2013, *FLPH Tibet Expedition Team, 13–2182* (PE).

### 39. Herminium pugioniforme

Taxon classificationPlantaeAsparagalesOrchidaceae

Lindl. ex Hook.f., Fl. Brit. India 6: 130. 1890.

Fig. 5I

Herminium nivale Schltr., Acta Horti Gothob. 1: 134. 1924. Type: CHINA. Sichuan, Dongrergo, *Smith H., 3631* (Holotype: UPS!).
Monorchis pugioniformis (Lindl. ex Hook.f.) O. Schwarz, Mitt. Thüring. Bot. Ges. 1: 96. 1949.
Androcorys pugioniformis (Lindl. ex Hook.f.) K. Y. Lang, Guihaia 16: 105. 1996.

#### Type.

INDIA. **Uttarakhand**, Garhwal, Dudu Glacier under Srikanta, 14000–15000 ft, 10.08.21883, *Duthie J. F., 517* (Syntype: K! [K000387533]); **Sikkim**, Samdong, 16000 ft, 11.09.1849, *Hooker J. D., s.n.* (Syntype: K! [K000387531], K-LINDL!); **Kashmir**, Kilan Marg, 12000 ft, 08.1877, *Aitchison, 106* (Syntype: K! [K000387532]).

#### Description.

Plant 5–20 cm tall. Tuber globose, 5–8 × 4–8 mm. Stem with 1 or 2 tubular sheaths and 1 leaf at base. Leaf oblanceolate, 2–4 × 0.4–1.2 cm, apex obtuse. Inflorescence 3–10 cm, rachis 1–4 cm, 3–20-flowered; floral bracts ovate, much reduced, shorter than ovary, apex acute. Flowers green; ovary and pedicel 4–5 mm, fusiform, apex beaked, 3–5 mm. Dorsal sepal broadly elliptic-orbicular, concave, 1.5–2.5 × 1–1.2 mm, apex obtuse; lateral sepals obovate to elliptic, oblique 1.7–2.2 × 1–1.2 mm, apex obtuse. Petals obovate, oblique, concave, 1.3–1.5 × 0.5–0.8 mm, apex obtuse. Lip narrowly lingulate-oblong, 1.5–2.0 mm long, base rather abruptly dilated, 0.6–1.1 mm wide, with two basal concavities separated by a longitudinal callus ridge, apex obtuse. Column 0.5–1 mm, anther with 2 divergent locules hooded with ca. 0.6 mm broad connective; pollinia 2, clavate, attached to ellipsoid viscidium by short caudicle; rostellum triangular; stigma gibbous, attached to base of rostellum. Fruit fusiform, 3–4 mm long.

#### Flowering time.

July–September.

#### Habitat.

Terrestrial in *Abies* forests, alpine grasslands, meadows and valleys at elevations of 2700–5200(–5500) m.

#### Distribution.

Bhutan, China, India, Nepal.

#### Note.

Pridgeon et al. (2001: 257) stated that this species may occur up to 5500 m, but we have not seen specimens collected above 5200 m.

#### Specimens examined.

BHUTAN: Gasa, Baiteng, 5040 m, 25.08.2000, *Miehe G. and Miehe S., 00-329-04* (K); Gasa, Upper mangde Chu near Kitchisabo, 5190 m, 11.09.2000, *Miehe G. and S., 00-368-17* (K); Tangey, lower Tsorim Chu, 4670 m, 14.09.2000, *Miehe G. and Miehe S., 00-385-17* (K); Paro, Tso Phu Valley, 4610 m, 08.07.2000, *Miehe G. and Miehe S., 00-175-24* (K).

CHINA: **Sichuan**, Songpan county, Huanglongsi, 3450 m, 30.07.2002, *Luo Y.B., 853* (PE); **Tibet**, Bomi county, Zhamo Highway, 4200 m, 27.07.2010, *Southern Tibet Expedition, STET1287* (PE); Dagze county, 5200 m, 02.09.1965, *Zhang Y.T. and Lang K.Y., 2475* (PE); Damxung, Nyainqentanglha Shan, 4880 m, 11.08.1989, *Dickoré B., 3753* (K); Lhasa, 5000 m, 19.08.1965, *Zhang Y.T. and Lang K.Y., 1938* (PE); Lhasa, Ximala Hill, mountain, 4900 m, 10.08.1965, *Zhang Y.T., Lang K.Y., 2114* (PE); Medog county, 2664 m, 08.09.2009, *Southeast Tibet Expedition, SET-ET 1362* (PE); Melong Gompa, 4900–5200 m, 02.07.1932, *Gould B. J., 2236* (K); Zayu county, Chawalong from meigu to Ridong, 4500 m, 17.07.2010, *Southern Tibet Expedition, STET0655* (PE); **Yunnan**, Zhongdian, 3880 m, 11.08.1981, *Beijing Hengduan Mountain Team, 3018* (PE).

INDIA: **Kashmir**, Mt. Kolahoi, 4250 m, *Stewart R. R., 9390* (K).

NEPAL. **Rasuwa**, Gosaithan, 4000 m, 21.07.2008, *Raskoti B. B., 20149* (KATH).

### 40. Herminium pusillum

Taxon classificationPlantaeAsparagalesOrchidaceae

Ohwi & Fukuy., Bot. Mag. (Tokyo). 48 (571): 430. 1934.

Androcorys pusillus (Ohwi & Fukuy.) Masam., Hokuriku J. Bot. 12: 88. 1963.
Androcorys japonensis Maek., J. Jap. Bot. 12: 96. 1936. Type: JAPAN. Hondo, Shinano Prov., Mt. Yuo, 18.08.1902, *Yabe Y. s.n.* (Holotype: Herb. Univ. Imp. Tokyo).

#### Type.

CHINA. Taiwan. Taichu (Mt. Niitakayama), 07.1933, *Ohwi J.*, (TAI!).

#### Description.

Plant 10–22 cm tall. Tuber globose, 5–7 mm in diam. Stem slender, with 1–3 tubular sheaths and 1 leaf at base. Leaf ovate, 2–4.5 × 1–2.2 cm, apex obtuse. Inflorescence 5–10 cm, rachis ca. 2.5 cm, 8–13-flowered; floral bracts ovate, ca. 1.5 mm, shorter than ovary. Flowers green; ovary and pedicel, fusiform, slightly beaked, 3–4 mm. Dorsal sepal erect, broadly ovate, concave, 1–1.2 × ca. 1 mm, margin irregularly ciliated, apex obtuse; lateral sepals spreading, oblong, 2–3 × 0.7–1 mm, margin ciliated, apex obtuse. Petals oblong, concave, 1.2–1.5 × ca. 1 mm, base cuneate, apex obtuse. Lip lingulate, 2–2.2 × ca. 0.7 mm, fleshy, base dilated, apex obtuse. Column short, anther with 2 divergent, hooded locules and broad connective; pollinia 2, clavate, attached to a viscidium by short caudicle; rostellum triangular; stigmalobes 2, pulvinate, attached to base of rostellum.

#### Flowering time.

July.

#### Habitat.

Terrestrial in forest margins and alpine valleys at elevations of 2500–3500 m.

#### Distribution.

China, Korea, Japan.

#### Specimens examined.

CHINA: **TAIWAN**, Hualien county, Nanhuchih shelter, 3100–3400 m, 07.07.2005, *Chuan T., 215* (TAIF).

### 41. Herminium pygmaeum

Taxon classificationPlantaeAsparagalesOrchidaceae

Renz, Edinb. J. of Bot. 56(1):106. 2001.

Figs 6C , 7C , 8C , 9C , 11C

#### Type.

BHUTAN. Thimphu district, below Darkey Pang Tso, north of Paro, 3960 m, 04.08.1991, *Noltie H. J., 105* (Holotype: E! [E00008013]).

#### Description.

Plant 2–16 cm tall. Tuber globose, 3–4 × 3–4 mm. Stem with a sheath and 2–3 basal leaves. Leaves linear-oblong, 1.5–3.5 × 0.5–0.15 cm, apex subacute. Inflorescence 2–10 cm, peduncle cylindrical with 1.5–2.5 × 0.5–2 mm, lanceolate, acute peduncle-scales, rachis glabrous, 2–4.5 mm long subdensely 3–5-flowered; floral bracts minute, lanceolate, acute, 0.5–2 × 0.5–0.6 mm. Flowers secund, 3–5 mm long, sepals green, petals yellowish, pedicel and ovary swollen, 3–5 × 1–1.3 mm, apex beaked and strongly hooked. Dorsal sepal ovate, concave, 3–4 × 1.5 mm, apex obtuse, lateral sepals ovate-lancelate, oblique, apex obtuse, 3–4 × 1.5–2 mm. Petals trowel-shaped, oblique, fleshy and narrowed towards apex, apex subacute, 3–3.5 × 1–1.5 mm, lip ovate, base concave, spurless, 3–4 × 2–2.5 mm, 3-lobed above middle, lateral lobes triangular, obtuse; mid-lobe narrowly triangular, apex acute, 1 mm long. Column 0.7 mm tall, pollinia globose; caudicles indistinct, viscidia involute, horn-like, rostellum triangular, stigma transversely oblong, situated below the rostellum. Fruit oblong, 4–5 mm long.

#### Flowering time.

July–August.

#### Habitat.

Terrestrial on wet cliff ledge at elevations of 3900–4000 m.

#### Distribution.

Bhutan, China.

#### Specimens examined.

CHINA: **Tibet**, Yadong county, Xiakangbu to Shangkangbu, Road 714, 71 km, 3965 m, 24.08. 2013, *FLPH Expidition Team*, 13-2115 (PE).

### 42. Herminium quinquelobum

Taxon classificationPlantaeAsparagalesOrchidaceae

King & Pantl., J. Asiat. Soc. Bengal, Nat. Hist. 65: 130. 1896.

Figs 5H , 6O , 7O , 8O , 9O

Monorchis quinqueloba (King & Pantl.) O. Schwarz, Mitt. Thüring. Bot. Ges. 1: 96.1949.

#### Type.

INDIA. Sikkim, Tendong, 7000 ft, 08.1894, *Pantling R., 339* (Holotype CAL; Isotype: K! [K000079023]).

#### Description.

Plant 15–30 cm tall. Tubers oblong to subglobose, 15–20 × 5–15 mm. Stem with 2 tubular sheaths at base and 2–3 leaves. Leaves subopposite, linear-lanceolate, 10–17 × 1–2 cm, apex acute. Inflorescence 10–20 cm; peduncle with a foliaceous linear-lanceolate peduncle-scale; rachis 10–15 cm, many flowered; floral bracts lanceolate, 3–8 mm, equal or longer thna ovary, apex acuminate. Flowers greenish white; pedicel and ovary straight, apex beaked, 5–10 mm. Dorsal sepal oblong-lanceolate, 2–3.5 × 0.5–1 mm, apex obtuse; lateral sepals oblong, 2–3 × 0.6–1 mm, apex subacute. Petals linear, 1.6–3.5 × ca. 0.3–0.5 mm, apex acute. Lip oblong, 5-lobed, 2–3.5 × 0.7–1 mm, base dilated and concave; basal lobes triangular, lateral lobes linear, 1 × 0.5 mm, apex acute; mid-lobe oblong-triangular, 1.5 × 0.5 mm, apex acuminate. Column ca. 7 mm, stigma transversely oblong, situated underneath the rostellum; auricles oblong, rostellum triangular; pollinia obovoid; caudicles short, viscidia disklike. Fruit oblong, 1 cm long.

#### Flowering time.

August–September.

#### Habitat.

Terrestrial on moist slopes or epiphytic in evergreen broad-leaved forests at elevations of 2100–3000 m.

#### Distribution.

Bhutan, China, India, Nepal.

#### Specimen examined.

CHINA: **Yunnan**, Gongshan county, 2200 m, 03.09.1982, *Qinghai-Tibet Team, 9908* (PE).

INDIA: **Sikkim**, Tendong, 2134 m, 08.1894, *Pantling R., 339* (K).

NEPAL: **Janakpur**, Dolakha district, Jiri, 2950 m, 21.09.1964, *Benerji M. L., Shrestha T. B.* and *Upadhyay A. V., 2877* (KATH); **Koshi**, Dhankuta district Chitrya, 2240 m, 11.09.1977, *Pradhan P. and Shrestha N.,89* (KATH); **Mechi**, Ilam district Hile, 2390 m, 06.09.1977, *Pradhan P., Rajbhandari K. R*. *and Niraula R., 263* (KATH); between Tala (2050 m) and Tale Bisauna (2750 m), 10.09.1970, *Kanai H., Chuma C. and Nagano T., s.n.* (K).

### 43. Herminium singulum

Taxon classificationPlantaeAsparagalesOrchidaceae

Tang & F. T. Wang, Bull. Fan Mem. Inst. Biol. Bot. 10: 35. 1940.

#### Type.

CHINA. Yunnan, Salween-Chiukiang, east of Wang-tzang, 2800 m, *Yu T.T., 20231A* (Holotype: PE! [PE01432256]).

#### Description.

Plant 10–30 cm tall. Tubers subglobose, 8–10 mm. Stem with one or two tubular sheaths and 1 leaf at base. Leaf elliptic, 2–5 × 1–2 cm, apex obtuse. Inflorescence 6–25 cm; peduncle cylindrical, with 1 or 2 lanceolate peduncle-scales, 1–1.5 cm; rachis 2–10 cm, sublaxly 4 or many flowered; floral bracts lanceolate, 5 × 2 mm, shorter or longer than to ovary, apex acuminate. Flowers pedicel and ovary arcuate, apex beaked, 6–10 mm. Dorsal sepal ovate-lanceolate, 2–3 × 1–1.3 mm, concave, apex acute; lateral sepals reflexed, obliquely ovate, 2.5–3 × 0.8–1 mm, apex acute. Petals ovate, oblique, ca. 1.8–2.2 × 1–1.5 mm, apex obtuse. Lip lanceolate, 3.2–5 × 1–1.3 mm, entire, base dilated and spurred, apex obtuse; spur cylindrical shorter than ovary. Column 1.5 mm; caudicles short, rostellum triangular, stigma transversely oblong, situated below rostellum.

#### Flowering time.

August–September.

#### Habitat.

Terrestrial in forest margins at elevations of 2600–2800 m.

#### Distribution.

China (Sichuan, Yunnan).

#### Specimens examined.

CHINA: **Yunnan**, Gongshan county, Dulong, Gaolingshan, 1979, *Liu L.H., et al. 08–14* (PE); Salween-Chiukiang, 2800 m, 03.09.1938, *Yu T. T., 20231A* (PE).

### 44. Herminium souliei

Taxon classificationPlantaeAsparagalesOrchidaceae

(Finet) Rolfe, J. Linn. Soc., Bot. 36: 51. 1903.

Herminium souliei Schltr., Repert. Spec. Nov. Regni Veg. 9: 22. 1910, nom. illeg. (non (Finet) Rolfe). Type: CHINA. Tibet, Kiala Dist., Togolo, near Tat-sien-lou, 1898, *Soulié J. A., s.n.* (Holotype: B, lost; Isotype: [presumably *Soulié 407*] P!).
H. souliei var. lichiangense W. W. Sm., Notes Roy. Bot. Gard. Edinburgh 8: 337. 1915. TYPE: CHINA. Yunnan, eastern flank of the Lichiang Range, 11000 ft, 08.1910, *Forrest G., 6399* (not found).
H. limprichtii Schltr., Repert. Spec. Nov. Regni Veg. Beih.4: 42. 1919. TYPE: CHINA. Yunnan, Talifu, 3500 m, 08.1913, *Limpricht H. W., 1003* (Holotype: B, lost).
Monorchis limprichtii (Schltr.) O. Schwarz, Mitt. Thüring. Bot. Ges. 1: 95. 1949.
M. souliei (Finet) O. Schwarz, Mitt. Thüring. Bot. Ges. 1: 95. 1949.

#### Basionym.

Herminium angustifolium var. souliei Finet, Rev. Gén. Bot. 13: 518. 1901.

#### Type.

CHINA. Tibet, Kiala Dist., Togolo, 18.08.1893, *Soulié J. A., 407* (Holotype: P! [P00378650]; Isotypes: K! [K000079033], P! [P00378650]).

#### Description.

Plant 8–38 cm tall. Tubers oblong-ellipsoid, 8–25 × 5–15 mm. Stem with 2 or 3 tubular sheaths at base and 2–4 leaves. Leaves well-spaced, linear to lanceolate, 4–20 × 0.5–1.5 cm, apex acute. Inflorescence 5–25 cm; peduncle cylindrical, with peduncle-scales 0.5–2.5 cm; rachis 2.5–20 cm, densely many flowered; floral bracts ovate-lanceolate, 2–5 mm, equal, shorter or longer than ovary, apex acuminate. Flowers pale green; pedicel and ovary arcuate, 5–10 mm, shortly beaked. Dorsal sepal ovate, concave, 2–3 × 1.5–2 mm, apex obtuse; lateral sepals ovate, oblique, 2.9–3.5 × 1.2–2.5 mm, apex obtuse. Petals connivent with dorsal sepal forming a hood, linear, 2–3 × 0.4–0.6 mm, apex obtuse. Lip oblong, 2.6–4.5 × 0.7–1.2 mm, 3-lobed; lateral lobes linear-falcate, 0.5 × 0.3 mm; mid-lobe oblong, 0.6 × 0.3 mm, apex obtuse. Column 1 mm; pollinia ovoid; caudicles short, viscidia ovoid, rostellum triangular, stigma transversely oblong, situated below the rostellum. Capsule oblong, 1 cm.

#### Flowering time.

July–August.

#### Habitat.

Terrestrial in broad-leaved forests margins or on grassy slopes at elevations of 1400–4200 m.

#### Distribution.

China, Nepal.

#### Note.

Morphologically, *Herminium souliei* is similar to *Herminium lanceum*, but *H. souliei* is shorter in stature and the lip is flatter, which gives the flowers a more spidery appearance.

#### Specimens examined.

CHINA: **Sichuan**, Muli county, 2800 m, 03.08.1937, *Yu C. T., 7564* (PE); Yanyuan county, Yuanbao area, Dalin town, 3200 m, 20.07.1983 (PE); *Qinghai-Tibet Team, 12041A* (PE); Xiangcheng county, 3900 m, 12.08.1983, *Qinghai-Tibet Team, 4780* (PE); Daofu county, 3600 m, 26.08.2001, *Luo Y.B., 687* (PE); **Yunnan**, eastern slopes of Likiang Snow Range, 1922, *Rock J. F., 5851* (K); Lijiang, 3500 m, 03.08.1981, *Beijing Hengduan Mountain Team,02550* (PE); Qiaojia county, 1400 m, 15.09.1932, *Tsai H. T., 52045* (PE); Shangri-La, 2900 m, 17.09.1962, *Zhongdian Team, 2116* (PE); Shangri-La, 3250 m, 09.08.1981, *Tian et al., 842* (PE).

NEPAL: **Koshi**, Dhankutta district, Chitrye, 2400 m, 29.08.2007, *Raskoti B. B.,02007* (KATH).

### 45. Herminium suave

Taxon classificationPlantaeAsparagalesOrchidaceae

Tang & F. T. Wang, Bull. Fan Mem. Inst. Biol. 7: 131. 1936.

Peristylus forrestii (Schltr.) K. Y. Lang, Acta Phytotax. Sin. 25: 454. 1987. Not Herminium forrestii Schltr.

#### Replaced name.

*Habenaria forrestii* Schltr., Notes Roy. Bot. Gard. Edinburgh 5: 101. 1912. Type: CHINA. Yunnan, eastern flank of the Lichiang Range, 11000–12000 ft, 15.09.1906, *Forrest G., 2875* (Holotype: E! [E00381990]; Isotype: P! [P00378752]).

#### Description.

Plant slender, 17–33 cm tall. Tubers oblong, 1–2.5 × 0.5–1 cm. Stem with upto 3 tubular sheaths and 2–3 leaves at base. Leaves linear, 1.5–8 × 0.1–0.4 cm, apex acuminate. Inflorescence 12–30 cm; peduncle cylindrical, peduncle-scales 1–3, lanceolate, 3–10 mm; rachis 5.5–13 cm, laxly to sub-densely many flowered; floral bracts ovate-lanceolate, 3–4 mm, much shorter than ovary, apex acuminate. Flowers green, ovary and pedicel 5–8 mm. Dorsal sepal broadly ovate, ca. 2–2.5 × 1.5–2 mm, apex obtuse; lateral sepals oblong, falcate, reflexed, ca. 2–2.5 × 1–1.5 mm, apex obtuse. Petals ovate, oblique, ca. 2–3 × 1–1.5 mm, apex obtuse. Lip lingulate, ca. 3 mm, base concave, entire, apex obtuse; spur pendulous, curving forward, cylindrical, 5–8 mm, ca. equal to or longer than ovary, slightly dilated toward apex. Column ca. 1 mm, pollinia ovoid; caudicles short; viscidia ovate, rostellum triangular, armed, stigma transversely oblong, drawn out at base of lip.

#### Flowering time.

August–September.

#### Habitat.

Terrestrial on grassy slopes at elevations of 1700–3900 m.

#### Distribution.

China (Sichuan, Yunnan).

#### Specimens examined.

CHINA: **Sichuan**, Muli, 2600 m, 12.08.1937, *Yu T.T., 7674* (PE); **Yunnan**, without data, *Maire E. E., 38* (K).

### 46. Herminium tangianum

Taxon classificationPlantaeAsparagalesOrchidaceae

(S. Y. Hu) K. Y. Lang, Acta Phytotax. Sin. 25: 458. 1987.

Herminium latifolium Homotypic synonyms: Gagnep., Bull. Mus. Natl. Hist. Nat., sér. 2, 3: 325. 1931, not (A. Rich.) Lindl. 1832 (= Benthamia chlorantha (Spreng.) Garay & G.A. Romero).
P. ecalcaratus Tang & F. T. Wang, Acta Phytotax. Sin.1: 64. 1951, not Finet 1901.

#### Basionym.

*Peristylus tangianus* S. Y. Hu, Quart. J. Taiwan Mus. 27: 461. 1974.

#### Type.

CHINA. Yunnan, 20.08.1906, *Ducloux F., 3971* (Holotype P! [P00378693]).

#### Description.

Plant 20–30 cm tall. Tubers subglobose to oblong-ellipsoid, 10–20 mm. Stem with 3–4 tubular sheaths at base and 2–3 subbasal leaves. Leaves orbicular or elliptic, 2.1–5 × 2–2.6 cm, apex subacute to acuminate. Peduncle cylindrical, with several triangular lanceolate peduncle-scales, rachis 4–10 cm, subdensely many-flowered; floral bracts lanceolate, 6–7 mm, slightly shorter than ovary, apex acuminate. Flowers white; pedicel and ovary twisted, beaked, ca. 8 mm. Dorsal sepal ovate, ca. 2.5 × 1.6 mm, apex obtuse; lateral sepals oblanceolate, ca. 2.6 × 1 mm, apex obtuse. Petals ovate, oblique, 1.2–2.4 × ca. 1.2 mm, apex obtuse. Lip decurved, ovate-pandurate, ca. 2.5 × 1.4 mm, somewhat fleshy, base dilated and shallowly concave, contracted near middle, margin entire, apex dilated and obtuse. Column short; viscidia elliptic, ca. 0.8 mm.

#### Flowering time.

August.

#### Distribution.

China (Yunnan).

#### Note.

Our description is based on Chen et al. (2009).

### 47. Herminium tibeticum

Taxon classificationPlantaeAsparagalesOrchidaceae

X.H. Jin, Schuit. & Raskoti sp. nov.

urn:lsid:ipni.org:names:77161963-1

Fig. 15

#### Type.

CHINA. Tibet, Medog, 52 km region of the road from Bomi to Motuo, 3600–3700 m, 28.08.2012, *Jin X.H., Jin W.T. and Xu S.Z., 13185* (PE).

**Figure 15.**
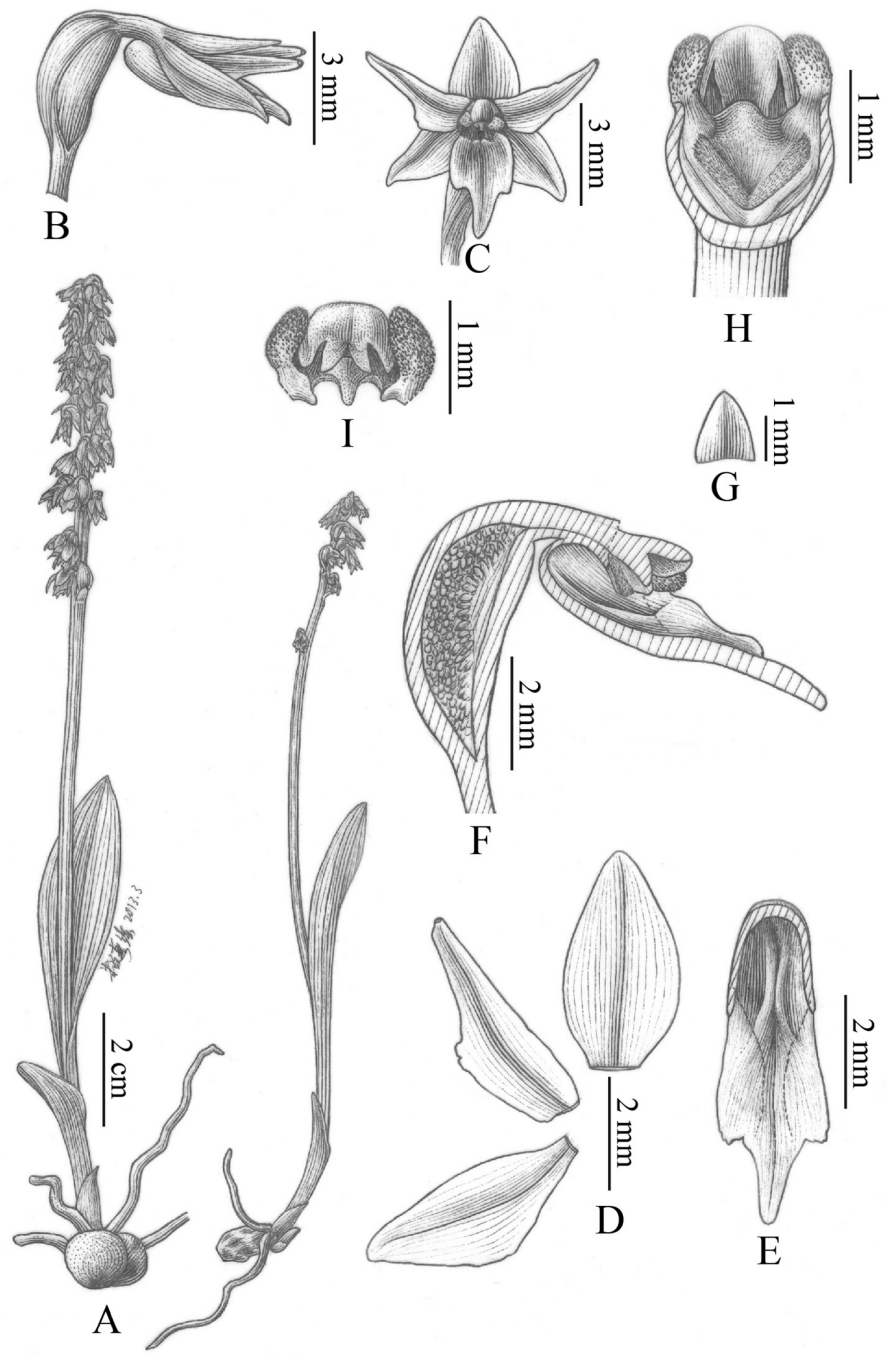
*Herminium tibeticum*. **A** Flowering plant **B** Flower (side view) **C** Flower spread (front view) **D** Dorsal sepal, lateral sepal and petal **E** Lip **F** Section of ovary with column **G** Floral bract **H** Column with stigma (front view) **I** Anther cap, auricles and viscidium.

#### Diagnosis.

*Herminium tibeticum* is similar to *Herminium pygmaeum* and *H. josephi*, but differs from the former in having strongly falcate petals and a lip with a callus; from the latter in having a 3-lobed and cuneate lip.

#### Description.

Plant 13–16 cm tall. Tubers subglobose, 4–8 × 4–6 mm, with slender roots. Stem with tubular sheaths at base, and often 1 or rarely 2-leaved. Leaves cauline, lanceolate, 4.0–5.0 × 0.8–1.0 cm, apex acute, base sheathing. Peduncle 7–8 cm, slender, without peduncle-scales; rachis 2.5–5.5 cm, with five to several subsecund flowers; floral bracts ovate,1–2 × 0.5–1.0 mm, about equal to the pedicel, apex acute. Flowers drooping, greenish, 4–5 mm across, ovary fusiform, shortly pedicellate, slightly arcuate, 4–7 mm long, ridged, apex strongly beaked. Dorsal sepal ovate, 3.0 × 1.9 mm, 1-veined, apex sub-obtuse; lateral sepals spreading, ovate, oblique, 3.0 × 1.3 mm, apex obtuse. Petals lanceolate, falcate, 3.0–5.0× 1.0–1.5 mm, 1-veined, middle portion crenulate, apex acute. Lip cuneate, apiculate, 5.5–6.0 × 1.5–2.5 mm, base saccate, concave, with two longitudinal 1.9 mm long calli, 3-nerved, shallowly 3-lobed; lateral lobes triangular, smaller than the midlobe, 1.0 × 0.4 mm, crenulate, apex obtuse; midlobe narrowly triangular,1.2 × 1.0 mm, apex sub-obtuse. Column ca. 1.4 mm long; stigma transverse, ca. 2.8 mm long, rostellum 0.5 mm, three lobed, mid-lobe larger than the lateral lobes; auricle ca. 0.6 mm long; pollinia 1.2 mm, globose; viscidia globose.

#### Flowering time.

July.

#### Distribution.

Endemic to China.

#### Habitat.

Terrestrial in grassland on open slopes in evergreen forest at elevations of 3600–3700 m.

#### Etymology.

Named for Tibet, origin of type.

#### Notes.

*Herminium tibeticum* is most similar to *Herminium pygmaeum* Renz in petal and lip shape; it differs from the latter in having strongly falcate petals, and a lip with two parallel longitudinal calli at the base.

### 48. Herminium wangianum

Taxon classificationPlantaeAsparagalesOrchidaceae

X.H. Jin, Schuit., Raskoti & L.Q. Huang, Cladistics 2015.

Herminium spirale (Not) (A. Rich.) Rchb. f., Bonplandia (Hannover) 3: 213. 1855 (= Benthamia africana (Lindl.) Hermans).

#### Replaced name.

*Androcorys spiralis* Tang & F. T. Wang, Bull. Fan Mem. Inst. Biol. Bot. 10: 38. 1940.

#### Type.

CHINA. Tibet, Chayu, 3500 m, 09.1935, *Wang C.W., 66285* (PE!).

#### Description.

Plant 5–12 cm tall. Tuber globose, 5–8 mm in diam. Stem slender, with 2 tubular sheaths and 1 leaf at base. Leaf oblong, 2–2.5 × 0.8–1 cm, apex obtuse. Inflorescence 4–5 cm, ebracteate, rachis 2–2.5 cm, 3–8-flowered; floral bracts spirally twisted, linear, shorter than ovary, apex acuminate. Flowers green; ovary and pedicel, fusiform, apex shortly beaked, 3–7 mm. Dorsal sepal broadly ovate-elliptic, concave, 1.5–2 × 1.3–1.8 mm, apex apiculate; lateral sepals spreading, oblong-lanceolate, 2–2.5 × 1–1.2 mm, apex obtuse. Petals oblong, oblique, 1.3–2 × 0.5–0.7 mm, apex obtuse. Lip linear-lingulate, 1.8–2.1 mm, fleshy, base dilated and ca. 0.8 mm wide, apex obtuse. Column short, anther with 2 divergent, hooded locules and broad connective; pollinia 2, clavate, attached to a viscidium by short caudicle; rostellum triangular; stigma 2 pulvinate, attached to base of rostellum.

#### Flowering time.

September.

#### Habitat.

Forested slopes at elevations of 2800–4800 m.

#### Distribution.

China (Sichuan, Xizang, Yunnan).

#### Specimen examined.

CHINA: **Qinghai**, Zadoi county, 4800 m, 25.07.1965, *Liu S. W., 217* (PE); **Sichuan**, Muli county, 2800 m, 15.08.1937, *Yu T. T., 7736* (PE); **Tibet**, Zayu county, 3500 m, 09.1935, *Wang C. W., 66285* (PE); Zayu county, 2800 m, 08.1938, *Wang Q. W., 65222* (PE); Zayu county, Chawalong, 3200 m, 08.1935, *Wang C. W., 65782* (PE); Nyalam county, 4600 m, 20.08.2010, *PE-Tibet Team, 01640* (PE).

### 49. Herminium yunnanense

Taxon classificationPlantaeAsparagalesOrchidaceae

Rolfe, Notes Roy. Bot. Gard. Edinburgh 8: 24. 1913.

Monorchis yunnanensis (Rolfe) O. Schwarz, Mitt. Thüring. Bot. Ges. 1: 96. 1949.

#### Type.

CHINA. Yunnan, Tsan-Shan Range, near Yang Bi Pass, *Forrest G., 907* (Holotype: E! [E00188161]).

#### Description.

Plant 15–27 cm tall. Tubers ovoid-oblong, 5–20 × 4–8 mm. Stem with 2 tubular sheaths and 1 leaf at base. Leaf linear-oblong, 8–14 × 0.5–1 cm, apex acute. Inflorescence 14–22 cm; peduncle cylindrical, with few lanceolate peduncle-scales; rachis 5–9 cm, densely many-flowered; floral bracts ovate, 3–5 mm, shorter than ovary, apex acuminate. Flowers yellowish green; pedicel and ovary, twisted, beaked at apex, 3–6 mm. Dorsal sepal broadly ovate, concave, 1.5–2 × 1–1.6 mm, apex obtuse; lateral sepals ovate, slightly oblique, 1.5–2.3 × 1–1.5 mm, apex obtuse. Petals hooded with dorsal sepal, concave, ovate, 1–1.5 × 1–1.3 mm, apex subacute. Lip oblong, 2–2.5 × 1–2 mm, concave, apex 3-lobed; lateral lobes triangular, 0.5 × 0.3 mm, apex acute; mid-lobe triangular, 0.7 × 0.7 mm, apex obtuse. Column ca. 1 mm; pollinia globose; caudicles short, viscidia ovoid; rostellum clavate, stigma transversely oblong, situated below the rostellum. Fruit oblong, 5–7 mm long.

#### Flowering time.

August–September.

#### Habitat.

Terrestrial on grassy slopes at elevations of 2200–3300 m.

#### Distribution.

China (Yunnan).

#### Specimens examined.

CHINA: **Yunnan**, Fugong county, 19.07.2013, *Jin X. H, Wang L. S., Wang Q. et al. ST0599* (PE); Chuxiong city, 2200 m, 23.09.1939, *Li M. K.,0170* (PE); Yangbi county, 2673 m, 24.07.2006, *Jin X. H., 8273* (PE).

### Excluded species

***Herminium gongganum* Ormerod, Taiwania 58: 27 (2013).**

**Note.** Judging from the protologue, an image of the holotype, and our own field observations of similar plants (which had fleshy rootstocks, lacking a tuber), this taxon belongs to the genus *Platanthera*. Efimov (2016) transferred this species to *Platanthera*.

***Herminium orbiculare* Hook.f., Fl. Brit. India 6: 129 (1890).**

**Note.** Based on an examination of living and type material as well as a phylogenetic analysis using DNA data (Jin et al., unpublished) it is apparent that this species belongs in *Platanthera*, necessitating the following new combination.

#### Platanthera orbicularis

Taxon classificationPlantaeAsparagalesOrchidaceae

(Hook.f.) X.H. Jin, Schuit. & Raskoti comb. nov.

urn:lsid:ipni.org:names:77161964-1

##### Basionym.

*Herminium orbiculare* Hook.f., *Fl. Brit. India* 6: 129 (1890).

##### Type.

CHINA. Xizang, Chumbi Valley, Rungboo (Kungphu), *King’s Collector s.n.* (Holotype: K! [K000079022]).

## Supplementary Material

XML Treatment for HERMINIUM

XML Treatment for Herminium sect. Herminium

XML Treatment for Herminium sect. Thisbe X.H. Jin, Schuit. & Raskoti

XML Treatment for Herminium sect. Androcorys

XML Treatment for Herminium sect. Bhutanthera

XML Treatment for Herminium sect. Cybele X.H. Jin, Schuit. & Raskoti,

XML Treatment for Herminium sect. Pseudoplatanthera X.H. Jin, Schuit. & Raskoti

XML Treatment for Herminium alaschanicum

XML Treatment for Herminium albomarginatum

XML Treatment for Herminium albosanguineum

XML Treatment for Herminium albovirens

XML Treatment for Herminium biporosum

XML Treatment for Herminium bulleyi

XML Treatment for Herminium chloranthum

XML Treatment for Herminium clavigerum

XML Treatment for Herminium coeloceras

XML Treatment for Herminium coiloglossum

XML Treatment for Herminium ecalcaratum

XML Treatment for Herminium edgeworthii

XML Treatment for Herminium elisabethae

XML Treatment for Herminium fallax

XML Treatment for Herminium fimbriatum

XML Treatment for Herminium forceps

XML Treatment for Herminium glossophyllum

XML Treatment for Herminium gracile

XML Treatment for Herminium handelii

XML Treatment for Herminium himalayanum

XML Treatment for Herminium hongdeyuanii

XML Treatment for Herminium humidicola

XML Treatment for Herminium jaffreyanum

XML Treatment for Herminium josephi

XML Treatment for Herminium kalimpongense

XML Treatment for Herminium kamengense

XML Treatment for Herminium kumaunense

XML Treatment for Herminium lanceum

XML Treatment for Herminium latilabre

XML Treatment for Herminium longilobatum

XML Treatment for Herminium mackinnonii

XML Treatment for Herminium macrophyllum

XML Treatment for Herminium mannii

XML Treatment for Herminium monophyllum

XML Treatment for Herminium monorchis

XML Treatment for Herminium neotineoides

XML Treatment for Herminium ophioglossoides

XML Treatment for Herminium oxysepalum

XML Treatment for Herminium pugioniforme

XML Treatment for Herminium pusillum

XML Treatment for Herminium pygmaeum

XML Treatment for Herminium quinquelobum

XML Treatment for Herminium singulum

XML Treatment for Herminium souliei

XML Treatment for Herminium suave

XML Treatment for Herminium tangianum

XML Treatment for Herminium tibeticum

XML Treatment for Herminium wangianum

XML Treatment for Herminium yunnanense

XML Treatment for Platanthera orbicularis

## References

[B1] Agrawala D Chowdhery H Chaudhury S (2010) New combinations in Indian Orchidaceae. Kew Bulletin 65: 105–106. https://doi.org/10.1007/s12225-010-9179-2

[B2] Averyanov L Averyanova AL (2003) Updated Checklist of the Orchids of Vietnam. Vietnam National University Publishing House, Hanoi, 101 pp.

[B3] Averyanov L (2010) The orchids of Vietnam illustrated survey part 2 subfamily Orchidoideae. Turczaninowia 13(2): 5–98.

[B4] Boufford DE Ohashi H Huang TC Hsieh CF Tsai JL Yang KC Peng CI Kuoh CS Hsiao A (2003) A checklist of the vascular plants of Taiwan. Flora of Taiwan 6: 15–139.

[B5] Chen SC Lang KY Gale SW Cribb PJ Ormerod P (2009) Subfam. Orchidoideae. In: Wu ZY Raven PH Hong DY (Eds) Flora of China (Vol. 25). Science Press, Beijing, Missouri Botanical Garden, St. Louis. Beijing: Science Press and St. Louis: Missouri Botanical Garden Press, 119–124.

[B6] Claessens J Kleynen J (2016) Orchidées d’Europe: fleur et pollinisation. Biotope éditions, Mèze. [trad. T. Pain]

[B7] Duthie JF (1906) The orchids of the north-western Himalaya. Annals of the Royal Botanic Gardens, Calcutta 9: 81–211.

[B8] Efimov PG (2016) A revision of *Platanthera* (Orchidaceae; Orchidoideae; Orchidinae) in Asia. Phytotaxa 254(1) 1–233. https://doi.org/10.11646/phytotaxa.254.1.1

[B9] Hara H Stearn W Williams L (1978) An enumeration of the flowering plants of Nepal. Trustees of the British Museum (Natural History), London.

[B10] Hooker JD (1890) The Flora of British India (Vol. 6 part 17). L. Reeve, London http://dx.doi.org/10.5962/bhl.title.678

[B11] Hunt P (1970) Notes on Asiatic Orchids: V. Kew Bulletin 24(1): 75–111. https://doi.org/10.2307/4103252

[B12] King G Pantling R (1898) The orchids of the Sikkim-Himalaya. Annals of the Royal Botanic Gardens, Calcutta 8: 1–342. [t. 1–448]

[B13] Kurzweil H (2010) Taxonomic studies in the genus *Peristylus* (Orchidaceae) in Thailand. Nordic Journal of Botany 28: 21–46. https://doi.org/10.1111/j.1756-1051.2009.00373.x

[B14] Kurzweil H Lwin S (2014) A Guide to Orchids of Myanmar. Natural History Publications, Borneo, 196 pp.

[B15] Lang KY (1988) The Genus *Herminium* Guett. (Orchidaceae) in China. Acta Phytotaxonomica Sinica 26(3): 173–188.

[B16] Lee J Kim SN Kim M (2013) First record of *Androcorys pusillus* (Orchidaceae) from Mt. Baekdu. Korean Journal of Plant Taxonomy 43(1): 27–29. https://doi.org/10.11110/kjpt.2013.43.1.27

[B17] Nilsson LA (1979) Pollination ecology of *Herminium monorchis* (Orchidaceae). Botaniska Notiser 132: 537–549.

[B18] Ormerod P (2013) Orchidaceous additions to the floras of China II. Taiwania 58(1): 20–34.

[B19] Pearce N Cribb P Renz J (2001) Notes relating to the Flora of Bhutan: XLIV – Taxonomic notes, new taxa and additions to the Orchidaceae of Bhutan and Sikkim (India). Edinburgh Journal of Botany 58: 99–122. https://doi.org/10.1017/S0960428601000488

[B20] Pearce N Cribb P (2002) Flora of Bhutan (Vol. 3). Royal Botanic Garden (Edinburgh) and Royal Government of Bhutan.

[B21] Pradhan UC (1976) Indian Orchids: Guide to Identification and Culture (Vol. 1). Self Published, Kalimpong, 747 pp.

[B22] Press JR Shrestha KK Sutton DA (2000) Orchidaceae. In: Press JR Shrestha KK Sutton DA (Eds) Annotated Checklist of the Flowering Plants of Nepal. The Natural History Museum, London.

[B23] Pridgeon AM Cribb PJ Chase MW (2001) Genera *Orchidacearum* (Vol. 2) – Orchidoideae. Oxford University Press, 464 pp.

[B24] Raskoti BB Jin WT Xiang XG Schuiteman A Li DZ Li JW Huang WC Jin XH Huang LQ (2016) A phylogenetic analysis of molecular and morphological characters of *Herminium* (Orchidaceae, Orchideae): evolutionary relationships, taxonomy, and patterns of character evolution. Cladistics 32(2): 98–210. https://doi.org/10.1111/cla.12125 10.1111/cla.12125 34736301

[B25] Raskoti BB (2012) A new species of *Bhutanthera* (Orchidaceae; Orchidoideae) from Nepal. Phytotaxa 62: 57–60. https://doi.org/10.11646/phytotaxa.62.1.10

[B26] Raskoti BB (2013) A new species of *Herminium* (Orchidoideae, Orchidaceae) from Nepal. Phytotaxa 98(1): 23–26. https://doi.org/10.11646/phytotaxa.98.1.3

[B27] Seidenfaden G (1977) Orchid genera in Thailand V. Orchidoideae. Dansk Botanisk Arkiv 31(3): 1–149.

[B28] Tang T Wang FT (1940) Contributions to the knowledge of eastern Asiatic Orchidaceae. Bulletin Fan Memorial Institute of Biology Botany 10: 28–31.

[B29] Tang T Wang FT (1937) Notes on Orchidaceae of China II. Bulletin Fan Memorial Institute of Biology Botany 7: 127–144.

